# Blodgett's (1919) “Ship camouflage” 105 years on: A misperception of dazzle perception revealed and redressed

**DOI:** 10.1177/20416695241312316

**Published:** 2025-03-14

**Authors:** Timothy Simon Meese, Samantha Louise Strong

**Affiliations:** 1722 Aston University, UK

**Keywords:** depth and 3D perception, dazzle camouflage, direction perception of scale models, World War 1 (WWI), spatial cognition, spatial vision

## Abstract

During WWI, dazzle camouflage involved painting allied shipping with bold geometric patterns to disrupt the perceptions of enemy submariners. The first experiment to provide quantitative results on this (Blodgett, 1919; *MIT Libraries*, MA) used scale models and mechanical simulation, and reported enormous perceptual errors for their perceived direction of travel (up to ∼60°), possibly due to a “twist” effect from forced perspective. However, Blodgett's work did not meet modern standards and the organisation of his report complicates evaluation. Here, we produce (i) reformatted and (ii) heavily edited versions of the original report to improve readability, and (iii) provide a critical reappraisal of the work including (iv) a detailed reanalysis of Blodgett's data and (v) a new control experiment on edited images of the original stimuli. After addressing problems with Blodgett's analysis and control experiment, we found results indicating a twist of only about 7°, but a much larger “hysteresis” effect (∼19–23°) where perceived direction was drawn to the horizon regardless of dazzle. This effect combined both constructively and destructively with “twist”, depending on the direction of the target ship. These reappraised findings resolve an apparent conflict with the second quantitative experiment on dazzle ships conducted over a century later using computer displays online (Lovell et al., 2024; *Royal Society Open Science*). We conclude that Blodgett's approach and data remain of interest today, but his conclusions substantially overestimated the effectiveness of dazzle camouflage in biasing the perceived directions of ships. However, other potential benefits of dazzle, including perceptual variance, await systematic investigation.

## How to cite this article

Meese, T. S., & Strong, S. L. (2025). Blodgett's (1919) “Ship camouflage” 105 years on: A misperception of dazzle perception revealed and redressed. *i–Perception*, *15*(0), 1–31. https://doi.org/10.1177/20416695241312316

If any real-world object should ignite the passions of a visual psychophysicist beyond that of a zebra, it has to be the dazzle ship. Anon (2024)

**Figure 1.**
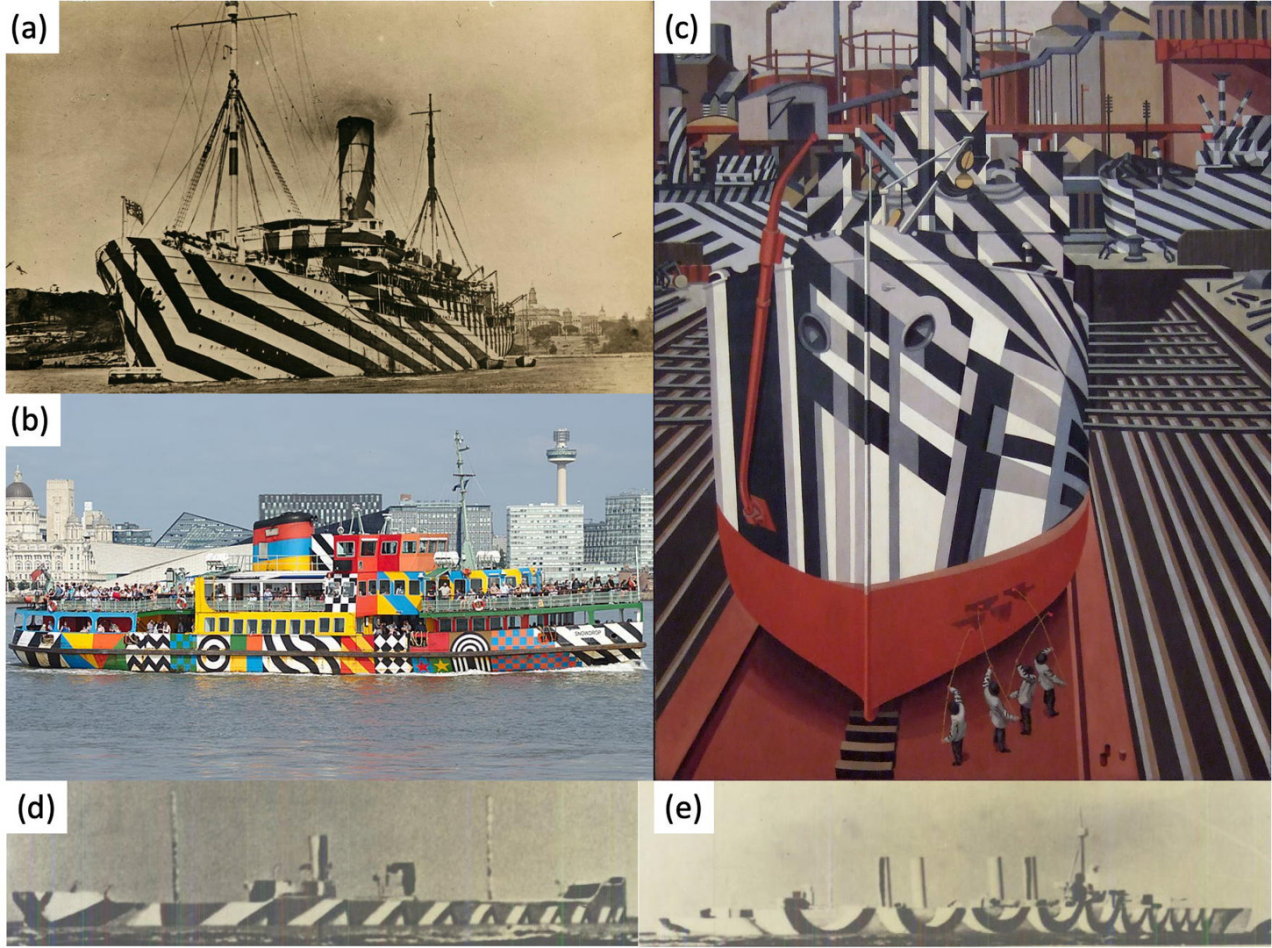
Examples of dazzle ships. (a) Passenger ship, SS Zealandia. Wikimedia Commons dates this as 1914, predating the use of dazzle camouflage; a date of 1918 seems more likely, since this is when she was reportedly requisitioned as a WWI troopship (“SS Zealandia (1910)”, n.d.). (Photographer: unknown; Australian National Maritime Museum, public domain). (b) Mersey ferry, Snowdrop, 25 August 2021. (Photographer: Rodhullandemu). (c) *Dazzle-ships in dry dock at Liverpool*, Edward Wadsworth, oil on canvas, 1919. (Photographer: Mzajac; public domain). (d, e) Model ships from between the wars demonstrating the use of forced perspective. Both photographs are broadsides (target angle = 90°), but the bow (to the right) appears to point away from the observer. As far as is known, these designs were not applied to real ships. (Photographer: unknown; public domain.) Images in (a), (b), and (c) were sourced from Wikimedia Commons. Panels (d) and (e) are from Bittinger and Hulburt (1936a). Panels have been cropped to fit the montage.

As a defence against enemy submarines in World War I, American and British authorities painted troop ships, merchant ships, and their escorts in ‘dazzling’ patterns of high-contrast geometric shapes (e.g., Figure 1(a)). (For reviews, see: Behrens, 1999, 2016; Bekers et al., 2016; Covert, 2007; Forbes, 2009; Merilaita et al., 2017; Scott-Samuel et al., 2011; Taylor, 2016; Wilkinson, 1969; Wilkinson, 2019; Williams, 2001, 2022). It is impossible to achieve consistent invisibility of a ship at sea using paint (Bittinger & Hulburt, 1936b; Van Buskirk, 1919a; Wilkinson, 1917, 1919a), not least because of the natural variations under lighting, weather and sea conditions that occur over space and time (Bittinger & Hulburt, 1935; Van Buskirk, 1919a, p. 183). Other methods—and several were tried (DuBose, 1937, pp. 33–38; Van Buskirk, 1919a)—also met failure. Instead, the principal idea of dazzle was to use camouflage to disrupt (Thayer, 1909, 1918; Warner, 1918) the enemy's perception of speed, direction and possibly ship type (The National Archives of the UK (TNA), 1917; Blodgett, 1919; Van Buskirk, 1919a; Wilkinson, 1919a, 1969; see also Behrens, 1999, 2012), thereby corrupting the aiming solution for firing torpedoes. The invention of this so-called dazzle camouflage (defined as “…painting a ship in such way as to produce confusion or deception” (DuBose, 1937, p. 15)) is typically credited to the English artist, Norman Wilkinson, though in more recent years, a case has been made for the Scottish scientist, Graham Kerr (Evans, 2015; Forbes, 2009; Murphy & Bellamy, 2009; Taylor, 2016). Nonetheless, it was Wilkinson who was placed in charge of the Naval Camouflage Unit, housed in the Royal Academy of Arts. In a letter (dated July 16, 1917) to the Rear Admiral Clement Greatorex of the Royal Navy for the attention of the US Navy Board, Wilkinson declared that

…the only course open is to paint [each ship] in such a way as to deceive the attacker as to her size and course; this can only be done by extreme contrasts of colour and shapes, which will so distort the vessel as to destroy her general symmetry and bulk” (Van Buskirk, 1919a; Wilkinson, 1917, 1969)

The remarkable results of this innovative approach have long captured the public's imagination. This is evident in various forms of visual art from the day (Figure 1(c); Evans, 2015; Taylor, 2016), publicity posters for the Cunard Line (see Meese, 2015), and even British bathing suits of the era (Behrens, 2012, p. 309; Taylor, 2016, p. 106). The legacy continues in modern-day artwork (Evans, 2015; Meese, 2015; Taylor, 2016) including the painting of pleasure ships (Figure 1(b)) and even a pop music album by OMD (1983).

Imaginative and delightful as the various camouflage designs for shipping might have been (Hurst, 1919; Skerrett, 1919; Taylor, 2016), a central question was whether they achieved their objective. Securing a definitive answer to this has not been easy for anyone (e.g., DuBose, 1937). Both the British and the US developed test facilities for camouflaged scale models during WWI (Jones, 1919; Van Buskirk, 1919b; Yates, 1919; see also: Bekers & De Meyer, 2017; Blodgett, 1919; Wilkinson, 1919a; Williams, 2022), and several relevant declassified naval documents are now readily available in the public domain (Bittinger & Hulburt, 1935, 1936a, 1936b; DuBose, 1937; ONI, 1918a, 1918b, 1918c, 1918d). However, none of these, nor the various reports by Wilkinson (Wilkinson, 1919a, 1919b, 1922, 1969) or other reports from around that time (Behrens, 2012; Van Buskirk, 1919a) provide a quantitative analysis of the effects (in the public domain at least), relying instead on subjective or speculative assessments and/or anecdotal observations (Williams, 2001), albeit, some of them rather compelling (e.g., Bement, 1919a; Skerrett, 1919; Twombley, 1918; Van Buskirk, 1919a, pp. 112, 294; Forbes, 2009; Wilkinson, 1969, pp. 96–98; Yates, 1919).

In post-war evaluations, the US and the British came to very different views about the value of dazzle (Bittinger & Hulburt, 1936a). The British declared there was no clear evidence that dazzle painting had ever confused the enemy (TNA, 1918; see also: Behrens, 1999; Bittinger & Hulburt, 1936a; Murphy & Bellamy, 2009), while the US celebrated the oft reported statistic (Behrens, 1999; Covert, 2007; Van Buskirk, 1919b; Williams, 1989; “Razzle Dazzle”, n.d.) that only 1% of their ships treated this way had been sunk (Van Buskirk, 1919a, p. 141), though a comparison with untreated vessels was not made. From assessments on the runup to WWII (Bittinger & Hulburt, 1935, 1936a; DuBose, 1937), it is evident that the US was persuaded by the potential benefits of dazzle—despite some misgivings (Van Buskirk, 1919a, p. 305)—but, again, there was no quantitative analysis. In fact, the prevailing American view towards the end of WWI (Bement, 1919b; Van Buskirk, 1919a, p. 151; Warner, 1919a, 1919b) indicates that much attention was being given to false perspective (Behrens, 2016; Blodgett, 1919)—or forced perspective^1^ as it has come to be known (e.g., Papathomas et al., 2012)—where converging lines of linear perspective (Andersen et al., 1998; Bates, 1918, p. 50) or texture gradients (e.g., Braunstein & Payne, 1969; Meese & Holmes, 2004; Niimi, 2023) of various designs and persuasion were painted on the side of the ship (e.g., Lovell et al., 2024; Figure 1(d) and (e)). In this respect, designs with gradients to the bow that twist the ship away from the observer, rather than towards, were preferred (Bittinger & Hulburt, 1936a). Put in the right hands and context, this general approach—contravening physical 3D with pictorial 3D—is known to produce compelling perceptual distortions (e.g., Papathomas et al., 2012; Rogers & Hughes, 2023).

One early report that provided a quantitative analysis of perspective-style dazzle designs was that of Blodgett (1919) in his Bachelor of Science thesis at the Massachusetts Institute of Technology (MIT). This was conducted using reportedly state-of-the-art (for its time) mechanical simulation equipment with painted scale models of ships and devised by the Boston Camouflage District.^2^ Whether his results and conclusion were known to later naval authorities (Bittinger & Hulburt, 1936a; DuBose, 1937) is unclear^3^, but the substantial misperception of dazzle ship directions that he reported for his expert observers (up to ∼60°) would have been encouraging if it were. Certainly, Blodgett's work has attracted attention from modern-day authors (Behrens, 2012, p. 285, 2013, 2016, 2018; Bekers & De Meyer, 2017; Bekers et al., 2016; Lovell et al., 2024; Scott-Samuel et al., 2023; Taylor, 2016, p. 123; Williams, 2022) not least because it is the earliest known record of a quantitative experiment involving the effects of dazzle camouflage on the perception of target direction. (There are several reports of systematic observations of dazzle treated model ships (Bates, 1918; Van Buskirk, 1919a; Wilkinson, 1919a), but no quantitative data from those.) The second such investigation (using online computer displays) did not appear until over one hundred years later (Lovell et al., 2024; Meese et al., 2022).

Unfortunately, the formatting, style and organisation of Blodgett's (1919) original type-written report make it difficult to evaluate on casual reading. We address this here in two ways. First, as a courtesy to the interested reader, we provide two reworkings of Blodgett (1919) in Supplementary Material 1 and 2. One (S1) is a light touch using a contemporary typeface and formatting with the addition of some sub-headings to aid navigation, the aim being to leave the original wording substantially intact. The other (S2) involves much heavier editing and reorganisation of content to approach modern standards for a scientific report including the appropriate use of tense and a focussed methods section (see front pages of S1 and S2 for further details of the reformatting and editing). Second, we critically reappraise the methods, analysis, and conclusions of Blodgett (1919) to provide a much clearer account of what he found. As we shall see, this uncovered both shortcomings and surprises. Most of the problems were addressed by our detailed reanalysis of Blodgett's data, but one required us to run a new control experiment using variously edited images from the original report.

The outcomes of all this are remarkable. First, Blodgett's results are in fact consistent in overall magnitude with later and (as we shall see) more modest findings of Lovell et al. (2024). However, second, hidden in Blodgett's data were the telltale signs of the same two components of misperception identified by Lovell et al. (hysteresis and twist; see later), and, remarkably, in a similar ratio.

## Introduction and Background to Blodgett 1919

Nothing is known to us about Leo Blodgett other than what can be gleaned from his publicly available thesis,^4^ accessible from the MIT library in electronic form (Blodgett, 1919). However, we can say that the work appears earnest and well-motivated, and respectful of many of the people involved in the dazzle projects, including Lieutenant-Commander Wilkinson (RNVR). Although WWI had ended at the time Blodgett carried out his work, there remained the academic question of whether the false perspective dazzle scheme was effective, and this is what he aimed to address: “All experimental work [in] this thesis [was] based upon principles of distorted perspective …” (Blodgett, 1919). It seems Blodgett had access to good resources including, he claimed, almost all relevant data compiled on camouflage from the USA and Europe. He described this as “… an uncoordinated mass and most of it without practical value in developing the subject,” and he presented his thesis without any formal referencing. However, based on his content, it seems he had access to Bates (1918) and Jones (1918) at least (quoting twice from Bates). In places, Blodgett is rather scathing of the efforts by some of the artists involved and it seems this is not without some justification (Behrens, 1999; Van Buskirk, 1919a, p. 104; Warner, 1919a). Since, “… it is not essential to blotch colo[u]rs on the side of the ship in contrasting masses in an artistically hit or miss way,” Blodgett claimed that “[a]n effort was made [(in his thesis)] to reduce the matter to a mathematical basis,” but he presented no such maths, and it seems likely that the term was intended to refer instead to the systematic application of false perspective principles. Even so, while these were described in general and thoughtful terms (see Methods and design descriptions in S2), no formal method was derived for producing dazzle camouflage (his general descriptions are not unlike the report by Bement (1919b) from the time) nor was any formal attempt made to assess the relative merits of the various perceptual factors involved in the designs (Bittinger & Hulburt, 1936a made similar points about the official US WWI designs.). In short, Blodgett quantified the misperceptions of model ship directions for several different dazzle designs of his own (including structures and colours) using principles gleaned from the US Navy Department, reportedly in the final months of WWI. The precise details of these principles (even if any formal expression of them existed), remain unclear to us.

Blodgett's work was performed under the guidance of Professor C. H. Peabody (“Cecil Peabody”, n.d.) of the Department of Naval Architecture and Marine Engineering at MIT. Whether this is the same individual as one of the participants in the experiment (Observer 2) who provided “valuable suggestions [on] the progress of the work” is unclear.

While Blodgett seemingly recognised that one benefit of dazzle camouflage was the disruption of outline and internal structure (e.g., Thayer, 1918; Warner, 1918; see also, Stevens et al., 2006), which could impede identification of class (and hence speed), he restricted his experimental work to assessing the perceived direction of heading (i.e., the perceived direction/angle of a target ship).

## A Critical Reappraisal of Blodgett 1919

### Methods

A full account of the methods derived from Blodgett (1919) is available in S2. Here, we provide an overview, identifying various key features and shortcomings along the way (Blodgett, 1919).

#### Equipment, Task, and Procedure

**Figure 2.**
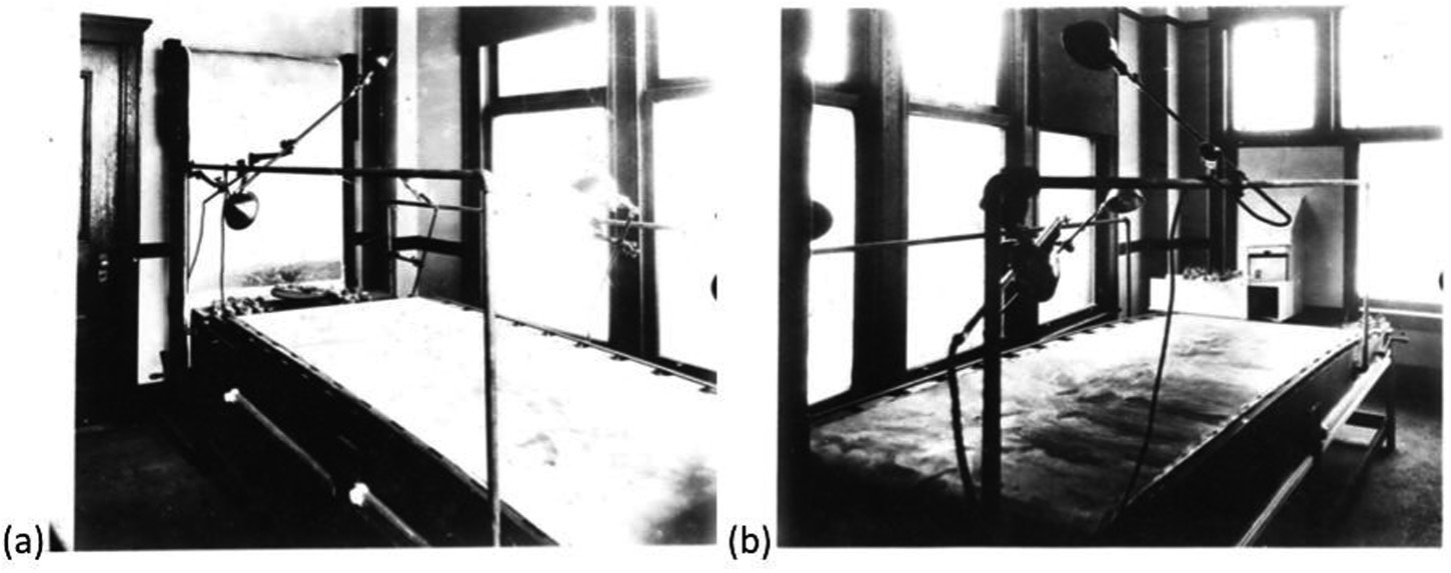
Two views of the equipment from the Boston Camouflage District (BCD) used in the experiment. (This atribution is the best that can be discerned from the original document but uncertainty remains over where the experiments were conducted; Williams (2022, p. 141) indicates that the equipment was donated to MIT by the BCD.) Note the horizontal belt used to simulate sea and in (a), the vertical belt used to role one of four different skyscapes into place. Note also the hand crank in the view in (b), towards the rear of the belt. From Blodgett (1919).

The equipment used in the experiment is shown in Figure 2. Model ships (examples can be seen at one end of the conveyer belt in Figure 2(a)) were viewed from the far end of the horizontal belt through a periscope. Participants set their perceived direction for the target ship using a dial (i.e., they performed a direction matching task). Precise details of this and what took place between experimental trials (including the order of conditions) are not described, though the dial indicating the true direction of the ship was mounted at the periscope end of the viewing theatre. Presumably, this was not inspected by the participants. Viewing time was probably unlimited, but this is not entirely clear (see the *Design, participants, and more procedure* section in S1).

#### Stimuli: Skyscapes, Seascapes, and Weather

There were four different skyscapes (clear blue, hazy, stormy, and shoreline) and four different seascapes (calm and blue, slightly ruffled and green, dull and hazy grey, and rough white capped and choppy) though Blodgett claims that “[a] little too much was left to the artistic temperament and imagination of [the artists involved].” There was an attempt to match seascapes and skyscapes realistically (see Table 1 in S2), but it appears that these arrangements were not systematically counterbalanced across other conditions. A device to simulate fog of various densities was also used. This involved a semi-transparent mirror placed close to the periscope aperture in conjunction with blue and ground glass filters. Nitrogen daylight lamps were also used to produce various lighting effects. None of these factors were varied systematically but (it seems) distributed somewhat randomly across experimental trials under the following constraints: no two scenes were to be alike (whether this was per participant or across the entire experiment is not clear), but each participant was to have systematic exposure to each of the four skyscapes. Blodgett did not report specific details on how the various weather manipulations might have impeded visibility, but some comments on weather at sea are provided by Bates (1918, pp. 17–18) and Jones (1918, pp. 57–58). In short, weather conditions served as an uncontrolled source of variance in Blodgett's experiment.

#### Stimuli: Target Ships

**Figure 3.**
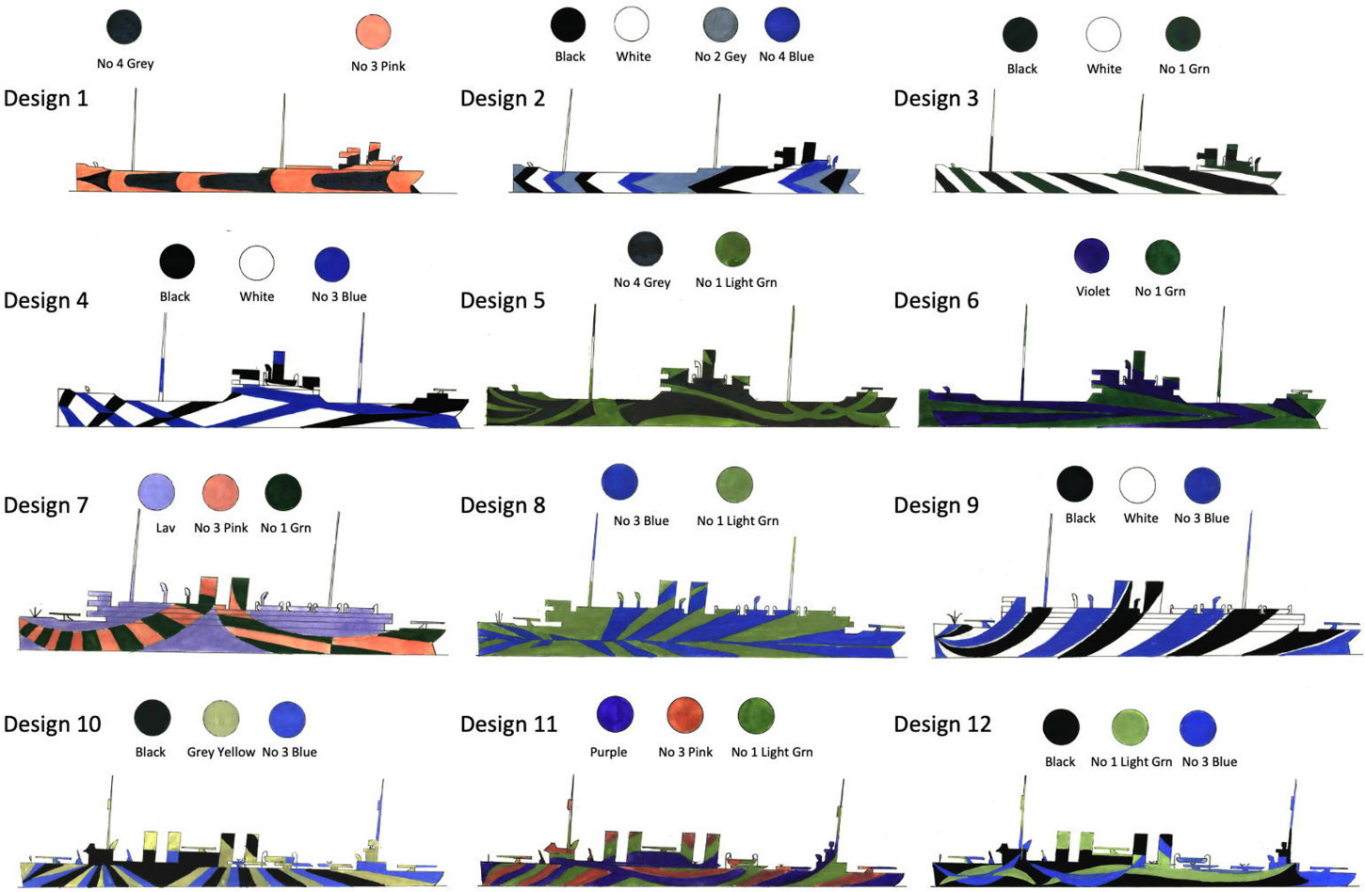
The 12 camouflage designs distributed across four (model) ship classes. From top to bottom these are: tramp 1 (superstructure aft), tramp 2 (superstructure amidships), passenger/troop carrier, destroyer. The ships are all shown with a direction of −90 (270) deg. Abbreviations for colours: Grn = green; Lav = lavender. See S1 and S2 for further details on colours. Adapted from Blodgett (1919).

The 12 camouflage designs for which formal perceptual data were gathered are shown in Figure 3, distributed evenly across four different ship classes (i.e., ship class and camouflage design were confounded). These colour images are copied from the MIT library pdf of Blodgett (1919), though Behrens (2013) reports that the originals were painted in gouache and that some of the colours might have changed over time (see the Part 2: Analysis of Individual Trials Across Four Different Factors section for further discussion).

Only one side of the ship was shown by Blodgett. Since no mention was made of the other side, we assume it was the same on both sides relative to the bow, consistent with comparisons between Blodgett's drawings (Figure 3) and some of his photographs (S1 and S2). For fuller descriptions and discussions of each of the designs in Figure 3, see S2.

Blodgett did not report the size of his model ships, but they were built to a scale of 1/32":1 ft (1:384). Around this time, destroyers were between 165 and 300 ft (50–91 m) in length (“Destroyer”, n.d.), tramps (common cargo ships) were between 250 and 400 ft (76–122 m), and passenger ships between 450 and 1000 ft (137–300 m) (ONI, 1944). This suggests a potential range of 5.2″–31.3″ for the scale models, with averages of 7.3″, 10.2″ and 22.7″ for destroyers, tramps and passenger ships, respectively (18.5, 25.9, and 57.7 cm). In their qualitative observations between the wars, Bittinger and Hulburt (1936a) used a viewing theatre with a description similar to the one used by Blodgett and report their scale models to be around 14″ (35.6 cm) in length.

#### Participants and Number of Trials

Blodgett reported that six observers took part in the experiment. Observers 1 and 2 were both familiarised with the stimulus designs (the painted model ships). Observer 1 was an (unnamed) experienced Lieutenant in one of the European Navies, familiar with ships, periscopes and conditions at sea. Observer 2 was “… closely in touch with the evolution and application of each [stimulus] design, [making] repeated observations [of] them, [and offering] valuable suggestions [during] the progress of the work.” Observers 3 to 6 were naive to the camouflage designs, though familiar with the structures of ships, conditions at sea, and the principles of perspective and visual illusions.

In Blodgett's results tables there are entries for “First Observer,” “Second Observer,” and “Third Observer.” He did not make clear what he meant by third observer, but his earlier text implies that observers 4–6 were treated as a group. We assume that “Observer 3” refers to this group and we have used the label, “Observers 3–6,” accordingly. This was our best guess, but it is possible that the results for Observers 4–6 were discarded. Blodgett provides no independent presentation of the results (in any form) for Observers 3–6, making it impossible for us to perform independent analysis and/or data cleaning on them.

The perceived directions for each of two physical directions for each of the 12 camouflage designs (Figure 3) and under each of the four different skyscapes were measured for each of the six/three observers with sea and lighting conditions varying across them. This gave 2 × 12 × 4 = 96 trials (ship presentations) per observer.

The 96 perceived direction settings and the corresponding actual directions are tabulated in Blodgett (1919). They are transcribed here in S1 and S2 and our transcription of these data and the results of our cleaning—see below—are placed in publicly available editable files (see end matter). Blodgett (1919) did not report how the physical directions were selected for each condition, though our analysis of his tabulated data shows they were not evenly distributed around the compass. Instead, it seems they were pseudo-randomly selected from spreads (SD = ∼8.5°) around four nominal centres of ∼±35° relative to East (90°) and West (270°) for an observer facing nominal North through the periscope, but physical direction was not counterbalanced across camouflage design (see Supplementary Material 3 (S3)).

#### Simulated Viewing Distances

The simulated distances were 1,100 yards (1,000 m) for four observers and 2,200 yards (2,000 m) for the other two, but which observers were assigned to each distance is not reported. From what Blodgett (1919) says on p. 14 in S1, the ship wearing Design 5 might have been presented at a simulated distance of 3,000 yards (2,700 m), but this is not clear.

#### Control Conditions

A uniform white and black background were assumed to produce zero errors. In the case of white, Blodgett (1919) claimed this was borne out by experiment, though no details (e.g., number of trials or observers) were provided. Furthermore, there is no evidence (or even a suggestion) that the black condition was run. Although these two skyscapes contributed to each of his 12 data figures (see S1 and S2), Blodgett placed little emphasis on them, and we do not discuss them further.

Results were also reported for ships painted uniform black and uniform grey (e.g., see Table 2 in S2). These conditions were not mentioned by Blodgett until his results, and it is not clear under what seascape and weather conditions these controls were run, how many trials were performed, what the true directions were, or which or how many participants took part. In fact, there is nothing in Blodgett's report to suggest that these conditions were performed by any of the six participants mentioned above.

### The Meaning of Target Angle (Ship Direction)

Referring to the direction (or equivalently here, the angle) of a ship in open sea poses the problem of identifying a framework against which that direction should be judged. Furthermore, different authors have reported on this in at least two different ways. Before elaborating on this, it is important to note that under both conventions we are considering the instantaneous direction of the ship from the viewpoint of the submarine, not from the bridge of the ship.

**Figure 4.**
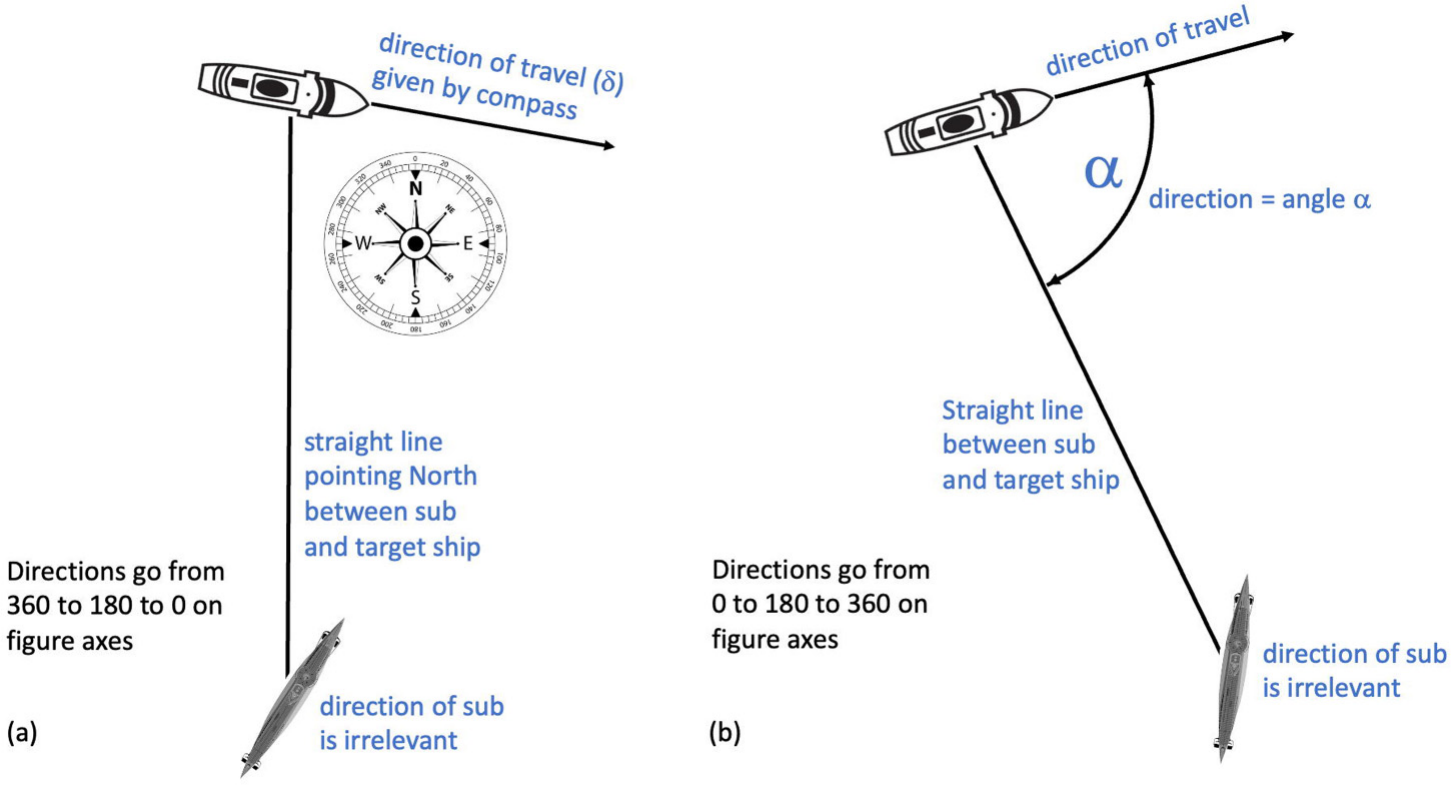
Two conventions for reporting the direction/angle of a target ship. (a) An absolute convention (compass angle) where the ship's direction (*δ*) is judged by the angle of a compass (North = 0°) (e.g., Lovell et al., 2024). For the purposes of experimentation, it is assumed that the observer is looking directly North through the periscope (i.e., the direction of regard is 0°). (b) A relative convention (target angle) where the compass is irrelevant and direction (*α*) is given by the (counterclockwise) angle between the direct line of sight from the observer (e.g., looking through a periscope) and the line of travel for the ship (e.g., Meese et al., 2022). Note that in both conventions, the direction of the submarine from which the observations are being made is irrelevant.

One natural predisposition to the direction problem is to use a concrete reference frame given by the compass, where North = 0° (e.g., Lovell et al., 2024). This is shown in Figure 4(a). The ship's direction (*δ*) is given by reading off the compass directly. In this example, *δ* is a little over 90° (East), probably about 100°. One problem with this compass-based approach is that the interpretation of *δ* depends on the direction of regard (in this conveniently simple example, 0°; the direction of the straight line between the submarine and the ship). This problem can be circumvented (to provide generality in the context of an experiment) by declaring that nominal North is always the direction of regard. This is to rotate the compass in Figure 4(a) to parallel any change in the absolute direction of regard should that be manipulated (though the angle of regard was not manipulated by either Blodgett (1919) or Lovell et al. (2024)). Under this compass-based convention, the meaning of direction (compass angle, *δ*) for cardinal examples is shown in the lefthand column of Table 1.

**Table 1.** Meaning of target ship direction (angle) for each of the two conventions in Figure 4. The observer is positioned in the submarine and is looking towards the target ship. The entries in the table are relative to this observer. In general (assuming the line of sight is nominal North), *α* = 180 − *δ*.

Direction (*δ* or *α*)	Target ship's direction of travel relative to the observer	
	Compass angle (*δ*): Figure 4(a)	Target angle (*α*): Figure 4(b)
0°	Directly away	Directly towards
90°	Right angles to the right	Right angles to the right
180°	Directly towards	Directly away
270° (−90°)	Right angles to the left	Right angles to the left

A more elegant solution for reporting ship direction in our present context (and adopted by Meese et al., 2022) is shown in Figure 4(b). In this convention, the compass is irrelevant. For any ship and submarine positioned anywhere at sea, there is a straight line that joins them (this is key to the approach). The ship's direction, angle α, is the (counterclockwise) angle between that line and the direction in which the target ship is travelling. This is sometimes referred to as the target angle. In this example, *α* is a little less than 90°, probably about 80°. Under this target angle convention, the meaning of direction (α) for cardinal examples is shown in the righthand column of Table 1. Note that in general (assuming the generality rule for the compass convention applies), *α* = 180 − δ. Importantly, both conventions agree on what is meant by the direction of a target ship (defining it relative to the observer's line of sight) but disagree in general on the numerical reporting of those directions (see Table 1).

**Figure 5.**
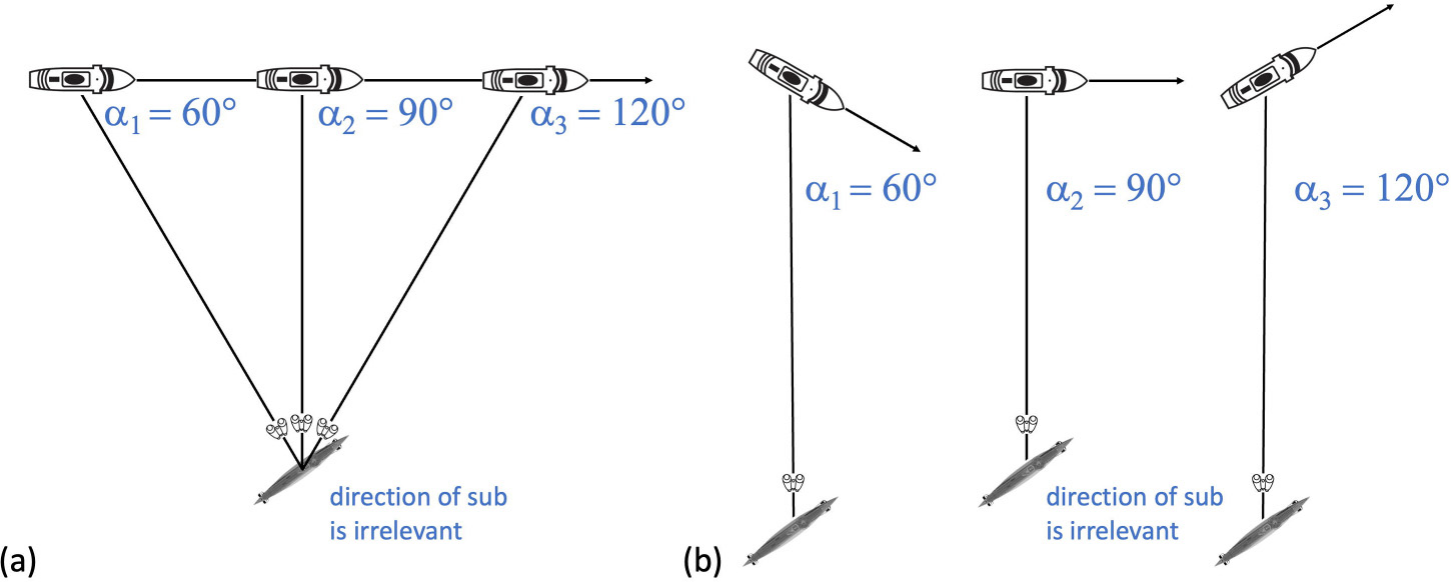
The positions of the ship and the submarine are irrelevant for the reporting of target angle. Wherever they are placed, the ship is always straight ahead relative to the observer's line of regard (denoted by the direction of each binoculars icon). (a) A stationary submarine and a ship that changes position from left to right on a fixed heading. (b) A stationary submarine and a ship on three different headings. Ignoring the irrelevant direction of the submarine, the three situations in (b) are simply rotations on the page of the three situations in (a). This means the target angles from left to right are the same in (a) and (b).

To quash potential confusion for the reader, one further point is made in Figure 5. This shows that we do not need to consider the positions of either the submarine or the target ship. In Figure 5(a), the ship is on a fixed course moving from left to right. Three specific positions of the ship are shown and the target angle (α) for each. Note also how the distance to the ship varies from left to right. In Figure 5(b), the three target ships are in the same position (though not at the same distances from the submarine) but on different headings. The relative geometry between submarine and target ship (including target angle) are identical across Figure 5(a) and (b). In short, the reason why position does not represent an extra degree of freedom is because the ship is always straight ahead (in all six panels of Figure 5 and any other possible arrangement) from the observer's point of view.

The choice of reporting target angle (α) (Figure 4(b)) over compass angle (δ) is to be preferred for three reasons. First, it is naturally general in that the relative positions of the target ship and the submarine are built into a single parameter without further contrivance. Second, when plotting perceived direction against actual (physical) direction (both of these from the perspective of the submarine), it is intuitive to organise the two-dimensional graphical space so that a vertical track through that space represents a change in perceived direction away from the observer (i.e., up is away) and, for consistency, that a track to the right through that space represents a change in actual direction away from the observer (i.e., right is away). To achieve this using compass angle (Figure 4(a)), it is necessary to invert both the *x*- and *y*-axes of the plot (Lovell et al., 2024), whereas no such inversion is necessary when using target angle (Figure 4(b)). These axis inversions can be seen in some of our later graphical presentations of Blodgett's results where we illustrate both conventions in the same plot for completeness. Third, it is the convention used in naval reports of sea tests (e.g., Bittinger & Hulburt, 1936b).

#### Mirror Reversals and Angle on the Bow

A further point about data presentation concerns mirror pooling around the clock. Consider the geometry of a ship on the eye, including its camouflage details and assuming the same camouflage on each side of the ship relative to the bow.^5^ A ship at a target angle of −70° (a counterclockwise angle, α, of 290°) is a simple mirror reversal of a ship at a target angle of 70°. If the first condition was subject to an illusory perceived twist in direction away from the observer of, say, 10°, giving a perceived target angle of −80°, then it follows by geometry that the expected perceived direction for a target angle of 70°, would be 80°. In other words, in principle, we are safe to pool experimental results over mirror reversals where target angle is expressed between −180° and 180° so long as we flip the negative sign. The result is an angle between 0° and 180°, sometimes referred to as angle on the bow (“Target angle”, n.d.; Florek, 2017c). We present (folded) results this way in several of our forthcoming plots, but always we have done this for the inferential statistics to maximise the power available for the analysis.

#### What Does all This Mean for Blodgett?

Unfortunately, ship angle (what we typically refer to as direction) was not defined by Blodgett (1919). However, Blodgett did not manipulate the direction of regard, nor did he plot his results in a two-dimensional direction space, so it is unlikely that the shortcomings of using compass angle would have presented themselves. We also suppose that the most natural way to construct the equipment in Figure 2 would be for the perceived and physical direction dials to be consistent with the compass (i.e., the lefthand column in Table 1) and note that Blodgett refers to an “indicator on a compass card”. Therefore, we assume (Participants and Number of Trials section) that the convention used by Blodgett is the one in Figure 4(a) (and the same as in Lovell et al., 2024) with observers looking due North through the periscope (Van Buskirk, 1919a, p. 151). In the Blodgett’s Direction Convention section, we consider the consequences of this assumption being wrong and conclude that it is correct.

### Results and Discussion

For the sake of brevity, this section (up to the end of the Part 2: Analysis of Individual Trials Across Four Different Factors section) is an edited version of a more detailed account that can be found in Supplementary Material (SR&D1).

**Figure 6.**
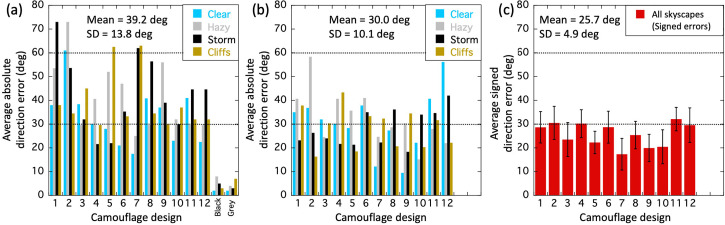
Experimental results (perceived direction errors) averaged across observers. (a) The results of Blodgett (1919) as he reported them (averages of absolute direction errors) for each of the 12 camouflage designs (Figure 3) and the uniform black and grey control conditions for each of the four skyscapes (see legend). Each average was derived from no more than 12 measures: two observations for each of six observers but weighted as if only a total of six observations across three observers. The measures were spread rather haphazardly across physical directions within the constraints described in the Methods section (see S3 for details). (b) The same as (a) after data cleaning but retaining absolute errors in the averaging (see text for details) and with the unsafe control results removed. (c) The same as (b) but using signed errors in the averaging and with results collapsed across the four skyscapes. Error bars are ±1 SE of the signed perceptual error distributions in the pool. The means and SDs reported in each panel are calculated across the full set of bars excluding the black and grey control conditions. The horizontal dashed lines are arbitrary comparison lines at 30° and 60°.

Blodgett's (1919) results for each dazzle design are replotted in Figure 6(a). The ordinate is the absolute (i.e., unsigned) difference between actual direction and perceived direction. The figure shows substantial variation of perceptual errors across conditions, though no obvious systematic effects for dazzle design or skyscape.

Most notable in Figure 6(a) is (i) how large some of the absolute direction errors are (>60°) and (ii) how much greater they are for each of the 12 dazzle designs compared to the two neutral conditions (black and grey at the far right of the plot). However, as highlighted above, the validity of these control results is questionable, and we consider them unsafe. We return to these data in the General Discussion section and again in the Overall Conclusion and a Eulogy for Blodgett section after running our own control experiment (A New Control Experiment section) designed to compare dazzle designs with neutral (grey) versions (cf., Lovell et al., 2024).

### A Revaluation of Blodgett's Results

We identified several sources of error in Blodgett's presentation (see appendices in either S1 or S2 for his data presentation) and worked through correcting each source in turn before inspecting the cleaned results. This procedure was put in place to protect against p-hacking and expectation bias by the current authors through the influence of cleaning decisions.

#### Data Cleaning

We identified five different problems with Blodgett's data and his analysis, four of which are listed in Table 2. First, we found instances where the reported error was inconsistent with the difference between the actual and perceived directions (data column 1, Table 2) and we removed these data from further analysis (these data are identified in the appendices of S1 and of S2).

**Table 2.** Anomalies in Blodgett's results tables (entries are number of trials). The ‘differencing’ column is for when the reported error was inconsistent with the difference between actual and perceived directions. The ‘direction’ error column is for when the error was greater than 90° (see text for details). The ‘missing’ column refers to missing data for trials. The ‘averaging’ column is for when Blodgett's average over the four skyscapes (the results he plotted) was wrong. For differencing and averaging, we worked with a tolerance of 1°. The final column lists the number of trials out of 24 (three observers × four skyscapes × two trials) that were missing or removed (owing to differencing or direction errors) for each camouflage design (i.e., the sum of the first three columns). This totalled 40 over the entire experiment, leaving 248 of 288 trials intact for our analyses. In our own plots (i.e., all replots of Blodgett's data except Figure 6(a)), we discarded Blodgett's averages and recalculated these ourselves.

Design	Differencing/24	Direction/24	Missing/24	Averaging/4	Removals/24
1	1	4	0	3	5
2	0	4	1	1	5
3	2	1	0	2	3
4	0	1	0	1	1
5	0	1	0	1	1
6	1	1	0	1	2
7	3	0	0	3	3
8	0	3	0	1	3
9	4	0	0	0	4
10	4	3	0	1	7
11	3	0	0	3	3
12	1	2	0	1	3
Total	19/288	20/288	1/288	18/48	40/288

Second, we found several cases where Blodgett's averaging across observer was wrong (data column 4, Table 2), but this held no obvious relation to the first problem. Therefore, we disregarded Blodgett's averages and performed these ourselves (we make a further point about averaging below).

Third, we found some cases where the difference between actual and perceived directions was greater than 90° (data column 2, Table 2; see also our archived data files referenced in the end matter). This can happen if the observer misidentifies the stern as the bow, but this is unlikely to persist at sea because of the clear giveaway of a steamship's smoke and the tracking of quarry (e.g., Florek, 2017a, 2017b; ONI, 1918a; Paterson, 2018). This type of perceptual error was also dismissed by Wilkinson, in Van Buskirk (1919a, p. 125). A second way in which perceptual errors can be greater than 90° is when there is a gross perceptual switch from veridicality as to whether the bow or stern is closer to the observer (e.g., a target angle of 90 + 50° is misperceived as an angle of 90–50°). Like above, this struck us as a different class of perceptual error from our main interest, perhaps more akin to the perceptual uncertainty/confusion that we discuss in the Response Variance, Uncertainty/Confusion, Weather, and Experience section. We also note that the simulated fog used by Blodgett might have contributed to both of these situations in an uncontrolled way (by obscuring small but telltale forward and rear facing features of the superstructure, for example). Therefore, we followed Lovell et al. (2024) and removed all results where perceptual errors were greater than 90°. Of the 20 out of 288 trials where we did this (Table 2), 11 were for bow/stern confusions and all but one were for either Observer 1 (*n *= 6) or Observer 3–6 (*n *= 13). For completeness, in Supplementary Material 4 (S4), we reintroduced the 9 trials that were not bow/stern confusions, showing that their exclusion was not critical for our main observer-based conclusions. And like Lovell et al. (2024), neither did we notice anything of interest about the conditions that resulted in data removal.

Fourth, there was one trial in Blodgett's results tables where no data were recorded (Design 2, Observer 2, clear skyscape; data column 3 in Table 2).

The fifth problem was with Blodgett's approach to averaging. He reported perceptual errors as the absolute (unsigned) differences between actual and perceived directions and averaged those, but this runs the risk of amplifying effect size because his results included perceived directions each side of veridicality (e.g., see SR&D1 and S3). This highlights a need for care with the perceptual error sign in our reanalysis.

Finally, we note that the individual data for each of Observers 3‒6 might have been subject to any or all the problems above, but because only their (presumed) average was presented by Blodgett (1919), we could apply corrections only to the combined results.

### A Reanalysis and Discussion of Blodgett's Results

#### Part 1: Following Blodgett

Figure 6(b) shows the absolute (unsigned) errors from Figure 6(a) after all other aspects of data cleaning and with the questionable black and grey control conditions removed from the plot. There was a general reduction in the overall magnitude of perceptual error (by 9.2°), and a reduction in the variance (the SD reduced by 3.7°), but no obvious pattern of perceptual errors emerged. To get an overall picture across the 12 camouflage designs, we collapsed the results across skyscape while respecting the sign of perceptual errors before any of the averaging (see S3 for further details) and plotted these in Figure 6(c) (completing our data cleaning). Still there was little of interest to see, but the overall effect size reduced by a further 4.3°. Inspection of the 12 individual designs (S3) also revealed nothing further about visual misperceptions.

#### Part 2: Analysis of Individual Trials Across Four Different Factors

**Figure 7.**
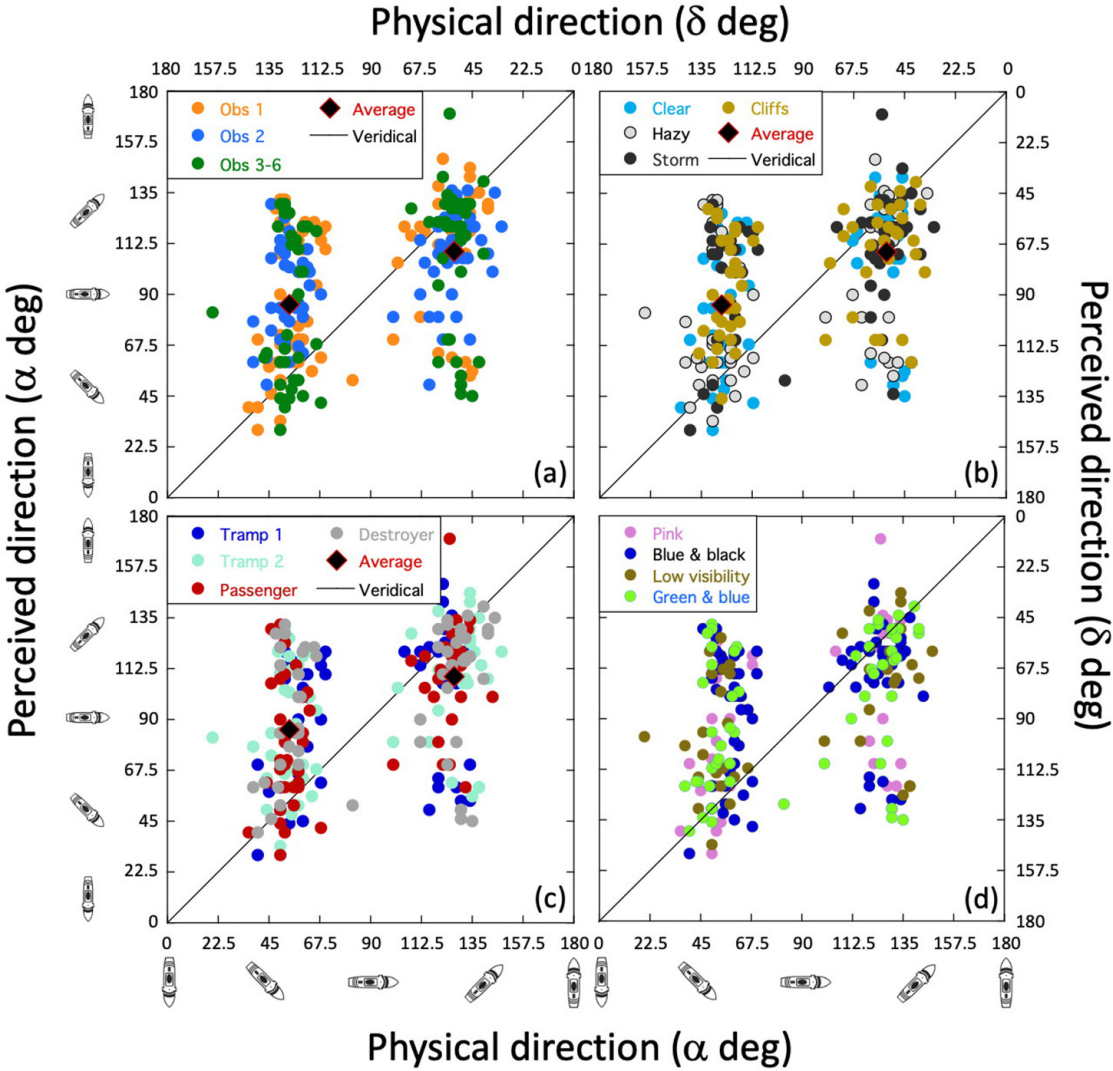
Cleaned results for perceived direction as a function of physical direction. The axis icons show the direction of the target ships relative to the viewpoint of the observer. The labels are for each of the two direction conventions (target angle, *α*, and compass angle, *δ*) described in Figure 4. The diagonal lines in each panel are the contours of veridicality. Deviations of data points from these contours indicate signed perceptual errors (according to the target angle axis). In (a, b, and c), each circle is the result of an individual trial, and all valid trials are plotted (see *Data cleaning*), including mirror reversals (see S3 and Stimuli: the Skyscapes, Seascapes, and Weather section). The black diamonds are (unweighted) averages of the data cluster means across mirror reversals (see S4). The data set is the same across each of these three panels. The symbol colours denote grouping by (a) observer, (b) skyscape and (c) ship class. The results in (d) are for camouflage colour and exclude ship design 11 (see text for details). Within each panel, there were no statistically significant differences for the factors depicted.

Our next step was to perform a detailed analysis at the individual trial level. We did this to investigate potential effects of observer, skyscape, ship class, and ship colour. Although Blodgett (1919) expressed interest in these four factors, he provided no formal comparisons. Figure 7(a) shows perceived direction against physical direction for all cleaned data (subject to mirror reversal as appropriate; see the earlier section), and is colour coded for the three/six observers. The black diamonds show the two cluster averages (see figure caption for arithmetic details). The three observer groups overlapped substantially and *t*-tests (not shown) on each of the three possible comparisons within each of the two clusters of signed errors (six *t*-tests) confirmed there were no significant differences between the observers [no value of *p* (two-tailed) was less than 0.33]. We used this homogeneity to justify pooling experimental trials across observer (treating the three different observer entries as a single random variable) when looking for differences in the other factors below.

Figure 7(b) is the same as Figure 7(a) but colour coded for the four different skyscapes. Again, there was substantial overlap across conditions and *t*-tests (not shown) confirmed there were no significant differences in the six available comparisons within each of the two clusters of signed errors (12 *t*-tests).

Figure 7(c) is the same as Figure 7(a) but colour coded for the four different ship classes. Once more there was substantial overlap across conditions and *t*-tests (not shown) confirmed there were no significant differences in the six available comparisons within each of the two clusters of signed errors (12 *t*-tests).

Finally, Figure 7(d) is similar to Figure 7(a) but colour coded for four different colour combinations in the camouflages. These groupings were determined by our sixteen naive participants after performing the control experiment below (A New Control Experiment section; see also S5 and S6). They were given 12 rectangular pieces of paper, each containing one of the coloured designs in Figure 3, and asked to sort them into four piles, each containing any number of ships but associated by the camouflage colours and shades. They were told to ignore the shape (class) of the ship and the form of the patterns. There was marked consistency across participants for 11 of the 12 designs, and we labelled the groupings that emerged as follows: pink for designs 1 and 7; blue and black for designs 2, 3, 4, and 9; low visibility, for designs 5 and 6^6^; and green and blue, for designs 8, 10, and 12 (see Figure 3 for design labels). Design 11 was omitted because there was inconsistency in its grouping across participants (see Supplementary Material S6 for further details). Figure 7(d) shows substantial overlap across these colour groups and *t*-tests (not shown) confirmed there were no significant differences in the six available comparisons within each of the two clusters of signed errors (12 *t*-tests).

After completing the experiment and analysis above, we became aware of recolored versions of the 12 designs by Blodgett (1919), attributed to Roy Behrens in Williams (2022). The most notable colour difference from Figure 3 was that Design 3, allocated to the blue and black group by us, also includes distinct bands of green in Williams (2022). However, removing that ship from the statistical analyses made no difference to the outcomes.

In sum, with a liberal approach to the formal statistics, we looked for differences across observers, skyscapes, ship classes, and camouflage colours by performing 42 two-tailed *t*-tests and found no significant effects, even without Bonferroni correction. This does not mean there are no effects for these factors in general, but there is no evidence for mean differences in Blodgett's cleaned data under the constraints of his reporting and his experimental design.

#### Part 3: Detailed Analysis: Cluster Statistics and Hysteresis and Twist Effects

Contemporary work on dazzle camouflage measured perceived directions as functions of true (physical) directions (Lovell et al., 2024). Blodgett's (1919) approach was not so systematic, but as a first step in aligning his study with Lovell et al.'s, we next concentrated on the central tendencies of the data clusters. The two black diamonds in Figure 7(a) are placed at average actual target angles (*x*-axis, *α*; strictly, angle on the bow) of 53.9° and 127.0° [equivalently, −36.1 and 37.0° relative to horizontal (90°)]. The average perceptual errors (signed deviations from veridicality) denoted by these diamonds are 31.4° (SD = 26.5°) and −18.0° (SD = 28.35°) for the left-hand and right-hand clusters, respectively. Averaging magnitude across clusters gives 24.7°.^7^ These perceptual errors are markedly less than the value of 39.2° that we derived for the pre-cleaned data (Figure 6(a)).

We performed statistical analyses on the perceptual error distributions (Figure 7(a)) for each of the three/six observers. We started by confirming that the means of each of the two cluster distributions were significantly different from zero for all three observers (Table 3, rows 1–3), indicating a systematic deviation from veridicality for Blodgett's observers (i.e., that they were biased). Second, we investigated whether observer means were statistically different from a hypothetical (and natural) default setting of horizontal (90°). The average of the lefthand cluster differed from horizontal by only −2.88°, and this was not significant for any of the observers (Table 3, rows 4–6, lefthand cluster). The standard deviation of this distribution (26.5°) was far too large to claim that participants were simply attempting to set the response dial to horizontal on each trial, but the central tendency was not significantly different from this. However, the average observer differed from horizontal by 18.5° in the right-hand cluster, and the difference was significant for each of the three/six observers (Table 3, rows 4–6, right-hand cluster). This is good evidence that the behavioural responses included a perceptual component (i.e., that the bias identified above was not pure response bias to horizontal). The question then, what was the basis for the perceptual bias?

**Table 3.** Statistical analysis (*t*-tests) for the clusters of data in Figure 7(a). The first six rows are for two-tailed single sample *t*-tests. The bottom three rows are for one-tailed unmatched two-sample *t*-tests. PE = signed perceptual error; PTA = perceived target angle; MPE = magnitude of perceptual error; LHC, RHC = lefthand and righthand clusters; ** = statistically highly significant.

Row No.	Comparison	Observer	Lefthand cluster (Figure 7(a))		Righthand cluster (Figure 7(a))	
			*t*(DF)	*p*	*t*(DF)	*p*
1	PE vs. 0°	1	7.53 (49)	<.001**	−3.03 (33)	<.005**
2	"	2	12.03 (41)	<.001**	−7.25 (43)	<.001**
3	"	3–6	5.48 (36)	<.001**	−3.81 (40)	<.001**
4	PTA vs. 90°	1	−0.62 (49)	.539	3.99 (33)	<.001**
5	"	2	−0.09 (41)	.924	6.77 (43)	<.001**
6	"	3–6	−1.04 (36)	.303	3.36 (40)	.002**
7	MPE: LHC vs. RHC	1	2.46 (82)	.008**	–	–
8	"	2	4.34 (84)	<.001**	–	–
9	"	3–6	1.36 (76)	.089	–	–

**Figure 8.**
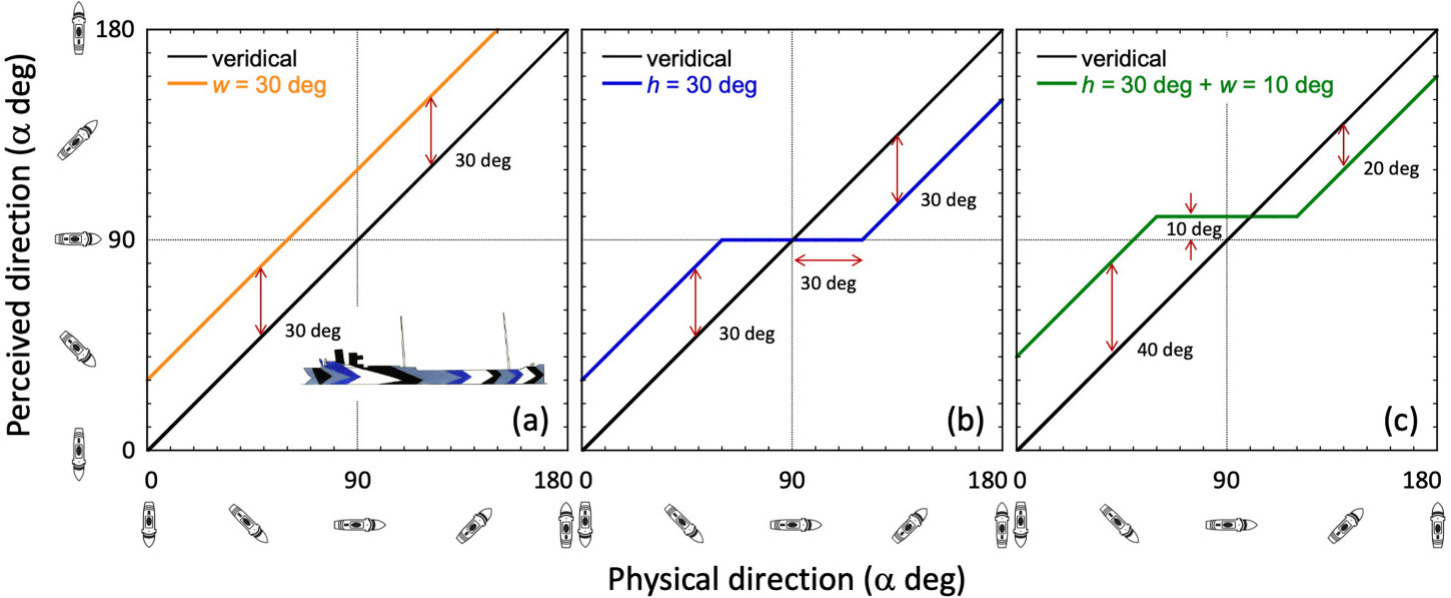
Schematised misperceptions of ship directions for target angles between 0° and 180°. The ship icons denote the direction of the target relative to the observer. (a) A twist (*w*) effect of 30 deg. Here, the bow of the ship twists away from the observer for all physical directions. (b) A hysteresis (*h*) effect of 30 deg. Here the ship is drawn to horizontal (90°) for a range of ±30° and remains offset from veridicality by that same amount thereafter. (c) The combination of hysteresis and twist. In this example, *h* and *w* are different in size: 30° and 10°, respectively. The function in (c) has exactly the same form as the one in (b) but is offset vertically by 10° (owing to twist). Note that if this were the real situation, an experimenter ignorant of hysteresis and averaging unsigned errors across the left and right parts of the function (e.g., Blodgett, 1919), would find an effect size of 30° (in this example), having nothing to do with twist. Adapted from Lovell et al. (2024).

By way of introducing our answer, Figure 8(a) illustrates what Lovell et al. (2024) referred to as a twist effect (*w*) (Blodgett (1919) and Bates (1918) used similar language). In this illustration, the forced perspective from the dazzle camouflage (illustrated by the inset of Blodgett's Design 2; Figure 3) twists the perceived direction of the ship away from the observer, in this toy example, by 30°. This is the type of effect that Blodgett (and others) appear to have been expecting to find. However, Lovell et al. (2024) found a second effect in their experiment that they referred to as hysteresis, or horizon bias (*h*). This is a tendency for the perceived direction of the ship to be drawn to parallel the horizon (a perceived target angle of 90°) for some range of directions, in this example (Figure 8(b)), ±30° (we say, *h *= 30°). For directions beyond this, some of the pull from the horizon is overcome, but the effect maintains its influence causing the perception of direction to be offset from veridical by *h* deg. This has no systematic relation to the application of dazzle camouflage since Lovell et al. found it in all their conditions including neutral grey. Note that for twist, the perceptual effect is always in the same relative direction: away from the observer for texture gradients towards the bow, as in this example and relevant WWI designs (Bement, 1919b; Blodgett, 1919; DuBose, 1937; Lovell et al., 2024; Williams, 2001). For hysteresis, on the other hand, the effect pulls the bow towards the observer for physical directions away from the observer, and away from the observer, for physical directions towards the observer. This means that if both effects are in play, they will combine constructively over half of the plot and destructively over the other half. This is exactly what Lovell et al. found in their experiment, though the magnitude of twist was much less than that of hysteresis (about a quarter to a third) as illustrated in Figure 8(c).

**Figure 10.**
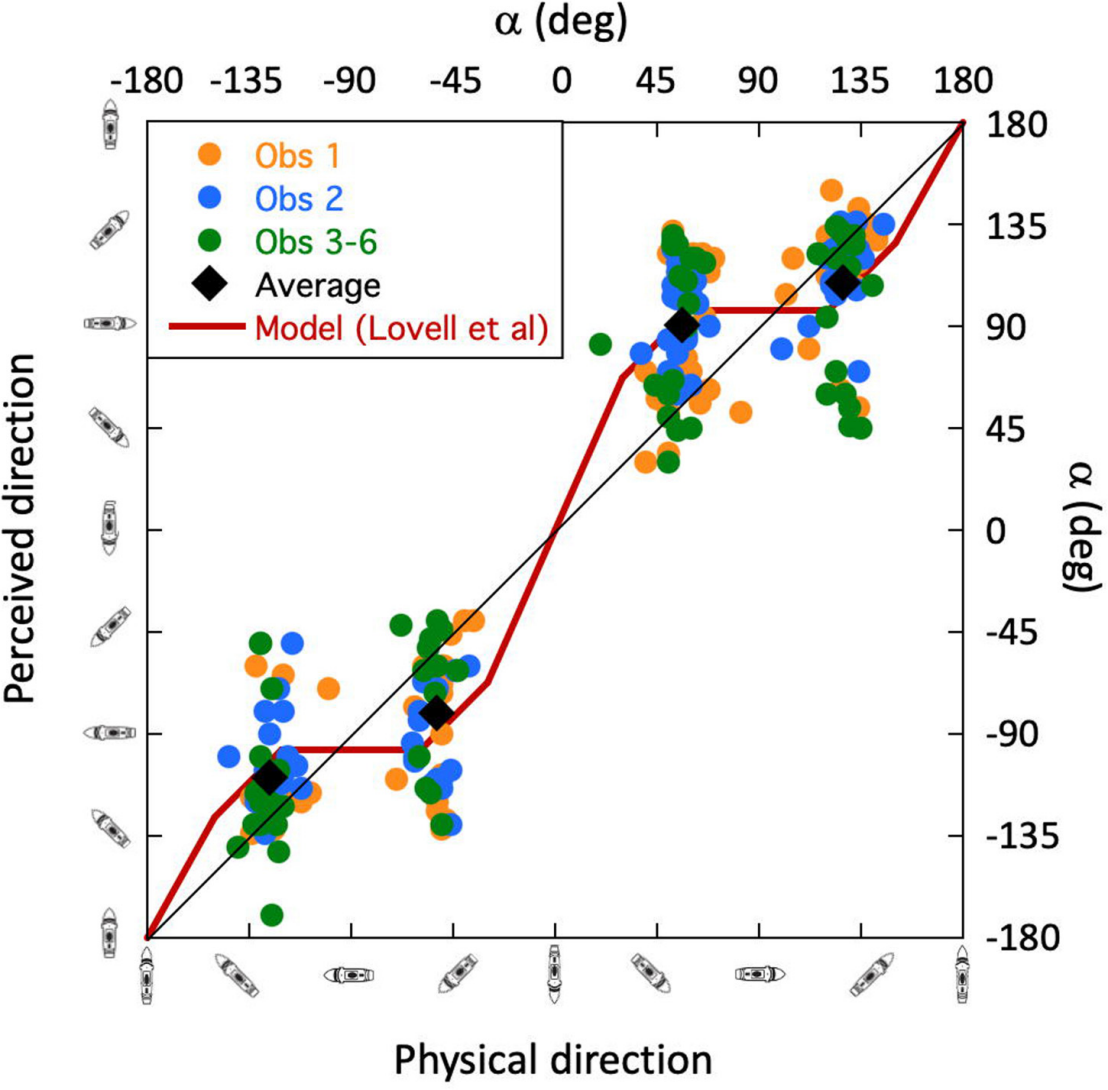
Results from Blodgett (1919) (excluding Design 6) for target angles from −180° to 180°, and model curve (red) derived from the four relevant dazzle conditions in Lovell et al. (2024) (*h *= 30.6, *w *= 7.0, *d *= 29.8°) (The *d* parameter determines the distances from −180°, 0° and 180° that the model curve begins its cuts back to veridicality but is of little interest here; see Lovell et al. (2024) for further details). The diagonal black line is the contour of veridicality, and the black diamonds are cluster averages. The model curve slightly overestimates the effect size of Blodgett's averages (black diamonds) largely because the hysteresis effect (*h*) was 6.2° larger for Lovell et al. than for Blodgett.

Towards the two ends of this general perceptual function, Lovell et al. supposed that the perceptual effects would diminish, returning the effect curve to veridicality at 0° and 180° (for ships with the same camouflage on each side). They modelled this with a third parameter they called decay (*d*). This is of little relevance here (and is not shown in Figure 8), but features incidentally in our model plot, looking ahead to Figure 10.

Note that in Figure 8(c), the combined effects of twist and hysteresis deviate from veridicality with an asymmetry around the physical 90° mark (horizontal/East). This is different from the hallmarks of pure twist and pure hysteresis, where the deviations are of the same magnitude in either the same direction (Figure 8(a)) or in opposite directions (Figure 8(b)) either side of a physical target angle of 90°. Casual inspection of the results in Figure 7 rule out a pure twist effect (Figure 8(a)) beyond reasonable doubt, but we turned to inferential statistics to decide between the arrangements in Figure 8(b) and (c) by testing whether the magnitudes of the perceptual errors in the two clusters (overall means of 31.4° and 18.0°) were significantly different. This confirmed that the perceptual errors were larger for the lefthand cluster than the righthand cluster for both Observer 1 and Observer 2 (Table 3, rows 7 and 8), providing good evidence for a combination of both hysteresis and twist for the two experts. For Observers 3‒6, the effect was in the same direction as for the experts but did not reach significance (Table 3, row 9). Thus, while the naive group might have experienced both hysteresis and twist (their data are consistent with this), we cannot rule out the possibility that their mean effects owed purely to hysteresis.

Finally, we estimated the size of the two effects (*h* & *w*) for Blodgett's results (excluding his neutral grey and black control conditions for which he did not provide the relevant data). In Lovell et al. (2024), these parameters were estimated by finely sampling the physical direction dimension and fitting model curves to their results. The data provided by Blodgett (1919) do not lend themselves to this, but are sufficient for us to estimate the magnitudes of the two effects, nonetheless. This is readily achieved by realising (from the geometry in Figure 8(c)) that *h* is estimated by averaging the magnitudes of mean perceptual errors in the righthand and lefthand clusters in Figure 7(a), whereas *w* is half the difference between them. In other words, referring to the lefthand and righthand mean magnitudes of perceptual error as *λ* and *ρ*, we have, *h *= (*λ* + *ρ*)/2, and *w *= (*λ* − *ρ*)/2. Note that *h* is always positive, whereas *w* can be positive or negative^8^ because, in principle, the twist effect could systematically pull the bow towards the observer instead of pushing it away (if the direction of the camouflage gradient were reversed, for example).

**Figure 9.**
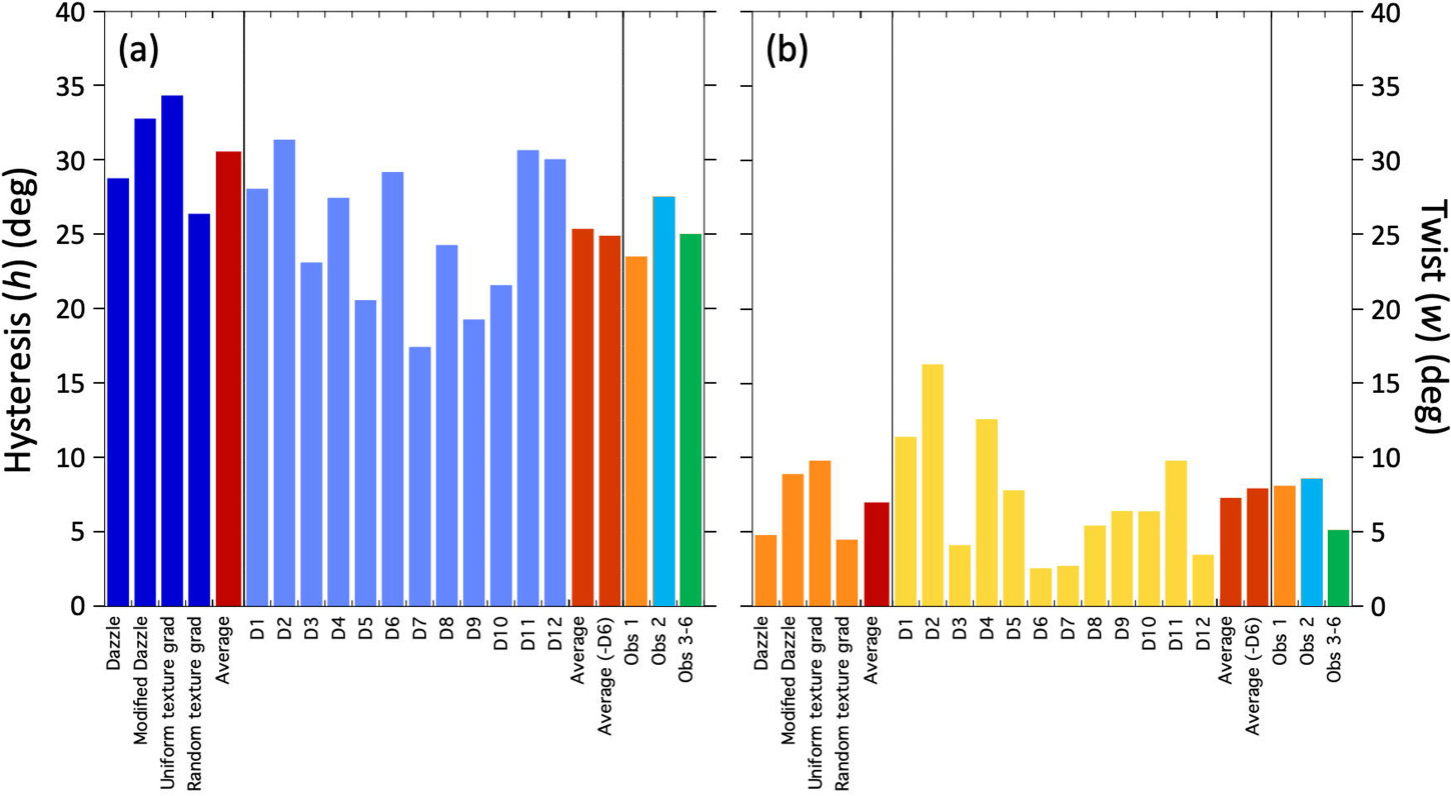
Estimates of hysteresis (a) and twist (b) for the studies by Lovell et al. (2024) (plotted to the left of each panel) and Blodgett (1919). The averages for Lovell et al. are for their four relevant dazzle conditions. (From left to right, Lovell et al. referred to these as: Mauritania dazzle, Simplified dazzle, Regular circles and Irregular circles.) The particoloured design from Lovell et al. was excluded here since it contained no obvious texture gradient and produced no positive twist (on the target angle convention). For Blodgett, the averages are for (i) the full set of 12 dazzle designs (D1–D12, to their left) and (ii) the same set but with Design 6 removed. Estimates are also shown for the three/six observers from Blodgett (1919) on the right. The standard errors (*SEs*) on the estimates for the individual designs, their average, their average -D6, and each of the three observers (treating Observer 3–6 as a single observer) for both *h* and *w*, were 11.9 (the average SE for D1–D12), 3.5°, 3.6°, 6.6°, 4.0°, and 7.6°, respectively. From Lovell et al. (2024), the *SE*s for the four designs and the average of those for *h* were 2.9°, 3.2°, 2.3°, 2.2°, and 2.6°, and for *w* were 0.9°, 1.3°, 1.1°, 0.9°, and 1.0°.

The results of this analysis (see S4 for further arithmetic details) are shown in Figure 9(a) and (b) for hysteresis and twist, respectively (see figure caption for details of standard errors). We did this for each of the individual dazzle designs (D1 to D12) (see also S3), for the three/six observers, the overall average, and an average with Design 6 removed (“Average (-D6)” in the plots). We included this last arrangement because, a priori, Design 6 did not look to us like a good example of a texture gradient towards the bow. (Perhaps this is what Blodgett meant by “reverse perspective” and is the implication of his proposal under *Stimulus design* in S1). Removing D6 from the analysis was intended to improve our overall estimate of Blodgett's twist (though it made little difference in practice). The result was *w *= 7.2°, which compares very well with *w *= 7.0° averaged across the four twisting dazzle conditions investigated by Lovell et al. (2024). Similarly, the hysteresis values of *h *= 24.4° for Blodgett and *h *= 30.6° for Lovell et al. (based on the same conditions as for twist) are in broad agreement. Note that the estimates of hysteresis and twist varied quite markedly across Blodgett's 12 designs, but the variance for these data was also quite large (the average cluster SD for individual designs was 26.9°; see S3 for plots) and the sample size for each design was quite small (the average number of cleaned observations for each design was 20.7 spread across the two clusters; see S3). Therefore, we advise treating the specifics of this observation with caution (see the caption of Figure 9 for standard errors). Nonetheless, it is remarkable that *w* was positive for all 12 designs. If we were looking at a random variable, then we would expect to see both positive and negative values; the probability of all 12 estimates of *w* being positive by chance is 1 in 4,096. Therefore, we are confident that perceptual twist was a factor in Blodgett's work, but much less than he probably supposed, assuming he attributed his average effect size (Figure 6(a)) to *w*. Blodgett did not in fact report an overall effect size, but in any case, the average magnitude of the experimental effect relates to *h* and has nothing to do with *w*, which Blodgett certainly did not estimate. (See the caption to Figure 8 for further details.) From Figure 6(a), our estimate of the supposed overestimation of twist by Blodgett is 32° (39.2–7.2°). What is also clear is that in both studies the magnitude of hysteresis was around three or four times greater than for twist. (The probability of all 12 measures of hysteresis and twist here being different in the same direction is 1 in 2,048).

We made our final comparison across studies in Figure 10 which shows model results (involving *h*, *w*, and *d* parameters) from Lovell et al. (based on their four relevant dazzle conditions) against the cleaned data set from Blodgett (excluding Design 6) but now unfolded across the mirror reversal to show the full range of target angles from −180° to 180°. Note how the model curve (red) correctly captures (without fitting) the asymmetries of the perceptual errors on both sides of a physical target angle (*α*) of 0°. This owes to the non-zero value of twist (Figure 8(c)).

### General Discussion

Details aside, the most striking characteristics uncovered by our analyses above are (i) how similar the results are across Blodgett (1919) and Lovell et al. (2024) and (ii) how much greater the hysteresis (*h*) effect is compared with twist (*w*). The analysis here extends the findings of Lovell et al. (2024) to a greater range of ship classes, colours (stimuli from Lovell et al. stimuli were all grey scale), seascapes, skyscapes, camouflage designs (according to Van Buskirk (1919b) there were 495 approved American designs), experimental setups, and an expert naval observer. We note, however, that while the overall measure of systematic perceptual bias known as twist was remarkably similar across the two studies, this detail is perhaps serendipitous because twist is known to vary across designs (Lovell et al., 2024), presumably as the professional camoufleurs would expect (e.g., DuBose, 1937). However, there is a critical difference in the results across studies. In Lovell et al., the dazzle results were consistent with what they found for a neutral grey condition where hysteresis was preserved (in fact, slightly greater than for the dazzle conditions) but twist was much diminished. In contrast, Blodgett found that experimental effects were all but absent for his neutral grey and black ship conditions (Figure 6(a)). This was crucial for Blodgett (1919) since it indicated that the application of dazzle had an enormous effect (regardless of the lack of detail in his analysis), whereas for Lovell et al. (2024) the impact was rather modest. To address this inconsistency, we conducted our own control experiment using photographs of a selection of dazzle ships from Blodgett's thesis and those same ships edited in photoshop to replace the dazzle camouflage with neutral greys.

## A New Control Experiment

### Methods

**Figure 11.**
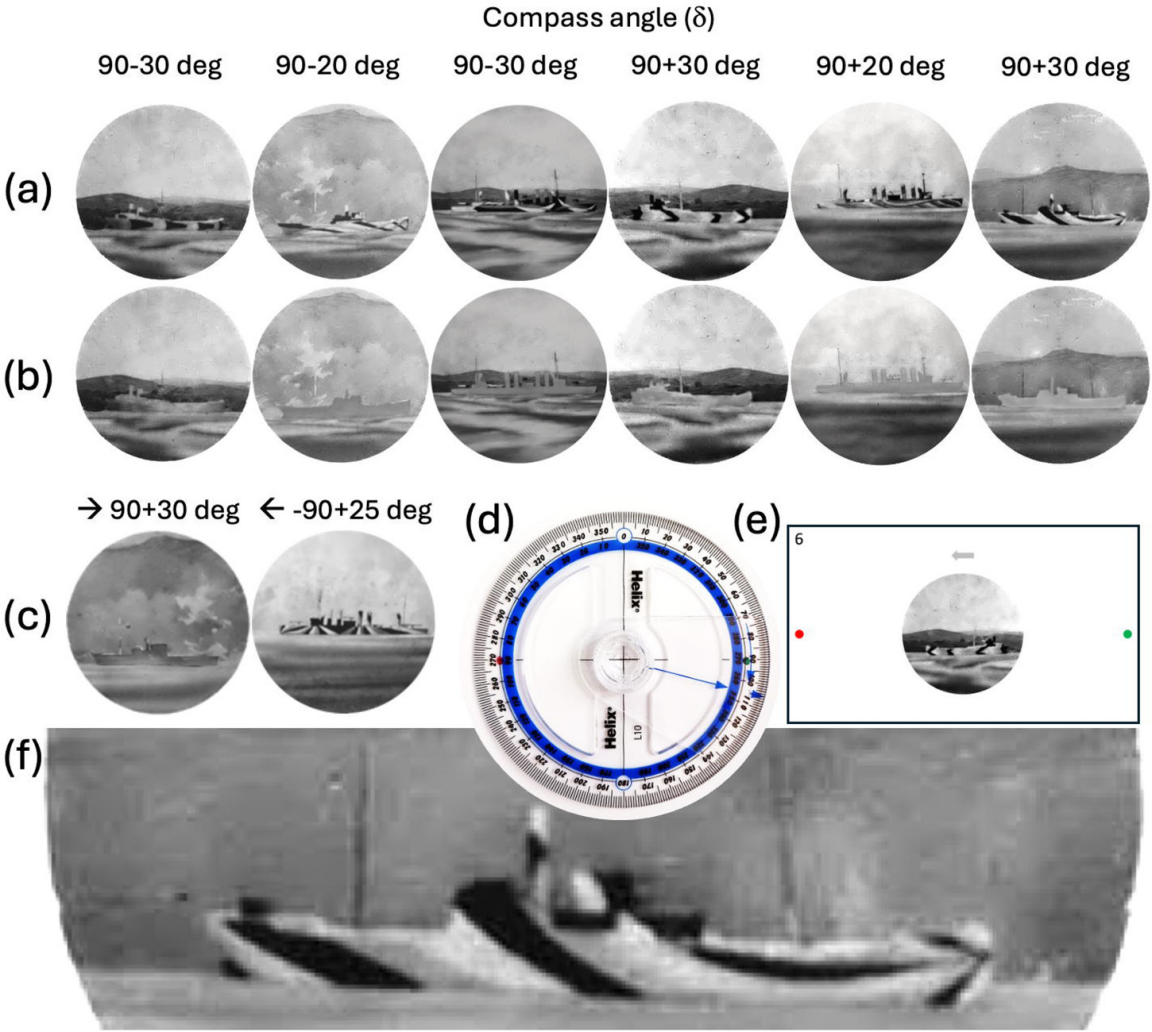
Example stimuli and methods for the new control experiment. (a) Original photographs, subject to re-windowing and mirror reversal as appropriate (all of these point East), and their compass angles as reported by Blodgett (1919). (b) The same as in (a) but with the dazzle camouflage edited to a neutral grey (camouflage control). Where there was clear evidence for a shoreline or horizon, the orientation of some of the photographs was adjusted to achieve horizontal alignment. Re-windowing was performed on the destroyer images which were ellipses originally (S1 and S2). In these cases, Adobe Photoshop's Generative Fill, AI tool was used to fill in missing background as appropriate. All these adjustments were identical for grey and dazzle ships (i.e., the grey transform was performed last). Owing to a preference to restrict image editing to as little as plausible, no attempt was made to equate the vertical position of the shoreline/horizon or to centre the ships. (c) The two stimuli used for the two practice trials. The grey ship is heading generally East, but 30° towards the observer. The dazzle ship is heading generally West but 25° away from the observer. (d) The protractor used by the participants to make their responses. Note that responses were made according to compass angle, but we present the results (Figure 12) by target angle for consistency with earlier figures. (e) Example display screen for a single trial. Note that the number in the top left corner of the display was used by the experimenter when recording responses but had no systematic relationship with the stimulus condition. The participant adjusted the compass in (d) to match the perceived direction of the ship in (e). In this example, the protractor would need to be adjusted through around 140° in the westward direction to make a reasonable match. (f) An enlarged version of the ship on the far right in (a).

Our aim was to use ship images with target angles counterbalanced around ±90°. The six images we selected from the black and white photographs of Blodgett (1919) are shown in Figure 11 in both their original camouflage (Figure 11(a)) and our edited versions to grey (Figure 11(b)). (Note that for ease of comparison with the protractor (Figure 11(d)), which participants used for making their responses (see below), we report compass angle rather than target angle for the ships in Figure 11.) This is not the experimental design we would have produced from scratch since that would distribute target angles across constant ship designs and camouflages (cf. Lovell et al., 2024), but our choice of images was severely limited (Blodgett, 1919; S1 and S2). Note that by switching ship and camouflage designs across target angle (as we did) there was a potential confound for our estimates of hysteresis (*h*) and twist (*w*) because these require estimates of perceptual errors for comparable conditions each side of horizontal (90°) (see Figure 8). Nonetheless, this did not impact our primary aim of comparing dazzle ships with the same ships in neutral grey camouflage since this factor was balanced across target angle. Note also that our stimuli lack the original colours used by Blodgett (Figure 3). However, because (i) our analysis found no colour effects (Figure 7(d)), (ii) Bittinger and Hulburt (1936a) advocated that the effectiveness of dazzle was not enhanced by colour, and (iii) DuBose (1937) came to propose using three shades of grey in dazzle ship painting, we do not consider the achromaticity of our stimuli to be a problem. On the other hand, Blodgett implied that (some or all of) his photographs were taken with colour filters, perhaps intending to diminish the dazzle effects for (curious) purposes of illustration (see S1, p. 12). Even so, while this might interfere with our attempts to measure twist, it does not undermine our primary aim. Finally, Blodgett pointed out that his photographs were taken from a slightly higher vantage point than that through the periscope in his experiment, claiming that this made it easier to judge direction in the photographs than in his experiment. But again, while this might interfere with effect size, there is no reason to suppose that it would undermine our primary aim.

We also created mirror reversed versions of each ship image (not shown) producing a total of 24 test stimuli and included two further examples (Figure 11(c)) for two practice trials. The ship stimuli were presented on a laptop screen at a comfortable viewing distance. (At a conventional arm's length distance of 57 cm, this produced images (Figure 11(e)) about 12° in diameter, but viewing distance was not strictly controlled). Participants were given unlimited viewing time to indicate their perceived directions of travel using a 360° protractor (Figure 11(d)) and the settings were recorded by the experimenter. The protractor was marked with green and red dots to provide co-registration with the display screen that also contained these dots (Figure 11(d) and (e)). The ship stimuli were presented in a different random order of 24 experimental trials (one trial per stimulus) for each participant and the general direction of travel (West or East) was shown by an arrow at the top of the display (Figure 11(e)) to ensure that the bow was identified correctly. Participant instructions were presented over two sequential display screens at the beginning of the experiment and are included in Supplementary Material 5 (S5). Participants (*n *= 16) moved forward through the experimental sequence by clicking the trackpad of the laptop. No feedback was provided to participants on the true directions of the test ships. Participants wore their normal optical correction and were recruited on a voluntary basis by the two authors. The work was done with informed consent and under ethical approval by the College of Health and Life sciences at Aston University (#856).

### Results and Discussion

**Figure 12.**
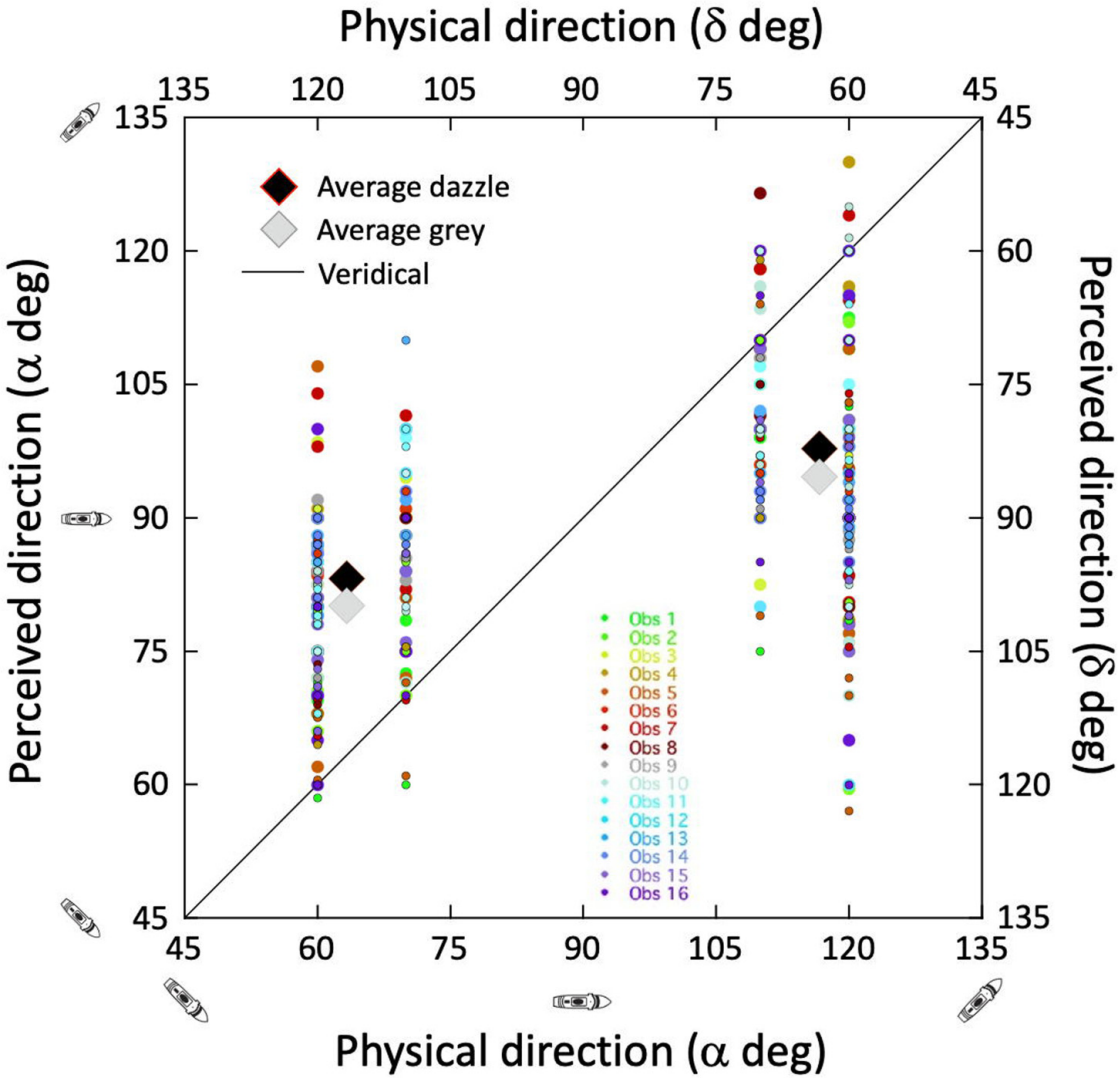
Results for the new control experiment using edited images from photographs of Blodgett's stimuli. The small, coloured points are for our 16 naive observers (the points for the dazzle condition are slightly larger than for the grey condition which also have black surrounds). The large diamonds are the group averages for the dazzle (black) and grey (grey) conditions for each of the two clusters by comparison with Figure 7. The standard deviation of perceptual errors across the full data set was 23.8° and 23.0°, for the dazzle and grey conditions, respectively. The average observer SD within condition was 10.35°.

The results were collapsed over mirror reversals (c.f. Figure 6) for each observer and plotted in Figure 12. The black and grey diamonds show means of the cluster distributions for the dazzle and grey camouflage conditions, respectively. For the dazzle conditions (Figure 6(a)), these equate to signed perceptual errors of 19.8 and −18.9° for the left and right clusters. For the grey control conditions the perceptual errors were 16.7 and −22.1°. Statistical analysis confirmed that these errors were greater than zero for both camouflage conditions (dazzle and grey) for each cluster of results (lines 1 and 2 in Table 4) providing evidence for bias in our image processed versions of Blodgett's stimuli. Further analysis confirmed that the response settings were significantly different from 90° in all cases (lines 3 and 4 in Table 4) providing evidence for the detection of signal (correct signed deviation of perceived direction from 90°).

**Table 4.** Statistical analysis for the new control experiment. All rows are for two-tailed single sample *t*-tests. PE = signed perceptual error; PTA = perceived target angle. ** = statistically highly significant.

Row No.	Comparison	Condition	Lefthand cluster (Figure 12)		Righthand cluster (Figure 12)	
			*t*(DF)	*p*	*t*(DF)	*p*
1	PE vs. 0°	Dazzle	14.7 (15)	<.001**	−17.1 (15)	<.001**
2	"	Grey	11.0 (15)	<.001**	−15.6 (15)	<.001**
3	PTA vs. 90°	Dazzle	−5.06 (15)	<.001**	7.04 (15)	<.001**
4	"	Grey	−6.50 (15)	<.001**	3.25 (15)	.005**

We followed our earlier procedure to estimate hysteresis (the pull of the diamonds in Figure 12 away from veridicality and towards 90°) and twist, finding *h *= 19.4 and *w *= 0.47° for the dazzle condition and *h *= 19.4 and *w *= −2.65° for the neutral grey condition (the average standard error for these four measures was 0.95°). For both camouflage conditions, *h *> *w* [dazzle: *t*(15) = 14.7, *p *< .001; grey: *t*(15) = 11.0, *p *< .001; two-tailed]. The values of hysteresis were not significantly different across conditions [*t*(15) = 0.069, *p *= .95; two-tailed], but the values of twist were [*t*(15) = 2.47, *p *= .026; two-tailed].

The hysteresis results here support our contention that Blodgett's control results for his grey (and presumably also his black) painted ship conditions (Figure 4(a)) are unsafe, our own control comparison being much more like that in Lovell et al. (2024) but using image-processed versions of Blodget's own ships. The large values of hysteresis in this experiment are similar across the two camouflage conditions (grey and dazzle), and broadly comparable with those derived from the other two studies (about 5° less than our estimate from Blodgett's results). On the other hand, our estimates of twist are notably diminished here. In fact, for our neutral grey condition, twist was negative (using the target angle convention), indicating an overall bias for the bow to pull towards the observer. We suspect this has more to do with the particular choice of stimuli than neutral camouflage in general, not least because Lovell et al. found a small effect of twist in the opposite direction to this for their grey ship stimulus. (Readers making comparisons across these studies are reminded of footnote 8.) We also note that the application of dazzle camouflage here twisted the bow away from that found for the grey ships by an average of 3.1° (the upward shift of the black diamonds relative to the grey diamonds in Figure 12). This is consistent with the direction of the texture gradient and the conventional direction for the twist effect. Overall, despite our misgivings about the stimuli (see previous section) and the diminished effect sizes we expected (and found) because of this, our results here confirm that (i) the hysteresis effect is much larger than the twist effect, (ii) it is present regardless of the application of dazzle paint, and (iii) the application of a dazzle texture gradient induces a small amount of perceptual twist.

## Overall Discussion

### Blodgett's Direction Convention

As mentioned earlier, we assumed that Blodgett's ship angles (actual and perceived) were reported under the compass convention, but there are two other cogent possibilities. First, he might have used a mathematical convention where zero referred to East, and angles were measured counterclockwise relative to that (e.g., 90° = North). However, this is unlikely because it would imply that in his experiment perceived direction was drawn to the North/South axis. This would be an incomprehensible form of ‘hysteresis’ orthogonal to the horizon, with perceived directions gravitating towards ‘directly away’ or ‘directly toward’ the observer. The second possibility is that Blodgett used the target angle convention. In this case, his results would be consistent with a perceptual draw to the horizon (i.e., Lovell et al.'s (2024) report of hysteresis), but in the plots here, this data transform would deliver negative twist, the forced perspective pulling the bow towards the observer, contradicting the direction of the texture gradient. In sum, Blodgett's results are consistent with our plausible assumption about Blodgett's meaning of angle (see the What Does all This Mean for Blodgett? section) and the results of Lovell et al. (2024) and would be utterly mystifying under the two other plausible interpretations.

### Hysteresis, Twist, and Expertise

The undisputed aim of dazzle camouflage was to interfere with the aiming capabilities of enemy submariners. As noted in the report by Lieutenant Van Buskirk (1919a, p. 305) to the US Navy Department's Bureau of Construction and Repair, “It must be remembered that the sole object of dazzle painting is to cause confusion as to the course and speed of a vessel …”. This included using false perspective designs to systematically twist the perceived direction of the ship from its true course. However, one of the main outcomes from Lovell et al. (2024) (and corroborated here; Figure 10) was that while twist alone would obviously benefit the target ship (Figure 8(a)), other than for the fastest ships (and largest, e.g., troop carrying; see Lovell et al. (2024)), its interaction with hysteresis would effectively abolish this benefit once the full range of target angles are considered. This is because although (positive) twist adds to the benefit of hysteresis for target angles towards the observer (|*α*| < 90°), it subtracts from that benefit for target angles away from the observer (|*α*| > 90°) (Figure 8(b) and (c)). While twist appears to have been one of the objectives for dazzle camoufleurs (including Blodgett (1919) and Lovell et al. (2024)) towards the end of the war (Behrens, 2016; Bement, 1919b; Bittinger & Hulburt, 1935, 1936a; DuBose, 1937; Warner, 1919b), we have found no mention of the hysteresis effect (observed here in Blodgett's data and our control experiment and by Lovell et al. (2024)) in the literature from that time, never mind its interaction with twist. This is most surprising since it is difficult to imagine that seafarers would not have witnessed this effect first hand, and we welcome any forthcoming information on the matter.

The hysteresis effect occurs because the pictorial depth information (3D relief) inherent in the perspective (polar) projection of a 3D object of fixed size diminishes with distance from the observer because the visual projection asymptotes towards parallel (this underlies the misnomer known as lens compression in telephoto lens photography). In fact, the silhouette of a ship in parallel projection for a target angle of 90 + *x* deg is identical to one with a target angle of 90 − *x* deg. As a distant object rotates from horizontal (90°) towards or away from the observer, the projective transform of the object is one of foreshortening. The only other relevant information is the occlusion/compression or disocclusion/stretching of rear or front facing image data (e.g., the bridge windows coming into view) including lighting and shadows, not available in a silhouette, and cognitively weak for strongly elongated stimuli such as ships (e.g., see Figure 5 in Lovell et al., 2024). Excepting the extremes, the foreshortening of object length (Bittinger & Hurlburt, 1936a) is a useful cue for a deviation of target angle from horizontal (90°) only if either (i) the rotation of direction is being witnessed dynamically (which was not the case for Blodgett or Lovell et al.), or (ii) the observer has good knowledge of the target's true length either from memory or from identification books (Florek, 2017a). Blodgett went to some lengths to facilitate his two experts’ memories of this information, but the similarity of their results against the naive observers suggests this was of little or no benefit under Blodgett's experimental conditions. In the absence of any (useful) information to the contrary, it is natural for visual perception (and cognition) to default to an average direction of horizontal (±90°) since this minimises the expected errors of judgement. This is the hysteresis effect, and deviations from this (e.g., at target angles of 60° and 120° in Figure 8(b)) indicate the availability of visual information that allow observers to overcome its resistance, at least in part. (The relevant visual information would include details of front and rear surfaces of the superstructure and any residual information from perspective projection at that distance.) By contrast to experimental participants, wartime submariners had access to identification books with silhouettes of ship classes (see Florek, 2017a; Hardy, 1943; Jane, 1914; ONI, 1944) to establish the true length and height of a ship class once identified. Presumably, these would diminish the effects of both hysteresis and twist. Nonetheless, our analyses of Blodgett's data accord with the findings of Lovell et al. (2024), showing that WWI-style dazzle painting provided modest protection at best in biassing the perception of a ship's direction of travel.

### Response Variance, Uncertainty/Confusion, Weather, and Experience

There are two components to any psychophysical measure of perception: bias and variability. Hysteresis and twist are both functions of perceptual bias (Figure 8), and (eventually) camoufleurs aimed to capitalise on one of these, twist, in their designs. But a second component to dazzle design involves perceptual variability through uncertainty, or confusion. In general, and in the absence of systematic perceptual bias, perceptual variability (around veridicality) would also benefit ships (DuBose, 1937, p. 19) on those occasions where the enemy's estimation of course deviated substantially from the mean/veridical mark one way or the other. However, neither Blodgett (1919) nor Lovell et al. (2024) set out to investigate this factor. The large data spreads in Blodgett's results (see Figure 7; SD = 27.2°) contrast sharply with the fine measures of angle discrimination for simulated planar surfaces using pictorial depth cues (e.g., Meese & Holmes, 2004), suggesting high perceptual variability. However, Blodgett's experimental design makes it difficult to draw conclusions because we do not know whether these data characteristics are due to dazzle features alone or involve uncontrolled factors such as seascape and simulated weather (see the Methods section).

One might expect experience to influence the effectiveness of dazzle, but our analysis of Blodgett’s (1919) averages revealed no obvious superiority for either one of his experienced observers over each other (they had different expertise; see Methods section) or the naive group. In contrast, in a much more carefully controlled study, Lovell et al. (2024) found that hysteresis decreased with expertise. The details of how response strategies might have differed across Lovell et al.'s participants is not clear, but the general issue is worthy of further investigation because experienced submariners would have had the benefit of identification books not available to participants in either of the two experimental studies.

## Overall Conclusion and a Eulogy for Blodgett

According to Wilkinson (1922), 4,000 merchant ships and over 400 war vessels received the dazzle treatment in WWI. This cost £2.5 M in 1918, equivalent to over £225 M in 2024. While only a small fraction of the total costs of WWI to Great Britain and the US (estimated at $58B in 1914–18 US dollars (“World War I: The financial cost of the war”, n.d.)) one would want an effective return on these monies nonetheless, and one senses the frustrations of those trying to source quantitative data on the matter (Bittinger & Hulburt, 1936a; Blodgett, 1919).

Blodgett (1919) is to be applauded for taking on the quantitative task of investigating dazzle, and although we have identified numerous shortcomings with his experimental design and analysis, it should be kept in mind that while experimental psychology has its origins in the late 19th century, today's guiding principles of investigative design and statistics (e.g., Fisher, 1925a, 1925b, 1935) were not mainstream at the time of Blodgett's thesis. Blodgett appears to have been overwhelmed by the number of potential experimental factors he encountered at the test facility (see S1, p. 9; S2, pp. 7, 11). And by trying to embrace them all, he sacrificed systematic manipulation, perhaps for the seductive draw of more dazzle designs than was strictly necessary for an initial foray into the science.

In spite of the shortcomings in the research above, the resources would have seemed considerable for those observing proceedings close up (manpower, test facilities, and finances) and Blodgett must have been warmly gratified to find what seemed like strong quantitative support for the theoretically sound (Warner 1919a, 1919b) and intuitively appealing concept of dazzle twist (Figure 1(d) and (e)), particularly under the purview of associated authorities on the matter. It is perhaps not surprising then that Blodgett's undergraduate results for non-dazzle ships, seemingly an afterthought and from an unidentified observer, did not rock the boat.

We can only share Blodgett's frustrations about the absence of quantitative science in studying dazzle camouflage, but hope that the analyses here and in Lovell et al. (2024) have begun to redress the balance, albeit over a century late. Much work remains to be done.

## Supplemental Material

sj-docx-1-ipe-10.1177_20416695241312316 - Supplemental material for Blodgett's (1919) “Ship camouflage” 105 years on: A misperception of dazzle perception revealed and redressed Supplemental material, sj-docx-1-ipe-10.1177_20416695241312316 for Blodgett's (1919) “Ship camouflage” 105 years on: A misperception of dazzle perception revealed and redressed by Timothy Simon Meese and Samantha Louise Strong in i-Perception

sj-docx-2-ipe-10.1177_20416695241312316 - Supplemental material for Blodgett's (1919) “Ship camouflage” 105 years on: A misperception of dazzle perception revealed and redressed Supplemental material, sj-docx-2-ipe-10.1177_20416695241312316 for Blodgett's (1919) “Ship camouflage” 105 years on: A misperception of dazzle perception revealed and redressed by Timothy Simon Meese and Samantha Louise Strong in i-Perception

sj-docx-3-ipe-10.1177_20416695241312316 - Supplemental material for Blodgett's (1919) “Ship camouflage” 105 years on: A misperception of dazzle perception revealed and redressed Supplemental material, sj-docx-3-ipe-10.1177_20416695241312316 for Blodgett's (1919) “Ship camouflage” 105 years on: A misperception of dazzle perception revealed and redressed by Timothy Simon Meese and Samantha Louise Strong in i-Perception

sj-docx-4-ipe-10.1177_20416695241312316 - Supplemental material for Blodgett's (1919) “Ship camouflage” 105 years on: A misperception of dazzle perception revealed and redressed Supplemental material, sj-docx-4-ipe-10.1177_20416695241312316 for Blodgett's (1919) “Ship camouflage” 105 years on: A misperception of dazzle perception revealed and redressed by Timothy Simon Meese and Samantha Louise Strong in i-Perception

sj-docx-5-ipe-10.1177_20416695241312316 - Supplemental material for Blodgett's (1919) “Ship camouflage” 105 years on: A misperception of dazzle perception revealed and redressed Supplemental material, sj-docx-5-ipe-10.1177_20416695241312316 for Blodgett's (1919) “Ship camouflage” 105 years on: A misperception of dazzle perception revealed and redressed by Timothy Simon Meese and Samantha Louise Strong in i-Perception

sj-docx-6-ipe-10.1177_20416695241312316 - Supplemental material for Blodgett's (1919) “Ship camouflage” 105 years on: A misperception of dazzle perception revealed and redressed Supplemental material, sj-docx-6-ipe-10.1177_20416695241312316 for Blodgett's (1919) “Ship camouflage” 105 years on: A misperception of dazzle perception revealed and redressed by Timothy Simon Meese and Samantha Louise Strong in i-Perception

sj-xlsx-7-ipe-10.1177_20416695241312316 - Supplemental material for Blodgett's (1919) “Ship camouflage” 105 years on: A misperception of dazzle perception revealed and redressed Supplemental material, sj-xlsx-7-ipe-10.1177_20416695241312316 for Blodgett's (1919) “Ship camouflage” 105 years on: A misperception of dazzle perception revealed and redressed by Timothy Simon Meese and Samantha Louise Strong in i-Perception

sj-xlsx-8-ipe-10.1177_20416695241312316 - Supplemental material for Blodgett's (1919) “Ship camouflage” 105 years on: A misperception of dazzle perception revealed and redressed Supplemental material, sj-xlsx-8-ipe-10.1177_20416695241312316 for Blodgett's (1919) “Ship camouflage” 105 years on: A misperception of dazzle perception revealed and redressed by Timothy Simon Meese and Samantha Louise Strong in i-Perception

sj-xlsx-9-ipe-10.1177_20416695241312316 - Supplemental material for Blodgett's (1919) “Ship camouflage” 105 years on: A misperception of dazzle perception revealed and redressed Supplemental material, sj-xlsx-9-ipe-10.1177_20416695241312316 for Blodgett's (1919) “Ship camouflage” 105 years on: A misperception of dazzle perception revealed and redressed by Timothy Simon Meese and Samantha Louise Strong in i-Perception

sj-xlsx-10-ipe-10.1177_20416695241312316 - Supplemental material for Blodgett's (1919) “Ship camouflage” 105 years on: A misperception of dazzle perception revealed and redressed Supplemental material, sj-xlsx-10-ipe-10.1177_20416695241312316 for Blodgett's (1919) “Ship camouflage” 105 years on: A misperception of dazzle perception revealed and redressed by Timothy Simon Meese and Samantha Louise Strong in i-Perception

sj-xlsx-11-ipe-10.1177_20416695241312316 - Supplemental material for Blodgett's (1919) “Ship camouflage” 105 years on: A misperception of dazzle perception revealed and redressed Supplemental material, sj-xlsx-11-ipe-10.1177_20416695241312316 for Blodgett's (1919) “Ship camouflage” 105 years on: A misperception of dazzle perception revealed and redressed by Timothy Simon Meese and Samantha Louise Strong in i-Perception

sj-docx-12-ipe-10.1177_20416695241312316 - Supplemental material for Blodgett's (1919) “Ship camouflage” 105 years on: A misperception of dazzle perception revealed and redressed Supplemental material, sj-docx-12-ipe-10.1177_20416695241312316 for Blodgett's (1919) “Ship camouflage” 105 years on: A misperception of dazzle perception revealed and redressed by Timothy Simon Meese and Samantha Louise Strong in i-Perception

sj-docx-13-ipe-10.1177_20416695241312316 - Supplemental material for Blodgett's (1919) “Ship camouflage” 105 years on: A misperception of dazzle perception revealed and redressed Supplemental material, sj-docx-13-ipe-10.1177_20416695241312316 for Blodgett's (1919) “Ship camouflage” 105 years on: A misperception of dazzle perception revealed and redressed by Timothy Simon Meese and Samantha Louise Strong in i-Perception

## Notes

1. Van Buskirk (1919a, p. 151), Warner (1919a, p. 204) and Williams (2022, p. 76) used the term 'reversed perspective' to mean the same thing.

2. Another remarkable facility from this time was the testing theatre at Eastman Kodak Company Laboratories, Rochester, New York (e.g., see Bekers & De Meyer, 2017; Jones, 1919).

3. In their preliminary report on dazzle camouflage to the Navy Department, Bittinger and Hulburt (1936a) state there was: "[no] statement in the references to indicate the experimental method used by the subsection of Design in developing and testing the [dazzle] designs. True, an elaborate theatre was built for the purpose of testing the designs on small ship models … , but we infer that it was never used, for the statement is made … that the equipment had not been entirely perfected when the work was discontinued, although several trial runs had been made." Unfortunately, it is not clear which test facility this refers to, but it is presumably the one in Rochester (Van Buskirk, 1919a, p. 160)—see footnote 2—and different from the one used by Bittinger and Hulburt (1936a). Whether Bittinger and Hulburt used the same or a different facility from Blodgett is also unclear.

4. Though Williams (2022) states that Blodgett went on to become an accomplished marine engineer and became an editor of the *Proceedings of the Society of Naval Architects and Marine Engineers*.

5. As justified in the Methods section, we assumed that Blodgett's dazzle camouflage was the same on both sides of the ship, as in Lovell et al. (2024). In general, this was not the case for WWI dazzle designs (Van Buskirk, 1919a; Wilkinson, 1917), but that does not undermine our point which relates specifically to Blodgett's stimuli.

6. Although Blodgett (1919) was interested primarily in dazzle camouflage, his writing suggests that that these two designs also employed low visibility techniques (see S1).

7. The small difference (1°) between this figure and the estimate in Figure 6(c) is due to the uneven distribution of trials across various conditions and the difference this makes across grand averaging and pooled averaging.

8. In Lovell et al. (2024), twist effects were reported with an opposite sign convention to those here, because in Lovell et al., the calculations were based on compass angle instead of target angle.
