# Supplementary material for: Blodgett's (1919) “Ship camouflage” 105 years on: A misperception of dazzle perception revealed and redressed
Source: Iperception. 2025 Mar 14;16(2):20416695241312316. doi: 10.1177/20416695241312316 (PMC11909666; doi:10.1177/20416695241312316)
Supplement: sj-docx-1-ipe-10.1177_20416695241312316 - Supplemental material for Blodgett's (1919) “Ship camouflage” 105 years on: A misperception of dazzle perception revealed and redressed [file sj-docx-1-ipe-10.1177_20416695241312316.docx]

**Reformatted version of Blodgett (1919)**

This document is a reformatted version of Blodgett's (1919) MIT thesis (B.S.) on *Ship Camouflage* using Microsoft Word.

The reformatting was done in February-June 2024 by Tim S. Meese (t.s.meese@aston.ac.uk; Aston University, UK).

A pdf of the original work (written up on a mechanical typewriter) can be found [here](https://dspace.mit.edu/handle/1721.1/67156?show=full).

In reformatting this document, every effort has been made to capture the original wording accurately, but the copying process via the clipboard introduced numerous typos that had to be corrected by hand. Any failures in this respect are due to the work of the re-formatter and not the original author.

The original document lacks the conventional organisation of Abstract, Introduction, Methods, Results and Discussion and, following a foreword, is largely a single passage of text with unlabelled figures and tables. There is no References section. As part of the reformatting, some headings have been added and conventional labelling, numbering and brief cross references to tables and figures have been added to the text, all intended to aid reader navigation. Sub-headings of Design 1, Design 2, etc., have been maintained from the original. Other than correcting some punctuation and some original typos/missing small words (*), no further additions or subtractions have been made knowingly (e.g., there is no rewriting to correct poor gramma or to improve clarity), but the layouts of some text, figures and tables have been reworked to benefit presentation. This includes re-grouping several of the camouflage images and colour swatches, cropping of 'white space' from the results figures, superimposing the 'periscope' images of the targets on the relevant results figures, tabulating or numbering and listing some of the text as was befitting, moving Table 2 and the supporting text to before the reporting of the results for Design 1, and moving the results tables to an appendix. Some brief explanatory text has also been included as figure and table captions. None of these captions were in the original.

The original document appears to have a missing page (the original page 34) noted by MIT libraries and as reported in the reformatted version here. It is unclear whether any original material has been lost, but seemingly not. The original disclaimer follows:

**DISCLAIMER OF QUALITY**

Due to the condition of the original material, there are unavoidable flaws in this reproduction. We have made every effort possible to provide you with the best copy available. If you are dissatisfied with this product and find it unusable, please contact Document Services as soon as possible.

Thank you.

Page 34 has been omitted due to a pagination error by the author.

-

The citation for the original work is: Blodgett, L. S. (1919) "Ship camouflage," MIT thesis, B.S., Dept. of Naval Architecture and Marine Engineering. Department of Distinctive Collections, MIT Libraries, Cambridge, Massachusetts.

This reformatted document is to be cross-referenced by Meese & Strong (2025), *i-Perception*, (as Supplementary Material, S1) who present a critical appraisal of Blodgett's work and a reanalysis of his results.

(*) The aim was to leave this as close as possible to the original text without endangering meaning, most particularly when meaning was not clear. But where corrections to punctuation and typos/glitches were unambiguous (in the re-formatter's view), the lightest touch was made, most usually in deleting and in some cases adding commas.

**Notes on numerical errors in Blodgett's thesis**

Several arithmetical errors were identified when transcribing Blodgett's results tables. No corrections have been made to the tables or graphical presentation, but mistakes (typically differencing or averaging errors) are highlighted in bold where > 1 deg. The magnitudes of the errors are not made explicit, but the interested reader will find sufficient information in the tabulated results to work out these details themselves. Where errors are noted in the reported averages, these are deviations from what should have been derived from the results as reported.

*Ship Camouflage*

*by Leo S. Blodgett N.A. Department - M.I.T. thesis 1919*


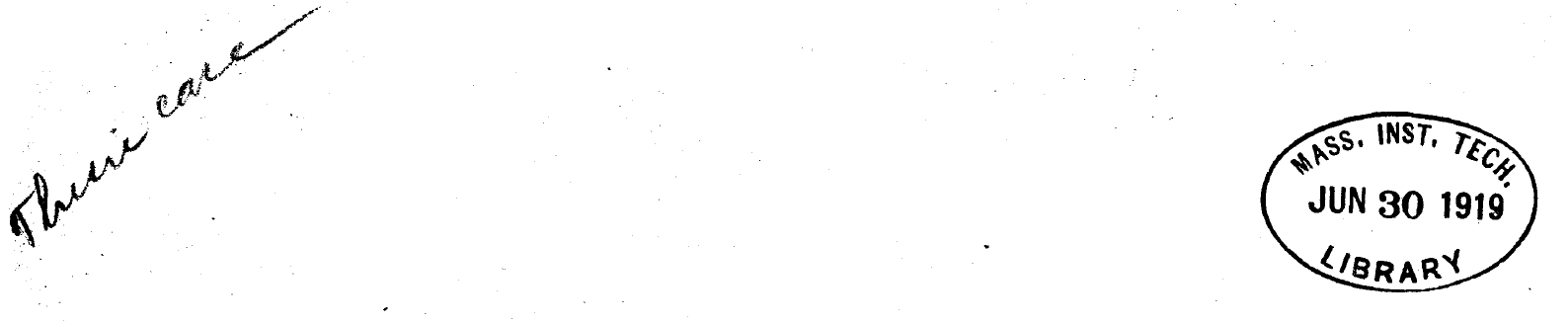
 **Foreword**

The following thesis on ship camouflage is based on the work produced during the Great War, aiming at the apparent distortion of ships by the use of contrasting colors according to the system termed the "British Dazzle". A quantitative treatment of the subject has been attempted with a greater or less degree of success in accomplishing this end. The latest apparatus devised by the camouflage section of the United States Navy was available for observing models, together with practically all data compiled in this country, and reports from Europe. All material was an uncoordinated mass and most of it without practical value in developing the subject. The work of the Navy Department during the last two months of hostilities approached the matter from a definite standpoint and results were just commencing to be apparent when the armistice was signed.

The subject is of very great interest and although it probably will never again come into prominence as a means of protecting ships against the submarine, the results of the work here indicate the possibilities of marine camouflage if approached from a scientific viewpoint.

The writer is deeply indebted to Professor C. H. Peabody of the Department of Naval Architecture and Marine Engineering at the Massachusetts Institute of Technology for his kind suggestions and aid during the work and for making possible the use of the necessary equipment.

Leo S. Blodgett

Massachusetts Institute of Technology.

May twelfth nineteen hundred and nineteen. 114479

**Introduction**

Marine Camouflage, as many other methods of defensive and offensive warfare, had its origin in the early ages of history. When the Greeks went on voyages of conquest their ships were painted blue, purple, green and vermillion, in order that they might be less conspicuous to the enemy. Mention is made in accounts of the pirate ships during the fourteenth and fifteenth centuries of elaborate and conspicuous designs in purple, violet, green, white and other colors, painted on the sides of the vessels. During the latter part of the nineteenth century the navies of the world were painted either black or white. Both these colors had certain qualities that made them undesirable, especially in war times. Germany recognized the need of a neutral tone and to this end had her ships painted a blue gray. England soon followed her lead, then France, the latter using a khaki colored gray. About 1905 the United States adopted a low visibility flat tone gray that is used even to the present on line of battle ships.

Not until the Great War broke out in 1914 and the U-boat menace threatened to wipe the transport and supply ships of the Allies from the seas, did the question of a system of protective painting arise. As land camouflage developed and proved its value in deceiving the enemy, various individuals in Europe and this country, many of them artists of note, attempted to work out similar methods of ship painting. Mackay and Herzog of New York seem to be the pioneers for this important work. About June 1917, keen interest was aroused in the matter both here and abroad. In October 1917, the Treasury Department and the Bureau of War Risk Insurance issued an order requiring all ships travelling under supervision to be painted in a manner which should prove of protective value while crossing the danger zone. A penalty of one-half percent increased insurance was imposed if instructions were not followed. The Submarine Defense Association, organized by shipping interests in New York, attempted to have all ships coming within its control painted with some design approved by the Government. To aid research for designs, the cooperation of the Eastman Kodak Laboratories at Rochester, New York was enlisted for carrying on experiments in Low Visibility painting.

Until March 1918, practically all systems attempted to lower the visibility of ships at sea by painting them out of the skyline. While research in low visibility was being carried on in this country, Lieutenant Norman Wilkinson R.N.V.R. discovered an entirely new and effective system of camouflage in England. This was called the British Dazzle and replaced all preceding ideas on the subject of protective coloration for ships. Designs were immediately sent to Washington from the Admiralty, and Wilkinson made a short visit to the Bureau of Construction and Repair to initiate certain Constructors in what was then known of the subject. Two Lieutenants, Jones and Van Buskirk, were designated as the organizers of a Department of Camouflage under the supervision of the Bureau. The Shipping Board appointed various individuals, mostly artists of more or less merit, to carry out designs on the ships as they might be supplied by the Navy Department and to attempt the development of the subject using such means for research as could be made available. The Dazzle System was established as the basis of all designs. To this end, each of the Camouflage Districts developed and improved apparatus for studying the subject under conditions which would simulate actual weather at sea. At first the apparatus was anything but satisfactory, consisting of a periscope with a minus lens and mirrors through which models might be studied when placed before a screen on which sky scapes were painted. Later the Boston District worked out what is considered by the men interested, a theater of observation that is strides ahead of any similar theater in the country. It includes all essential features of sea and sky illusion, light effects, periscopic means of observation comparable with actual conditions. This apparatus has been used in developing the subject matter of this thesis, so a description may be given at this point.

**Methods Part I: Equipment and procedure**

The question of the illusion of water was first considered, and it was solved by building up a curved painted surface, which most nearly approached the appearance of the sea as observed from a submarine. An endless belt of canvas painted with various sea scapes was run over rollers in order that a variety of weather might be simulated (see Figure 1). The scenery for sky was treated in a similar manner in the vertical plane so that various conditions and shorelines might be obtained. Hand cranks were attached to operate the canvas belts. The bed over which the sea canvas rolled was given a downward turn in order that a true representation of water in the horizontal plane would result and the visual center of the periscope brought low enough to the horizon to approximate the relationship existing under actual observations.


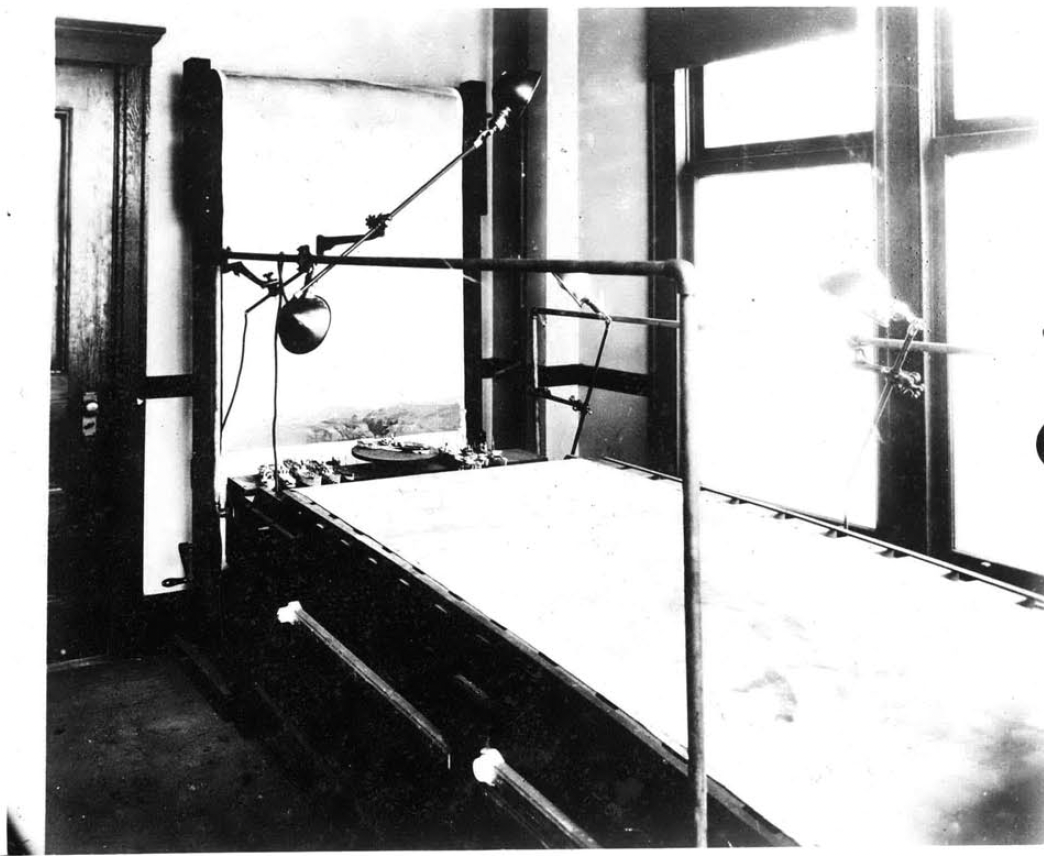

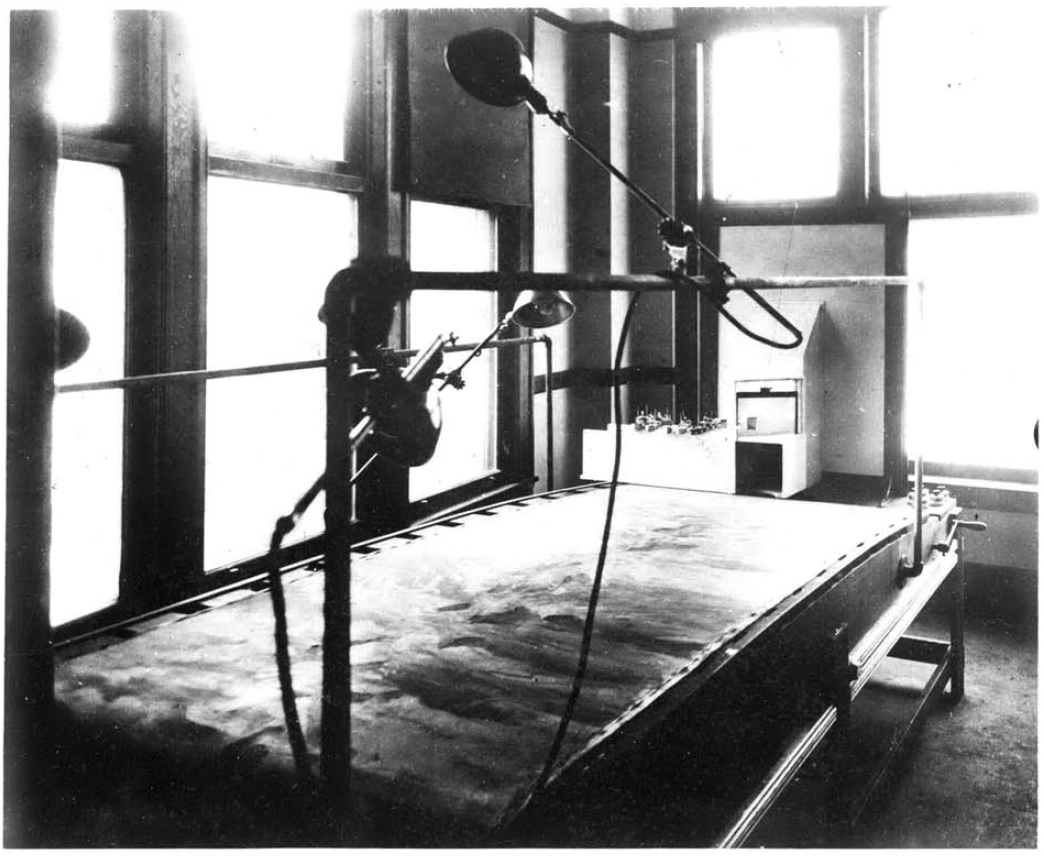


Figure 1. Two views of the equipment of the Boston Camouflage District used in the experiment. (This atribution is the best that can be discerned from the original document but from that source, uncertainty remains over where the experiments were conducted.)

In the case of the periscope, it was not possible to follow the true one exactly for it was found necessary from the size models used to produce a virtual image, reduced in size, where actually a real image enlarged would result. The models were made to such a scale that at distances available they gave a true representation of the ship. To render conditions of observation rigid, the ranges were made 1100 to 2200 yards, which are somewhat less than most cases of attack at sea. Two mirrors and two lenses comprise the working parts of the periscope, the mirrors being fixed parallel to each other and at a forty-five-degree angle with the vertical the lenses placed between the mirrors. The lower lens is fixed and has a diameter of four inches with a focal length of thirty, while the upper lens is a three-inch minus or reducing lens, which will allow the image to be varied in size as desired. A fog producing device was installed directly in front of the bottom opening of the periscope. It consists of a semi-transparent mirror placed vertically at a forty-five-degree angle to the line of vision. A ground glass screen is placed in the vertical plane parallel to the line of vision. Fog densities may be varied by adjusting a light enclosed near this mirror. Blue and ground glass screens are used to give the correct tone to daylight fog. Horizontal light carriers were erected along the sides of the frame, and nitrogen, daylight lamps used for effects on the sky and sea.

A turntable was installed between the horizontal edge of the sea and sky scenery and so arranged that as it was turned by a hand wheel, the indicator on a compass card pointed in the true direction taken by the ship. This card was conveniently located near the eyepiece of the periscope so that as estimates of direction were made by the observer on another dial, the two might be compared directly and the amount of error ascertained. Models were constructed on a scale of 1/32" : 1 Ft., as this dimension gave a true representation of the ship as observed through the periscope.

The apparatus is without question the nearest approach to conditions as they might occur at sea and shows a great deal of study on the part of the men interested in the matter in developing it. However, there are various defects and drawbacks that should he noted. No definite scientific study of the details of the apparatus was made. The curve of the sea belt, the arrangement of the periscope, lighting, the painting of the sea and sky, all were determined so that they appeared natural to artists familiar with sea painting. A little too much was left to the artistic temperament and imagination of these men. Nevertheless, if models are tested rigidly, leaving as little as possible to the imagination of the observer, good comparative results may be obtained with the equipment.

**Submarine attacks**

An idea of the appearance of a ship, as seen through a periscope, as well as a general description of the instrument as used by the Germans, is necessary in a treatment of the matter of Camouflage. For torpedo and gun fire attack, accurate observations of the speed, range and course, of a vessel must be made. In the case of torpedo attack, all such observations are made through the periscope, for the submarine is usually running below the surface, while for gunfire, after the ship is within range, the accuracy of shooting is determined by spot-firing. Periscopes are fitted with range finders, but range, as well as course and speed, is rather uncertain. To allow for error, ten degrees in course, and two knots in speed are incorporated in plotting the position of the ship. An error of two thousand yards at a range of ten thousand has been noted in the log of one British submarine.

Dr. F. Weidert a German periscope expert expresses the difficulties of range finding from a submerged U Boat.

"Now it is already well known that correct estimations of range with the naked eye, without some means of assistance is extremely difficult, and for many people actually impossible. With one eyed vision, through an optical instrument, this is even more the case. On the other hand precise knowledge of the range is essential for many purposes, especially for the firing of torpedoes. To unite with the periscope a range finder, of sufficient merit meets indeed with serious difficulties, inasmuch as one cannot apply apparatus whose use will cause one to be prematurely seen by the opponent."

(Entwicklung und Konstruktion der Unterseebootensehrohr.)

To estimate range from a periscope there are two available methods, one to project into the eyepiece of the instrument, crosshairs divided in hundredths both vertically and horizontally, or telemeter scales. The Goerz double imagemicrometer may be used as well. Lindell T. Bates describes the system in his report, "The Science of Low Visibility".

"Two pictures of the same object are made to cut each other in the lens, and are shifted with reference to one another until the tops of the masts or other high points of a ship under observation in one picture are level with the water line in the other. The angle of shift is measured to determine the distance. This method too is only roughly approximate, for it has to deal with the measurement of a very small angle, and it assumes the height of the selected part of the vessel, and uses this figure for the length of base line."

In all methods used, the ship is in motion and estimates of her length on the cross hairs are necessarily very approximate, unless the observer has had long practice. Estimates in course depend on the alignment and perspective of masts, funnels, and superstructure. If the ship takes a zig-zag course it is hard to make correct estimates, and if her masts and structures are out of line either by actual construction or by perspective painting, the difficulties are greatly increased. Speed calculation depends on the triangulation method of observation, which in turn depends on the accuracy of range estimate. If the latter is in error, the speed will also be incorrect.

**Camouflage against submarine attacks**

The efforts of the Submarine Defense Association in developing a Low Visibility painting for ships, deserve a description at this point. Lindell T. Bates and Loyd Jones, carried out extensive experiments in the Eastman Kodak Laboratories, studying colors, and combinations of color that would blend to a flat tone at a given distance when applied in areas of definite proportions. Actual tests of gray painting on models were made on Lake Ontario and on the Atlantic. The U.S.S. Gem, a 125 ft. patrol boat, was assigned to them by the Navy Department for such tests as might have a bearing on the matter. Under a range of 5,000 yards, in clear weather, low visibility is beyond hope of attainment. Colors may blend at shorter distances, but do not give the desired tone. No flat tone pigment or paint of any description will totally absorb the light that falls upon it, and the reflective quality however slight destroys the desired effect under 5,000 yds. At this range the ship will actually fade into the skyline if painted a gray, or with such colors as will blend to gray; that is unless the ship happens to be between the sun and the attacking submarine. In which case it is impossible to hide it. Mackay and others who developed the low visibility idea, combined colors in definitely proportioned areas, and obtained a resultant gray. Any colors as red, green, and violet, that superimposed will give a white, may be used. Bates and Jones determined that a shade of gray known as Omega Gray, was best adapted for use in the northern part of the danger zone, where the weather is about seventy percent cloudy. South of Lat. 45 degrees North, a gray of bluer tone, called Psi Gray was best. Combinations of color to give these resulting grays were Alpha blue and Beta White in equal parts for Omega Gray, and Gamma Blue and Delta White for Psi Gray. Very little difference in the result could be noted between colors applied in mixed areas or painted on as a monotone. All of which would lead to the conclusion that the desired gray be painted directly on the ship, and since such painting has not proved particularly effective as camouflage, all the work done in the matter has to a certain extent been futile. No ship however invisible can remain so very long because of the remarkable hydrophone equipment carried on all submarines. With the apparatus in the hands of an experienced operator, it is possible to detect the presence of a ship at a range of ten or twelve miles, and to determine her approximate direction. The submersible would approach her until observations on her could be made through the periscope.

**Dazzle camouflage**

The greatest efforts have been carried on investigating systems of dazzle as invented by Lieut. Wilkinson. Any system that is to have a degree of merit must distort the ship at a range of 1000 yards or less, so that accurate observations may not be taken at that point. By painting the ship with bright or contrasting colors, outlines and structures will be broken, and if the design is good, the range, course, and speed of the vessel will be in doubt, thus causing the submarine to remain near the surface longer than is altogether safe for her, while corrections in plotting are made. Her painting must be such that a maximum distortion be obtained under all conditions of weather and light. From the slight inaccuracy of the periscope, in combination with the effect due to the human element, an error of 15 degrees in course will be sufficient to accomplish the complete upsetting of torpedo control from the submarine. If in addition an error of 2 Knots in speed or of 200 to 300 yards in range be attained, the sub is effectively blocked as a menace to shipping so far as torpedo attack is concerned. Shell fire on the other hand necessitates the submersible coming to the surface and resorting to spot-firing. Then the only defense of the ship attacked is her armament and the possible help of destroyers. A surprising lack of really good information on the matter is very evident, and not until the last two months of hostilities was apparatus developed that could in any way be depended upon to even approximate actual conditions. A few good principles were discovered, and all systems of painting must depend on these. Nevertheless, with few exceptions these principles have been completely ignored and the individual camoufleur given free rein to his imagination, and the results of his work applied to ships regardless of merit.

It would seem obvious in examining the matter that certain principles of distortion and perspective worked in a definite scheme, and with colors of sufficient contrast would accomplish all results desired. Then if the tests on such systems be reduced to as nearly a mathematical basis as is possible with so indeterminate a matter as obtains under sky and light conditions at sea, the results would be of value.

**Motivation for the experiments**

All experimental work of this thesis has been based upon principles of distorted perspective in combination with colors approved by the Navy Department and which are not affected by color screens to any appreciable extent. An effort has been made to reduce the matter to a mathematical basis. With perspective designs, any desired concentration of dark or light color may be made in order to satisfy the principle that large masses of color at bow, stern and superstructure, have a distorting effect. It is a fact that no actual perspective design appears in the approved list of the Bureau until nearly the end of the war. Then several such designs were distributed for application to ships, showing that the value of the method had become apparent. As a matter of fact, it had become certain towards the cessation of hostilities that the effectiveness of the submarine as a weapon of offense had been greatly lessened in value because of the perfection of the hydrophone as applied to fleets of small, swift craft, in conjunction with the use of the depth charge. A submarine in motion, within the range of a listening device was surely doomed. The ferrets of chasers could find her and stay with her until an "egg" could be successfully dropped, when she might either come to the surface disabled, and surrender or lie to on the bottom and perish. In spite of this fact, the subject has a certain fascination, and the work of this thesis will strive to bring out certain points that may be of value, if the matter should ever come into prominence again, as it will probably never do. There is the possibility of sound and vibration deflectors being used by submarines, so that the effectiveness of the hydrophone may be greatly lessened. Such general principles as proved of value in research have been used here and will be incorporated in the aims of the work, while in addition an effort will be made to prove the value of certain basic principles of perspective painting.

**Methods Part 2: Experimental conditions and approach**

The apparatus described in the first pages has been used in such a manner as to simulate actual conditions and to reduce the results to as nearly a scientific basis as the rather vague and artificial conditions will allow. In general, the apparatus has four distinct sky scapes:

(i) clear blue sky,

(ii) hazy, dull sky, in which cumulus clouds predominate,

(iii) dark storm clouds, touched with color including tints of orange, yellow, and patches of blue,

(iv) the typical ragged shore lines that occur along the English and French coasts.

The sea scapes have the effect of toning the sky and reflecting lights on the ship but do not occur directly as a background. In this case, four types of sea are painted on the canvas belt:

(i) calm blue, summer sea,

(ii) bright green water, slightly ruffled,

(ii) dull gray hazy sea,

(iv) rough white capped choppy weather.

It is very difficult to determine exactly which sea or how many different seas may be combined effectively with a given sky to seem real and have the basis of possibility. A short table of effects used in tests made will show the difficulties of the matter.

It was assumed that the following sea scapes (Table 1) might occur with a given sky.

| Sky | Sea scape |
| --- | --- |
| Clear blue sky  It is very important consider angle from which sun strikes the ship | On a calm day with a slight haze, the sea might be blue; then with hard metallic sunlight, a green sea might result; and on a clear, windy fall day, a choppy blue sea would occur. |
| Hazy sky | All four effects of sea might occur if the lighting were properly regulated. |
| Storm sky | Either the dull, flat sea if the wind had not ruffled it, or if the squally or puffy winds were blowing, the dark rough chop would result. |
| Shore line | Practically any one of the four might occur with proper lighting. |

Table 1. Links between sky scapes and sea scapes (see text for further details).

It must be remembered that the play of light is ever changing, toning down in places, increasing brilliancy in others, and at every change having some effect on whatever object appears on the face of the sea. To extend a series of observations on actual painted ships under such conditions would be long, tedious, expensive and of doubtful accuracy, because but one or two ships could be obtained for such experiments. In trying to attain some reliable simulation of actual conditions on painted scenery and artificial light, one is convinced of the rather hopeless aspect of the task. However, the work will be carried on using any little knowledge acquired at sea and along the New England coast, of lights and shadows, applied to the apparatus at hand. As many combinations of sky, sea and light will be used as seem possible, and because it is not practicable to use a moving model and a given range, there remains but one object to attain, that of altering the course. It is true that if this can be done successfully, the range and the speed will almost automatically be changed. Although there are many atmospheric factors entering the problem of the ship at sea, and all of them, as refraction and haze, helping towards a distortion, since the tests under artificial conditions must be made as severe as possible, an alteration of course of not less than 18 degrees must be made by the painting.

***Design, participants, and more procedure***

Two angles for each design will be judged by each of six observers and under the four different skies, with changing sea and light. It will be attempted to have no two scenes alike that is either the light or the sea will change, and the resulting shadows will tend to alter the aspect of the ship. The actual angle of progress will be noted, the estimated angle, and the error, in tables prepared. Errors for each ship will be averaged for each observer, and compared, then the average of all the observers taken. This seems to be the only way of reducing the matter to anything near a fair basis. A study of the methods of precision of measurements, although usually understood to apply to direct mathematical measurements, may also be applied to indirect measurements, and the case in hand, is comparable to certain calculations entailed in chemical combinations where precision has been applied. The final average angle of deviation on each ship will be used as the basis of the effectiveness of design. Distances at which observations are to be made must be severe in trying the ship, so ranges of 1100 yards in four cases and of 2200 yards in the other two have been chosen. Actually, very few attacks at such range would occur. Any attempts at low visibility dazzle seem rather impossible here, but in one or two cases tried that prove of value, the results will be analyzed for a possible bearing on the subject.

Fortunately, the first observer is a Lieutenant in one of the European Navies, and is entirely familiar with ships, periscopes, range finders, and conditions at sea in general, under which observations might occur. Before estimating course, he became familiar with each design so that any errors made will be in spite of a previous knowledge of the ship. Repeated observations on a design with no limit to the time allowed would seem to ably care for possibility of the submarine commander becoming familiar with a given design. If errors are persistently made and greater than the required 18 degrees, the object would certainly be gained, especially at sea with atmospheric conditions aiding.

The second observer has been very closely in touch with the evolution and application of each design, and has made repeated observations on them, in fact offered valuable suggestions in the progress of the work.

The third set of observations was made by four different individuals. None of them was familiar with the painting previous to his observations on the apparatus, so that all were unbiased by a previous knowledge of what to expect from a design. All of these last are entirely familiar with ship structures, with principles of perspective and optical illusions, as well as conditions of light and shadow at sea. Conditions of observation have as stated been made as severe as could possibly be expected, and results obtained will indicate fairly the merits of failure of the following principles.

***Further observations on camouflage/stimulus design***

From reports and data available it is certain that camouflage must accomplish the following results. All high vertical points, as masts, stacks, superstructures, must be distorted so that no estimate of course can be made by lining them up. To accomplish this, one mast alone should be used, and that as short as compatible with the efficient operation of the wireless. If two or more masts are absolutely necessary, they must not be placed on the center line of the ship, but staggered. The stacks must be short, if necessary forced draft should be resorted to, in order that the desired result be accomplished. False superstructures may be used, but it is better not to depend on them, for they must be of rigid construction to stand weather, are cumbersome and more or less of an expense to construct. To aid in preventing the lining up of masts, corresponding parts should not be painted the same color. That is, the tops should not both be white, or both black, if these two pigments were to be used. One top should be white, the other black. Now at no time will both be plainly visible, one will blend more or less with practically any background. This is increasingly true of colors to be discussed later. There are two methods of concealing superstructure and false works, and between them there is little to choose in results obtained. One is somewhat more simple of application to the ship than the other. Any scheme of distortion or perspective painting on the hull should be adequately and consistently carried into the superstructure. The other method depends on black or some other dark color as violet for its effect. In this case the entire structure, the forward and aft vertical surfaces, the lower part of the stack, are all painted the dark monotone, and the result is quite remarkable in that it is almost impossible to determine angles or lengths. This method really carries out any hull design and more or less accentuates contrasts. The top of the stack should be painted with the lighter tones used on the hull. In approaching the treatment of the hull, a word of the lines on which the Bureau was proceeding at the time hostilities ceased. The ship was considered to have three main divisions: bow, midships, and stern. The bow is made to turn away in the painting by the use of light, pale colors, in small divisions. The midship section is devoted to breaking of outlines. The stern is made to turn towards the observer. Production of shapes in the three divisions should be made with three things constantly in mind.

a) That there should be a continuous perspective design through the three spaces.

b) That the important forms be constructed with a large and a small end, and the small end painted forward.

c) In every case where it is possible, that all important lines point downward from the stern to the bow.

This last may be questioned in the light of experiment and will be considered later in the results obtained. Instead of trying to imitate the stern on the bow, as often practised, the stern should be brought forward to the quarter.

These principles are the basis of the work here, that while breaking of outline is absolutely necessary it is not essential to blotch colors on the side of the ship in contrasting masses in an artistically hit or miss way, but all effects striven for may be combined with some phase of application of principles of perspective. Any color of desired intensity may be applied at any designated part of the ship but in such a way that it has a definite part in the scheme of things. Lines may be smooth but not monotonous, that is simply because a line or painted strip may be followed by the eye it may not be possible to determine direction, particularly if it is given a turning movement. The same curve appearing concave at one moment with a very slight change of light will seem to be convex. When contrasting colors are arranged in any good perspective design it is practically impossible to determine the ship's course accurately.

Twenty-four designs have been used in this work, and all of them employ some perspective or converging line principle. No two are in any way similar and might be painted with practically any one of the combinations to be discussed later and be of value. The best twelve designs will be carried through a complete discussion, and conclusions as to their respective merits drawn. All observations will be made under the foregoing conditions.

In deciding on colors that may be used advantageously it is safe to discard brilliant colors. All those described will be designated by standard Navy Camouflage terminology. Twenty flat tone pigments have been prepared and tested. Some of these have been discarded, while those used are as follows:

Black, White, Grey white, No. 4 Gray, No. 1 Gray, Grey pink, No. 3 Pink, No. 1 Gray green, No. 1 Green, No. 1 Blue, No. 3 Blue, Lavender, Violet, Yellow (see Figure 2).

Figure 2. The colours used for the ship camouflages in the experiment.

Any intensity or tone with this range has been possible, but none of the designs have made use of other tones than those indicated. Decks have in each case been painted with No. 1 Gray. It will remain for the results of experiment to give various combinations in which these colors may be used effectively. Designs have been made as simple as possible with the idea always in mind that they have to be applied to the side of a vessel. As few colors have been used in a design as will attain the object of contrast.

**Results and camouflage descriptions of stimuli**

Figure 3. Designs 1-6 (out of 12) used in the experiments. Designs 1-3 are applied to tramp steamers with superstructures and stacks placed aft. For Designs 4-6, the superstructure and stacks are amidships.

|  | **Black** | **Gray** |
| --- | --- | --- |
| **Clear** | 2 | 2 |
| **Hazy** | 8 | 4 |
| **Storm** | 5 | 3 |
| **Cliffs** | 3 | 7 |

Table 2. Results for Black and Gray ships (different columns) as they would be painted in normal times.

The results in Table 2 are plotted with those for each design (Figures 4 & 6) in order that a direct comparison can be made in each case.

***Design 1***

This design (Figure 3, top-left) is the simplest possible application of perspective. All lines converge at a point either actually, as the arrowhead at the bow, or the bounding lines of the other figures produced. The masts are treated so that the gray and the pink divisions will at no time stand out from a background simultaneously. The gray patches from the hull are produced to stack and superstructure. At first glance the choice of the grey and pink would seem rather poor, but they have a very decided degree of contrast and have proved most effective in actual tests.

The photographs of the various models (insets to Figures 4 & 6) give a slight idea of the ship as observed through the periscope. It was impossible to focus the camera through the periscope lens, so the pictures were taken from a slight elevation; that is the camera lens could not be brought close enough to the water level to absolutely eliminate the sea from the background. Because of this fact it is possible to guess the direction travelled by the ship much better than if it appeared wholly against the sky.

These photographic reproductions represent the maximum effect that can be attained by the use of color screens. In order to approximate this effect in actual observations, it is necessary to devise a system of screens that in a very short interval of time will eliminate the red and green tones from the ship and cause it to approach a flat painted surface in appearance. Observations may not be prolonged more than thirty or forty seconds.

In this case (inset of Figure 4, top-left) the single mast is quite effectually blocked out of the skyline, with the exception of the very top, which would not be useful in determining the size or course of the ship. The design is simple, but the actual effect of the colors is necessary for distortion.

Ship is headed 30 degrees away from the observer.


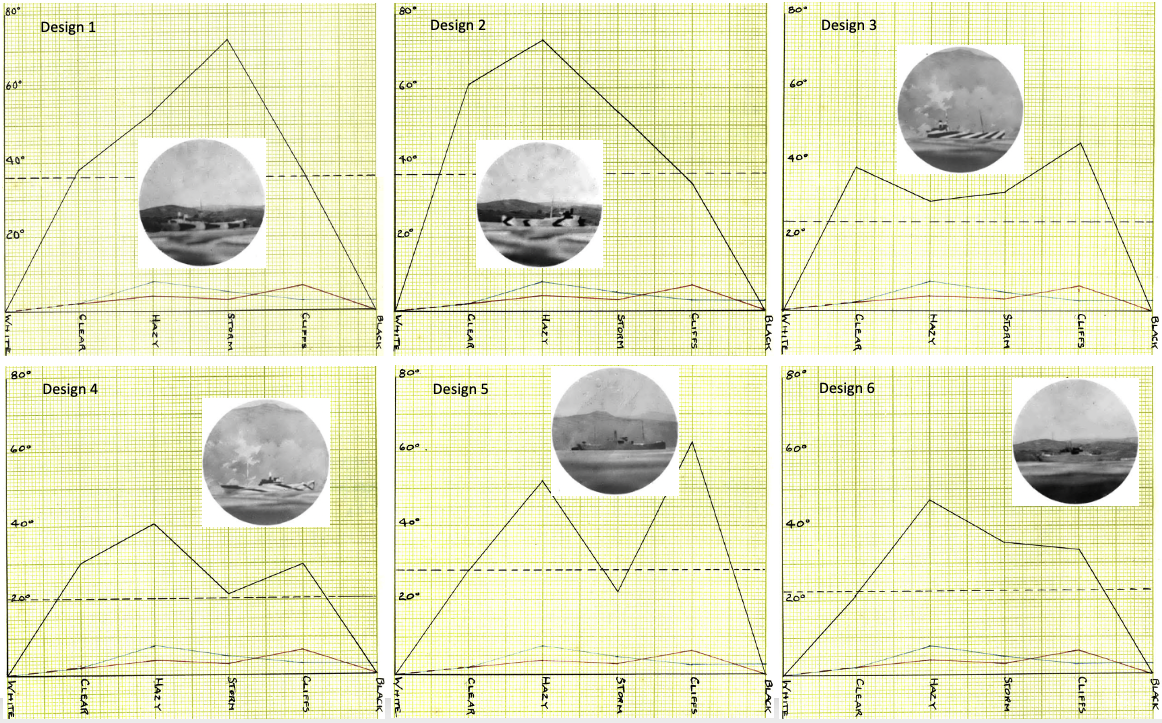


Figure 4. Results for Designs 1-6. The ordinate gives the average error from Tables A1-A6 in Appendix A for six backgrounds which are from left to right: White, Clear, Hazy, Storm, Cliffs, Black. The errors were assumed to be zero for the black and white backgrounds, but no data are reported. The dashed line is the average across background conditions including black and white. The blue and red lines are for black and gray ships, respectively (see Table 2). The insets indicate views similar to that seen through the periscope (see text for details). The ship directions in the insets are: D1: 30 deg away; D2: 30 deg towards; D3: 30 deg towards; D4: 20 deg astern; D5: 25 deg towards; D6: 20 deg towards.

A word is necessary in explanation of the method of plotting the results of observations so that a direct comparison of designs may be made (Figures 4 & 6). The average angle of error for each sky has been plotted against that sky as abscissa. With a white background the error has been assumed to be zero, which assumption is borne out by actual test. It will be noted that starting with white, the sky scapes are graduated as clear, hazy, storm, cliffs and black. In the latter case the error is assumed to be zero. The areas under the straight line plots are a quantitative measure of the effectiveness of the designs as camouflage, and by comparing them the relative merits of designs are clearly brought out. The results of observations on black and gray ships as painted in a monotone (Table 2) are incorporated in each plot, so that the camouflaged ships may be compared with these. The solid black curve indicates errors in the case of the camouflaged ship. The broken black line indicates the average under all conditions of weather. The blue line represents the errors in the case of the black ship. The red one, errors made in estimating course for the gray one.

The results for Design 1 are shown in Figure 4 and Table A1.

***Design 2***

It was determined that black, blue and white had a maximum contrast, and in several designs they have been utilized. In this case (Figure 3, top-middle) they are applied in the converging line principle, in such a way that the peaks of all areas cannot be lined up, that is the eye does not follow from one point to the next directly. It is very difficult to determine the shape or size of superstructure when painted black, and here the broad stripe of this color carried from the hull to the superstructure effectually blots the out lines. No. 2 Gray white was incorporated with the idea that against certain skies and under various weather conditions, the sections so painted would be lessened in visibility in the same proportion that the contrasting parts would attract the eye, thus breaking the outlines.

-

NOTE: The disclaimer by the MIT Library mentioned at the beginning of this document was placed here.

-

From the inset for Design 2 in Figure 4 (top-middle) it might be assumed that the ship was approaching the observer at an angle of maybe ten degrees from the horizontal. Actually, it is much greater than this, being 30 degrees. See Figure 4 and Table A2 for results.

***Design 3***

In this case (Figure 3, top-right) all lines on the hull converge at a point forward of the ship and a little above it. This vanishing point system used in nearly all cases is most effective in obtaining the desired turning movement of the ship. The narrower lighter stripes at the bow also tend to turn it from the observer, while the heavier striped stern brings it into prominence and towards the observer. Black is used to paint out the superstructure and the masts are painted in the accepted manner. No. 1. Green has been used for the sake of variety and in order to test its value, but No. 3 Blue might as well have been used. It is possible to screen out the green, but the resulting black will serve very nearly the same purpose in distortion.

From the inset for Design 3 in Figure 4 (top-right), the ship would seem to be headed away from the observer. It is coming ahead at an angle of 30 degrees. The masts are out of line, which helps the distortion. The design indicates very well the effect of lines converging at some vanishing point, and also that even a simple one may give excellent results. See Figure 4 and Table A3 for results.

***Design 4***

The first three designs were painted on a tramp steamer having her superstructure and stacks aft. Because of this fact, the distortion was rather harder to affect than in the present case (Figure 3, bottom-left) where the structure comes amidships. Two vanishing points, one above the bow forward of the ship and the other below, have been used. It is intended that the bow turn away, and the stern towards the observer. The combination of black, No. 3 Blue, and White is good in all designs, the black and white for contrast, and the blue because it is very difficult to focus the eye upon. Here the design is carried from the hull into the superstructure. The results for Design 4 are shown in Figure 4 and Table A4.

Here the double vanishing point turns the ship farther away from the observer than it actually is (inset to Figure 4, bottom-left). Its true direction is 20 degrees astern, but it might be anything up to 45 degrees.

***Design 5***

Low visibility has been discussed at considerable length in the foregoing pages, and it was decided that under a range of 5000 yards it was not effective. The present design (Figure 3, bottom-middle) was made with the idea of attempting to dazzle by the use of colors in themselves of comparative low visibility. Beyond 3000 yards, however, the colors blend and the monotone, while nearly invisible against some backgrounds, has in general the faults of monotone painting. The design is built from the arcs of circles, and if other colors had been used to give contrast it would have been effective at all ranges. As it is, however, the interest lies in its distortion to the range of 3000 yards. Superstructure is included in the hull design and masts are properly treated.

The gray and green used here blend to a flat tone on the plate and quite effectively alter the course (inset to Figure 4, bottom-middle). This gray is the result of using two colors of marked contrast yet even with the naked eye at a range of about fifteen hundred yards, the colors have a tendency to blend. Actual direction: 25 degrees towards the observer. See Figure 4 and Table A5 for results.

***Design 6***

Here again (Figure 3, bottom-right), contrast is obtained by means of colors that were never considered in the research work during the war. The design is one of reverse perspective. Violet applied in broad stripes at the stern, when carried into the structure acts as effectively as black would under the same conditions. It is a very simple design, easily applied to the side of a ship, and effectually breaks up the outlines.

In this case (inset to Figure 4, bottom-right), as in the last, the gray results from a combination of contrasting colors, dark purple and bright green. Both examples of low visibility show the good qualities of this method of painting, that is the ability to blur outlines by their use. Maximum effects, however, are attained with colors of more decided contrast.

Actual direction 20 degrees towards the observer. See Figure 4 and Table A6 for results.

***Design 7***


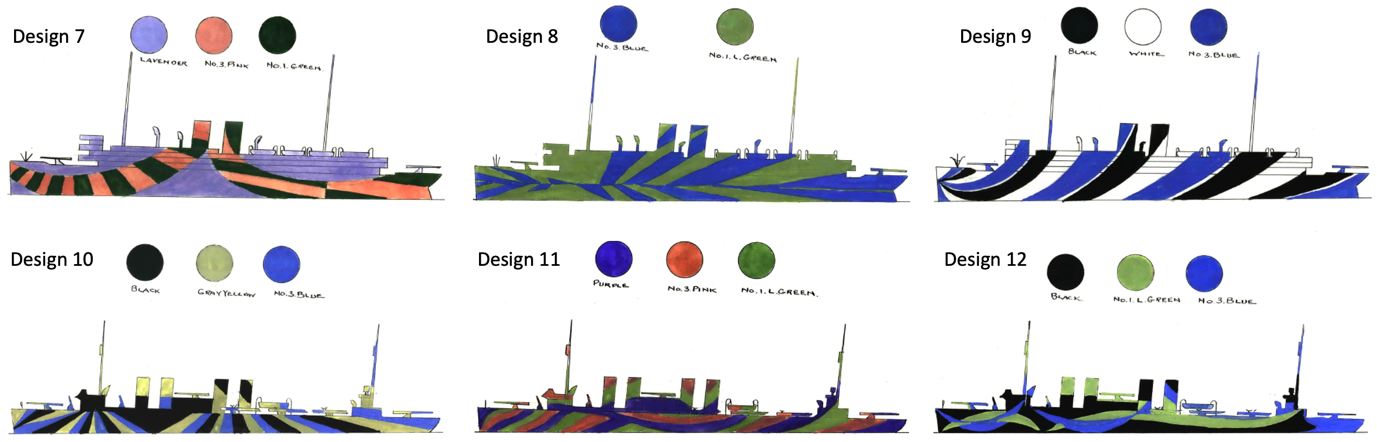


Figure 5. Designs 7-12 (out of 12) used in the experiments. Designs 7-9 are on passenger ships and designs 10-12 are on destroyers.


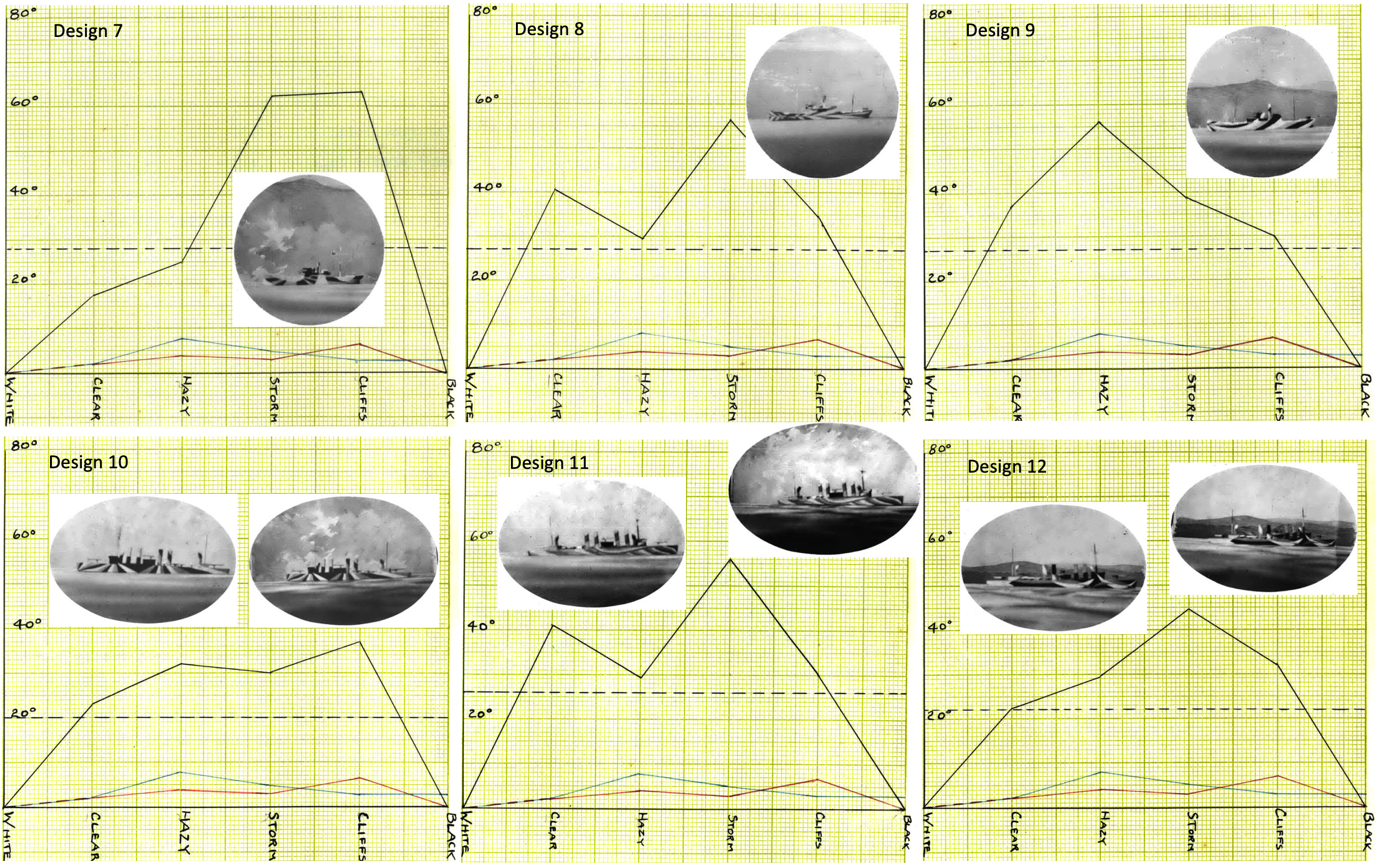


Figure 6. Results for Designs 7-12. The data are in Tables A7-A12 in Appendix A. See caption to Figure 4 for details. The ship directions in the insets are: D7: 30 deg towards; D8: 30 deg towards; D9: 30 deg towards; D10: 25 deg astern; D11: 20 deg towards; D12: 30 deg astern.

Mackay determined that red, green and lavender, applied in small areas would give a resulting gray tone of warm hue, and would attain the effect of low visibility. The same colors applied to the passenger ship of the type here considered (Figure 5, top-left) in sufficiently large areas gives a very good result in dazzling the observer. All lines converge at a vanishing point near the bow. The lavender attracts the eye less than the brighter colors, green and pink, and has the effect of blotting the outlines. The curve at the bow has a tendency to twist it away from the observer.

The combination of curves and vanishing points actually turns the ship away, very effectively in natural colors, while here the camera has nearly neutralized the effect (inset to Figure 6, top-left). Actual direction 30 degrees towards the observer. See Figure 6 and Table A7 for results.

***Design 8***

No. 3 Blue and light green used here (Figure 5, top-centre) have not the great degree of contrast that some other combinations will give, but as used in this type design, which might be designated a herring bone perspective, they give rather good results. The point near the bow at which the two sections of design converge seems to break the bow apart from the ship, and as observed under certain light, turns away from the periscope, while at others turns towards the lens. Superstructure is effectively distorted by carrying on the hull design. The rather large masses of blue at the stern render the actual length of the ship vague.

The pattern has been decidedly altered on the negative (inset, Figure 6 top-middle) while actually the light parts of the picture are a medium blue on the ship, and the dark are light green. In spite of this change the contrast is about the same. Actual direction 30 degrees towards the observer. See Figure 6 and Table A8 for results.

***Design 9***

Another combination of black, No. 3. blue and white in large regular curves, proves effective in application to this type of passenger ship (Figure 5, top-right). All curves working towards the point at the bow twist the bow away from the observer.

This is a very good example of the twisting effect of long curves converging at a point either on the hull or at a vanishing point outside away from the ship (inset, Figure 6, top-right). Actual direction 30 degrees towards the observer. See Figure 6 and Table A9 for results.

***Design 10***

Destroyers are rather difficult to successfully camouflage because of the small amount of freeboard and the massive superstructure and stacks, all of which have a decided rake aft (Figure 5, bottom-left). This first destroyer design employs extensive areas of black to serve the end in distorting the structure. It is very nearly impossible with this design to determine the type of ship or direction travelled. No outlines are sufficiently clear to determine the course. It is a rather successful combination of the black, No. 3 blue, and yellow.

It has been noted that it is difficult to disguise a destroyer or to alter her superstructure. The three following designs indicate the possibilities of distortive painting in this connection. The actual colors of course render more marked the effect that is indicated in the pictures. Actual direction 25 degrees astern (insert, Figure 6, bottom-left). See Figure 6 and Table A10 for results.

***Design 11***

Here is another case of pink, green and lavender applied as a dazzle (Figure 5, bottom-middle). As a matter of fact, the design has a tendency to lower the visibility of the ship under certain lights, even at ranges of 1100 yards. Whatever effect is not gained by lessening the visibility is accomplished by distortion so that the results attained make it acceptable. The outlines are rather well broken.

It was believed that this painting would prove of low visibility when photographed (inset, Figure 6, bottom-middle). Against certain backgrounds the visibility is reduced. The results here are passable but not as good as in some other cases. Actual direction 20 degrees towards observer. See Figure 6 and Table A11 for results.

***Design 12***

Again, the lavish use of black aids the design in breaking up the form of the superstructure, while the long regular curves in contrasting colors give the bow a decided distortion (Figure 5, bottom-right). The No. 3. Blue stern prevents the eye from determining the length of the ship while the mass of black on the quarter by attracting observation serves to shorten any estimate. Other color schemes for the foregoing designs will be discussed in the conclusions.

This painting indicates how effectively the ship may be distorted and superstructure broken by the use of black and medium blue. It is difficult to determine the length of the ship either in the photograph or in observing the model through the periscope (inset, Figure 6, bottom-right). It is equally hard to decide on the number of stacks and the disposition of deck works. Actual direction 30 degrees astern. See Figure 6 and Table A12 for results.

**Discussion and Conclusion**

In taking this picture (Figure 7) it was hoped that a comparison of two destroyer designs would indicate how great a distortion may actually be attained. The ships were placed about

six inches apart and as nearly parallel as possible. The results of the camouflage are self-evident.


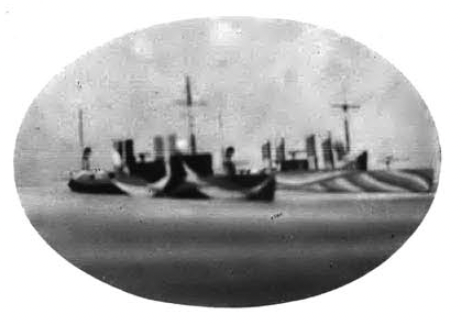


Figure 7. Two destroyers heading in parallel directions

The results of the foregoing experiments would seem to indicate that the method of formulating designs was an advance on preceding work and that the efforts were conducted in the right direction. In general, the vanishing point system, built up with curves or straight lines, where they converge towards the bow is correct. Black may be used to block out superstructure or the design carried into the deck works in a convincing manner. The treatment of masts and stacks has been discussed previously and is understood. Any of the colors used in a given design may be almost as well used in any other. For example, design 2 might have used black, green and white instead of blue, gray, white and black; design 8 might as well have been painted with dark gray and pink rather than the blue and green.

The attempt to standardize the tests by using the same backgrounds with each model was done in order that any future work with the same apparatus might be compared with the results here given. Under like conditions the straight line plots measure the effectiveness of the design and may be compared with each other for relative merits. It will be noted that the majority of errors are between twenty and thirty degrees (that is the averages for the plots) so that the poorest of them would certainly cause sufficient error in a submarine observation to protect the ship against the torpedo attack. The errors would no doubt be accentuated with weather and haze aiding the ship.

An apparatus for neutralizing the effect of the camouflage might be devised. This machine as previously mentioned would probably consist of a series of revolving color screens which would enable the effect of all but black and white to be eliminated from the ship. Actually, the ship would assume a flat gray aspect. The time element in observations is very important and any equipment for discovering the camouflage would have to be adjusted and operated within a few seconds to be successful.

The day of camouflaging ships in wartime has passed with the passing of the submarine as an effective weapon of naval operations. It is unfortunate that so very little was accomplished during the progress of hostilities for the results might have prevented the loss of many ships, whereas camouflage had no more than a ten percent tendency towards safety.

**Appendix A: Results Tables**

| **Design 1** |  | | | | | | | | | |
| --- | --- | --- | --- | --- | --- | --- | --- | --- | --- | --- |
|  | **Obs 1** | | | **Obs 2** | | | **Obs 3** | | | **Average** |
|  | **Actual** | **Est.** | **Error** | **Actual** | **Est.** | **Error** | **Actual** | **Est.** | **Error** |  |
| **Clear** | 140 | 110 | 30 | 64 | 280 | 216 | 50 | 64 | 14 | **38.00** |
|  | 60 | 190 | 130 | 52 | 68 | 16 | 122 | 90 | 32 |  |
| **Hazy** | 135 | 122 | 13 | 54 | 44 | 10 | 52 | 120 | 68 | **53.50** |
|  | 58 | 110 | **42** | 128 | 56 | 72 | 130 | 240 | 110 |  |
| **Storm** | 112 | 62 | 50 | 128 | 72 | 56 | 135 | 42 | 93 | **73.00** |
|  | 75 | 60 | 15 | 58 | 72 | 14 | 52 | 50 | 2 |  |
| **Cliffs** | 112 | 66 | 46 | 46 | 110 | 64 | 51 | 52 | 1 | 38.00 |
|  | 112 | 66 | 46 | 126 | 66 | 60 | 126 | 136 | 10 |  |
|  | **Average angle of error:** | | | | | | | | | 50 |
|  | **Average angle of error assuming no error for white or black backgrounds:** | | | | | | | | | **36** |

Table A1. Results for three observer sets and their averages for Design 1. Values are in degrees. The error is the absolute difference between the actual direction of the model ship and the observer's setting of their perceived direction (two trials per condition). The average column is the average error for each skyscape condition. These are the results plotted in the results figures. The second line from bottom is the average of the entries above it. The bottom line is the average including assumed values of zero deg for nominal white and black backgrounds for which no data are reported. This is the dashed line in the results figures. The entries in bold denote calculation errors in the original report where > 1 deg.

| **Design 2** |  | | | | | | | | | |
| --- | --- | --- | --- | --- | --- | --- | --- | --- | --- | --- |
|  | **Obs 1** | | | **Obs 2** | | | **Obs 3** | | | **Average** |
|  | **Actual** | **Est.** | **Error** | **Actual** | **Est.** | **Error** | **Actual** | **Est.** | **Error** |  |
| **Clear** | 232 | 300 | 68 | 292 | 294 | 2 | 302 | 322 | 20 | **61** |
|  | 225 | 318 | 93 | **-** | **-** | **-** | 235 | 296 | 61 |  |
| **Hazy** | 300 | 244 | 56 | 296 | 230 | 66 | 300 | 130 | 170 | 73 |
|  | 238 | 246 | 8 | 230 | 290 | 60 | 230 | 310 | 80 |  |
| **Storm** | 314 | 298 | 16 | 234 | 292 | 58 | 302 | 286 | 16 | 53.6 |
|  | 250 | 290 | 40 | 312 | 300 | 12 | 242 | 62 | 180 |  |
| **Cliffs** | 292 | 300 | 8 | 242 | 280 | 38 | 240 | 124 | 116 | 34.5 |
|  | 242 | 258 | 16 | 308 | 286 | 22 | 303 | 310 | 7 |  |
|  | **Average angle of error:** | | | | | | | | | **54** |
|  | **Average angle of error assuming no error for white or black backgrounds:** | | | | | | | | | 37 |

Table A2. Results for three observer sets and their averages for Design 2. See caption to Table A1 for further details.

| **Design 3** |  | | | | | | | | | |
| --- | --- | --- | --- | --- | --- | --- | --- | --- | --- | --- |
|  | **Obs 1** | | | **Obs 2** | | | **Obs 3** | | | **Average** |
|  | **Actual** | **Est.** | **Error** | **Actual** | **Est.** | **Error** | **Actual** | **Est.** | **Error** |  |
| **Clear** | 46 | 126 | 80 | 57 | 56 | 1 | 60 | 120 | 60 | **38.4** |
|  | 49 | 52 | 3 | 118 | 70 | 48 | 130 | 130 | 0 |  |
| **Hazy** | 112 | 118 | 6 | 112 | 90 | 22 | 120 | 135 | 15 | **29.6** |
|  | 58 | 30 | 28 | 60 | 60 | 0 | 50 | 126 | 76 |  |
| **Storm** | 120 | 70 | 50 | 60 | 58 | 2 | 128 | 54 | 74 | 32 |
|  | 140 | 150 | 10 | 176 | 54 | **52** | 64 | 58 | 6 |  |
| **Cliffs** | 110 | 60 | 50 | 120 | 76 | 44 | 56 | 150 | 94 | 45 |
|  | 120 | 60 | 60 | 52 | 60 | **12** | 130 | 120 | 10 |  |
|  | **Average angle of error:** | | | | | | | | | 36.2 |
|  | **Average angle of error assuming no error for white or black backgrounds:** | | | | | | | | | 24 |

Table A3. Results for three observer sets and their averages for Design 3. See caption to Table A1 for further details.

| **Design 4** |  | | | | | | | | | |
| --- | --- | --- | --- | --- | --- | --- | --- | --- | --- | --- |
|  | **Obs 1** | | | **Obs 2** | | | **Obs 3** | | | **Average** |
|  | **Actual** | **Est.** | **Error** | **Actual** | **Est.** | **Error** | **Actual** | **Est.** | **Error** |  |
| **Clear** | 46 | 54 | 8 | 47 | 74 | 27 | 120 | 60 | 60 | 30 |
|  | 114 | 86 | 28 | 128 | 77 | 51 | 235 | 228 | 7 |  |
| **Hazy** | 54 | 118 | 64 | 126 | 62 | 64 | 50 | 64 | 14 | 40.6 |
|  | 116 | 124 | 8 | 128 | 112 | 16 | 128 | 50 | 78 |  |
| **Storm** | 120 | 110 | 10 | 44 | 60 | 16 | 118 | 60 | 58 | 21.6 |
|  | 42 | 45 | 3 | 117 | 80 | 37 | 54 | 48 | 6 |  |
| **Cliffs** | 120 | 62 | 58 | 46 | 56 | 10 | 128 | 54 | 74 | **29.6** |
|  | 78 | 76 | 2 | 122 | 80 | 42 | 35 | 148 | 113 |  |
|  | **Average angle of error:** | | | | | | | | | **35.5** |
|  | **Average angle of error assuming no error for white or black backgrounds:** | | | | | | | | | 20.3 |

Table A4. Results for three observer sets and their averages for Design 4. See caption to Table A1 for further details.

| **Design 5** |  | | | | | | | | | |
| --- | --- | --- | --- | --- | --- | --- | --- | --- | --- | --- |
|  | **Obs 1** | | | **Obs 2** | | | **Obs 3** | | | **Average** |
|  | **Actual** | **Est.** | **Error** | **Actual** | **Est.** | **Error** | **Actual** | **Est.** | **Error** |  |
| **Clear** | 125 | 112 | 13 | 128 | 114 | 14 | 125 | 68 | 57 | 28 |
|  | 46 | 38 | 8 | 126 | 120 | 6 | 126 | 54 | 72 |  |
| **Hazy** | 130 | 146 | 16 | 80 | 100 | 20 | 120 | 280 | 160 | 52 |
|  | 50 | 64 | 14 | 142 | 102 | 40 | 160 | 98 | 62 |  |
| **Storm** | 48 | 72 | 24 | 126 | 78 | 48 | 60 | 86 | 26 | 22 |
|  | 122 | 110 | 12 | 52 | 68 | 16 | 48 | 50 | 2 |  |
| **Cliffs** | 122 | 70 | 52 | 49 | 62 | 13 | 128 | 114 | 14 | **62.5** |
|  | 60 | 68 | 8 | 120 | 116 | 4 | 136 | 116 | 20 |  |
|  | **Average angle of error:** | | | | | | | | | **41.10** |
|  | **Average angle of error assuming no error for white or black backgrounds:** | | | | | | | | | **28.00** |

Table A5. Results for three observer sets and their averages for Design 5. See caption to Table A1 for further details.

| **Design 6** |  | | | | | | | | | |
| --- | --- | --- | --- | --- | --- | --- | --- | --- | --- | --- |
|  | **Obs 1** | | | **Obs 2** | | | **Obs 3** | | | **Average** |
|  | **Actual** | **Est.** | **Error** | **Actual** | **Est.** | **Error** | **Actual** | **Est.** | **Error** |  |
| **Clear** | 45 | 124 | 79 | 49 | 74 | 25 | 114 | 112 | 2 | **21** |
|  | 52 | 130 | **8** | 134 | 96 | 38 | 46 | 50 | 4 |  |
| **Hazy** | 138 | 42 | 96 | 64 | 100 | 36 | 122 | 128 | 6 | 47 |
|  | 130 | 70 | 60 | 136 | 130 | 6 | 42 | 120 | 78 |  |
| **Storm** | 122 | 66 | 56 | 38 | 72 | 34 | 131 | 60 | 71 | 35.3 |
|  | 46 | 34 | 12 | 32 | 60 | 28 | 309 | 300 | 9 |  |
| **Cliffs** | 60 | 42 | 18 | 41 | 66 | 25 | 123 | 92 | 31 | 33.3 |
|  | 42 | 120 | 78 | 134 | 106 | 28 | 72 | 52 | 20 |  |
|  | **Average angle of error:** | | | | | | | | | **38.00** |
|  | **Average angle of error assuming no error for white or black backgrounds:** | | | | | | | | | 22.70 |

Table A6. Results for three observer sets and their averages for Design 6. See caption to Table A1 for further details.

| **Design 7** |  | | | | | | | | | |
| --- | --- | --- | --- | --- | --- | --- | --- | --- | --- | --- |
|  | **Obs 1** | | | **Obs 2** | | | **Obs 3** | | | **Average** |
|  | **Actual** | **Est.** | **Error** | **Actual** | **Est.** | **Error** | **Actual** | **Est.** | **Error** |  |
| **Clear** | 230 | 252 | 22 | 232 | 260 | **52** | 303 | 302 | 1 | **17.5** |
|  | 216 | 220 | 4 | 307 | 294 | 13 | 130 | 150 | 20 |  |
| **Hazy** | 312 | 240 | 72 | 308 | 314 | 6 | 232 | 240 | 8 | 25 |
|  | 238 | 240 | 2 | 232 | 290 | 58 | 306 | 308 | 2 |  |
| **Storm** | 242 | 60 | **178** | 300 | 260 | 40 | 305 | 350 | 45 | **62** |
|  | 314 | 314 | 0 | 306 | 270 | 36 | 232 | 220 | 12 |  |
| **Cliffs** | 232 | 304 | 72 | 302 | 250 | 52 | 303 | 302 | 2 | **63** |
|  | 230 | 270 | 40 | 232 | 260 | 28 | 224 | 40 | **174** |  |
|  | **Average angle of error:** | | | | | | | | | **39.20** |
|  | **Average angle of error assuming no error for white or black backgrounds:** | | | | | | | | | 28.00 |

Table A7. Results for three observer sets and their averages for Design 7. See caption to Table A1 for further details.

| **Design 8** |  | | | | | | | | | |
| --- | --- | --- | --- | --- | --- | --- | --- | --- | --- | --- |
|  | **Obs 1** | | | **Obs 2** | | | **Obs 3** | | | **Average** |
|  | **Actual** | **Est.** | **Error** | **Actual** | **Est.** | **Error** | **Actual** | **Est.** | **Error** |  |
| **Clear** | 238 | 108 | 130 | 235 | 240 | 15 | 230 | 224 | 6 | 40.8 |
|  | 294 | 298 | 4 | 226 | 286 | 60 | 310 | 280 | 30 |  |
| **Hazy** | 230 | 248 | 18 | 242 | 282 | 40 | 310 | 300 | 10 | 29.6 |
|  | 230 | 312 | 82 | 224 | 242 | 18 | 238 | 242 | 4 |  |
| **Storm** | 230 | 310 | 80 | 301 | 292 | 9 | 305 | 250 | 55 | **56.4** |
|  | 130 | 130 | 0 | 232 | 250 | 18 | 128 | 238 | 110 |  |
| **Cliffs** | 226 | 338 | 112 | 240 | 264 | 24 | 313 | 310 | 3 | 34.5 |
|  | 280 | 250 | 30 | 298 | 280 | 18 | 233 | 252 | 19 |  |
|  | **Average angle of error:** | | | | | | | | | **36.8** |
|  | **Average angle of error assuming no error for white or black backgrounds:** | | | | | | | | | 27 |

Table A8. Results for three observer sets and their averages for Design 8. See caption to Table A1 for further details.

| **Design 9** |  | | | | | | | | | |
| --- | --- | --- | --- | --- | --- | --- | --- | --- | --- | --- |
|  | **Obs 1** | | | **Obs 2** | | | **Obs 3** | | | **Average** |
|  | **Actual** | **Est.** | **Error** | **Actual** | **Est.** | **Error** | **Actual** | **Est.** | **Error** |  |
| **Clear** | 222 | 30 | **168** | 294 | 284 | 10 | 236 | 232 | 4 | 37 |
|  | 236 | 240 | 4 | 238 | 247 | 9 | 248 | 222 | 26 |  |
| **Hazy** | 220 | 62 | **168** | 226 | 310 | 84 | 240 | 280 | 40 | 56 |
|  | 308 | 294 | 14 | 304 | 290 | 14 | 310 | 294 | 16 |  |
| **Storm** | 306 | 298 | 8 | 240 | 260 | 20 | 246 | 214 | **132** | 39 |
|  | 230 | 288 | 58 | 300 | 288 | 12 | 306 | 300 | 6 |  |
| **Cliffs** | 288 | 296 | 8 | 243 | 274 | 31 | 237 | 294 | 57 | 30 |
|  | 230 | 240 | 10 | 324 | 280 | 44 | 310 | 240 | **30** |  |
|  | **Average angle of error:** | | | | | | | | | **58.90** |
|  | **Average angle of error assuming no error for white or black backgrounds:** | | | | | | | | | 27.00 |

Table A9. Results for three observer sets and their averages for Design 9. See caption to Table A1 for further details.

| **Design 10** |  | | | | | | | | | |
| --- | --- | --- | --- | --- | --- | --- | --- | --- | --- | --- |
|  | **Obs 1** | | | **Obs 2** | | | **Obs 3** | | | **Average** |
|  | **Actual** | **Est.** | **Error** | **Actual** | **Est.** | **Error** | **Actual** | **Est.** | **Error** |  |
| **Clear** | 229 | 240 | 11 | 311 | 296 | 15 | 310 | 230 | 80 | 23 |
|  | 310 | 308 | 2 | 234 | 312 | **22** | 56 | 46 | 10 |  |
| **Hazy** | 220 | 220 | 0 | 308 | 260 | 48 | 300 | 302 | 2 | 32 |
|  | 324 | 224 | 100 | 218 | 240 | 22 | 223 | 242 | 19 |  |
| **Storm** | 304 | 312 | 8 | 308 | 300 | **2** | 306 | 90 | **16** | 30 |
|  | 226 | 226 | 0 | 230 | 294 | 64 | 141 | 50 | 91 |  |
| **Cliffs** | 228 | 308 | 80 | 312 | 297 | 15 | 250 | 110 | 140 | **37** |
|  | 306 | 294 | 12 | 231 | 254 | **77** | 320 | 320 | 0 |  |
|  | **Average angle of error:** | | | | | | | | | **38.90** |
|  | **Average angle of error assuming no error for white or black backgrounds:** | | | | | | | | | 20.00 |

Table A10. Results for three observer sets and their averages for Design 10. See caption to Table A1 for further details.

| **Design 11** |  | | | | | | | | | |
| --- | --- | --- | --- | --- | --- | --- | --- | --- | --- | --- |
|  | **Obs 1** | | | **Obs 2** | | | **Obs 3** | | | **Average** |
|  | **Actual** | **Est.** | **Error** | **Actual** | **Est.** | **Error** | **Actual** | **Est.** | **Error** |  |
| **Clear** | 123 | 62 | **59** | 130 | 96 | 34 | 128 | 52 | 76 | 41 |
|  | 115 | 58 | 57 | 52 | 56 | 4 | 312 | 296 | 16 |  |
| **Hazy** | 130 | 128 | 2 | 130 | 110 | 20 | 122 | 70 | 52 | **29.6** |
|  | 128 | 48 | 80 | 35 | 45 | 10 | 56 | 60 | 4 |  |
| **Storm** | 122 | 80 | 42 | 56 | 76 | 20 | 114 | 62 | 52 | **44.6** |
|  | 46 | 62 | 16 | 122 | 96 | 26 | 52 | 220 | **178** |  |
| **Cliffs** | 122 | 104 | 18 | 55 | 72 | **19** | 40 | 72 | 32 | **32** |
|  | 68 | 100 | 32 | 123 | 96 | 27 | 56 | 110 | 54 |  |
|  | **Average angle of error:** | | | | | | | | | **39.20** |
|  | **Average angle of error assuming no error for white or black backgrounds:** | | | | | | | | | **22.00** |

Table A11. Results for three observer sets and their averages for Design 11. See caption to Table A1 for further details.

| **Design 12** |  | | | | | | | | | |
| --- | --- | --- | --- | --- | --- | --- | --- | --- | --- | --- |
|  | **Obs 1** | | | **Obs 2** | | | **Obs 3** | | | **Average** |
|  | **Actual** | **Est.** | **Error** | **Actual** | **Est.** | **Error** | **Actual** | **Est.** | **Error** |  |
| **Clear** | 119 | 58 | 61 | 130 | 96 | 34 | 45 | 135 | 90 | **22.5** |
|  | 120 | 60 | 60 | 58 | 70 | 12 | 310 | 230 | 80 |  |
| **Hazy** | 130 | 58 | 72 | 120 | 62 | **68** | 48 | 54 | 6 | 29.6 |
|  | 60 | 50 | 10 | 68 | 90 | 22 | 310 | 310 | 0 |  |
| **Storm** | 38 | 52 | 14 | 54 | 66 | 12 | 50 | 310 | 100 | 44.6 |
|  | 98 | 128 | 30 | 122 | 94 | 28 | 50 | 134 | 84 |  |
| **Cliffs** | 38 | 50 | 12 | 47 | 44 | 3 | 121 | 80 | 41 | 32 |
|  | 122 | 110 | 12 | 126 | 102 | 24 | 42 | 142 | 100 |  |
|  | **Average angle of error:** | | | | | | | | | **40.50** |
|  | **Average angle of error assuming no error for white or black backgrounds:** | | | | | | | | | 22.00 |

Table A12. Results for three observer sets and their averages for Design 12. See caption to Table A1 for further details.
