# Supplementary material for: Blodgett's (1919) “Ship camouflage” 105 years on: A misperception of dazzle perception revealed and redressed
Source: Iperception. 2025 Mar 14;16(2):20416695241312316. doi: 10.1177/20416695241312316 (PMC11909666; doi:10.1177/20416695241312316)
Supplement: sj-docx-2-ipe-10.1177_20416695241312316 - Supplemental material for Blodgett's (1919) “Ship camouflage” 105 years on: A misperception of dazzle perception revealed and redressed [file sj-docx-2-ipe-10.1177_20416695241312316.docx]

**Edited version of Blodgett (1919)**

This document is a heavily edited version of Blodgett's (1919) MIT thesis (B.S.) on *Ship Camouflage*. The editing was done to improve gramma, tense, layout, organisation and headings, and general clarity of the original document. The work was done with due respect for the original author and for the benefit of the would-be readership. Every attempt has been made to preserve the original meaning but where reasonable doubt arose, this has been flagged with footnotes. No attempt has been made to check, corroborate, or correct historical or other factual details. The original document contains no reference section, and no references have been added here. American spelling has been changed to English spelling, consistent with the editor's nationality.

A pdf of the original work (written up on a mechanical typewriter) can be found [here](https://dspace.mit.edu/handle/1721.1/67156?show=full).

The citation to the original work is as follows:

Blodgett, L. S. (1919) "Ship camouflage," MIT thesis, B.S., Dept. of Naval Architecture and Marine Engineering. Department of Distinctive Collections, MIT Libraries, Cambridge, Massachusetts.

The original reformatting was done in February/June 2024 by Tim S. Meese (t.s.meese@aston.ac.uk; Aston University, UK).

The further editing (here) was done in May/June 2024 by Tim S. Meese (t.s.meese@aston.ac.uk; Aston University, UK).

This edited document and the earlier reformatted document are to be cross-referenced by Meese, & Strong (2025), *i-Perception*, (as Supplementary Materials, S1 & S2) who present a critical appraisal of Blodgett's work and a reanalysis of his results.

**Notes on numerical errors in Blodgett's thesis**

Several arithmetical errors were identified when transcribing Blodgett's results tables. No corrections have been made to the tables or graphical presentation, but mistakes (typically differencing or averaging errors) are highlighted in bold where > 1 deg. The magnitudes of the errors are not made explicit, but the interested reader will find sufficient information in the tabulated results to work out these details themselves. Where errors are noted in the reported averages, these are deviations from what should have been derived from the results as reported. No attempt has been made to correct numerical errors and the reported results/averages are consistent with the original document. A detailed reappraisal of the results is made in the document for which this one serves as supplementary material (S2).

*Ship Camouflage*

*Original report by Leo S. Blodgett N.A. Department - M.I.T. thesis, 1919*

*Edited by Tim S Meese, 2024*

**Abstract**

This undergraduate thesis (B.S., MIT, USA) follows work carried out during the Great War where contrasting colours and patterns were used in ship camouflage in attempts to distort enemy perceptions of their targets according to the 'British Dazzle' system. A quantitative/scientific treatment of the subject was conducted using: (i) information compiled in the USA and Europe during the war and (ii) equipment devised by the camouflage section of the United States Navy. An experiment was performed to test the perceived directions of scale model ships viewed through a periscope under a variety of conditions at simulated viewing distances of 1,100 yards and 2,200 yards with unlimited viewing times^[[1]](#footnote-1)^. The stimulus conditions included: class of ship (four types), camouflage colour and design (twelve types), background/skyscape (four types), seascape, weather, and true direction of the ship. Data were gathered from two naval experts and a group of four naive observers. The camouflaged ships produced errors in perceived direction between 17.5 deg and 73 deg with an overall average of 38.3 deg^[[2]](#footnote-2)^. By comparison to model ships without the application of dazzle camouflage (plain black or plain grey) where the errors were in the range of 2 deg to 8 deg, it is concluded that dazzle camouflage is effective in its job. While the subject remains of great interest—the work by the US Navy Department became usefully informed only during the last two months of hostilities—, it is unlikely to come into prominence again as a means of protecting ships against the submarine.

**Introduction**

Like many other methods of defensive and offensive warfare, marine camouflage has its origin in the early ages of recorded history. To appear less conspicuous to their enemies, the Greeks painted their ships blue, purple, green and vermillion for voyages of conquest. Mention is also made in the accounts of pirate ships during the fourteenth and fifteenth centuries where elaborate and conspicuous designs in purple, violet, green, white, and other colours, were painted on the sides of their vessels. However, during the latter part of the nineteenth century, the navies of the world painted their ships either entirely black or entirely white, both schemes having undesirable qualities in wartime. Germany recognised the need for a neutral tone and painted her ships blue-grey. Britain soon followed, followed by France, where a khaki-coloured grey was adopted. Around 1905, the United States chose a low visibility flat tone grey, still used on present battleships of the line.

After the outbreak of the Great War in 1914, the menace of the U-boats threatened to wipe out the transport and supply ships of the Allies and the question of a system of protective painting arose. As land camouflage developed and proved valuable in deceiving the enemy, Europeans and Americans attempted to develop similar methods for ships, with Mackay and Herzog of New York, seemingly the pioneers. By around June 1917, keen interest had arisen and in October 1917, the Treasury Department and the Bureau of War Risk Insurance issued an order that required all ships travelling under supervision to be painted in a protective manner for voyages across the danger zone. A penalty of increasing insurance by 0.5% was imposed when this instruction was not observed. The Submarine Defense Association, organized by shipping interests in New York, attempted to have all relevant ships painted with a government approved design. By way of developing these designs, the Eastman Kodak Laboratories at Rochester, New York were enlisted to carry out experiments on low visibility painting.

***The wild goose chase of the low visibility approach***

Extensive experiments were carried out by Lindell T. Bates and Loyd Jones of the Eastman Kodak Laboratories, who studied colours and combinations of colour that would blend to a flat tone at a given distance when applied in areas of specific proportions. Tests of grey painting on models were made on Lake Ontario and on the Atlantic, and a 125 ft patrol boat—the U.S.S. Gem—was assigned to the research team by the Navy Department. The team found^[[3]](#footnote-3)^ that at less than 5,000 yards, low visibility was beyond hope of attainment in clear weather. Although colours were found to blend at shorter distances, they did not give the desired tone. No flat tone pigment or paint of any description would absorb the incident light totally, and the reflective properties of a ship's surface, however slight, would destroy the desired effect. At 5,000 yards, a ship would fade into the skyline if painted grey or with colours that blended to grey, unless backlit by the sun, in which case, concealing the ship was beyond hope.

In their development of the low visibility idea, Mackay and others combined colours in precisely proportioned areas, obtaining a resultant grey, and recommended colours such as red, green, and violet, that superimposed^[[4]](#footnote-4)^ to give white. Bates and Jones determined that a shade of grey known as omega grey, was best used in the northern part of the danger zone where weather conditions were about seventy percent cloudy. South of Lat. 45 degrees North, a grey or bluer tone, called psi grey was found to be best. The combinations of colour to give the desired greys were alpha blue and beta white in equal parts for omega grey, and gamma blue and delta white for psi grey. However, the results of applying colours in mixed regions were found to be similar to the direct application of monotone shades, but since the monotone approach had proved ineffective (see above), the work done by this team was arguably in vain.

The death knell for the low visibility approach was that no ship, however well disguised, can remain invisible for long because of the remarkable hydrophone equipment carried on all submarines. With the apparatus in the hands of an experienced operator, it is possible to detect the presence of a ship at a range of ten or twelve miles and to determine her approximate direction. The attacking submarine would then approach her target until reaching a distance where direct observations could be made through the periscope.

***Test equipment development and the rise of dazzle***

Until March 1918, nearly all systems attempted to lower the visibility of ships at sea by painting them out of the skyline. While low visibility research was being conducted in the USA, a renowned artist, Lieutenant Norman Wilkinson of the Royal Navy Volunteer Reserve (R.N.V.R.), developed an entirely new system of camouflage in Great Britain, called 'British Dazzle', superseding all earlier ideas on the subject. Admiralty designs were immediately sent to Washington, and Wilkinson made a short visit to the Bureau of Construction and Repair to inform various constructors on what was known about the subject. Two Lieutenants, Jones and Van Buskirk, were designated as the organizers of a Department of Camouflage under the supervision of the Bureau. The Shipping Board appointed several individuals, many of them artists, to produce camouflage designs as might be used by the Navy Department, and to develop the subject using what means for research were available. From this, the Dazzle System was established as the basis of all designs and each of the various Camouflage Districts devised their own apparatus for studying the subject under simulated conditions of observations at sea.

Initially, the apparatus was rudimentary, consisting of a periscope with a minus lens and mirrors, and used to observe models placed in front of a background screen of painted skyscapes. Later, the Boston District devised a theatre of observation that was generally agreed to be superior to any other in the country. It included all the essential features to meet the illusion of sea and sky, lighting effects, and a periscopic method of observation comparable with real conditions. This is the apparatus used in the experimental work here and a description is given in the Methods section.

***Submarine attacks***

To understand the ideas behind camouflage, consider first the appearance of a ship as seen through a periscope, as well as the enemy's approach and their equipment. For torpedo and gunfire attacks, it is necessary to make accurate observations of the speed, range and course of the target ship. In the case of a torpedo attack, these observations are made through the periscope because the submarine is usually running below the surface. For gunfire, accuracy is determined by spot-firing on coming into range of the target. Periscopes are fitted with range finders, but estimates of range, course and speed are rather uncertain. To accommodate error, ten degrees in course, and two knots in speed are incorporated when plotting the position of the ship. An error of two thousand yards at a range of ten thousand (20%) has been noted in the log of one British submarine.

The German periscope expert, Dr. F. Weidert, expressed the difficulties of range finding from a submerged U-boat as follows:

Now it is already well known that correct estimations of range with the naked eye without some means of assistance is extremely difficult and is for many people actually impossible. With one-eyed vision through an optical instrument this is even more the case. On the other hand, precise knowledge of the range is essential for many purposes, especially for the firing of torpedoes. To unite with the periscope a range finder of sufficient base meets indeed with serious difficulties inasmuch as one cannot apply apparatus whose use will cause one to be prematurely seen by the opponent.

(Entwicklung und Konstruktion der Unterseebootensehrohr.)

To estimate range from a periscope observation, two methods are available. In the first, crosshairs divided into hundredths both vertically and horizontally in the eyepiece of the instrument can be used, or telemeter scales. The second, is the Goerz double image micrometre, which Lindell T. Bates describes in his report, 'The Science of Low Visibility':

Two pictures of the same object are made to cut each other in the lens, and are shifted with reference to one another until the tops of the masts, or other high parts, of a ship under observation in one picture are level with the water line in the other. The angle of shift is measured to determine the distance. This method, too. is only roughly approximate, for it has to deal with the measurement of a very small angle, and it assumes the height of the selected part of the vessel and uses this figure as length of base line. ^[[5]](#footnote-5)^

For all methods, when the ship is in motion, estimates of her length on the cross hairs would be approximate unless the observer is well-practiced. Estimates of a ship's course depends on the perceived alignment and perspective of masts, funnels, and superstructure. When ships take zig-zagging courses it is hard to make correct estimates, and if masts and structures are out of line, either by construction or by perspective painting, the difficulties are increased. Speed calculation depends on the triangulation method of observation, and this depends on the accuracy of range estimation. This means that if range is mis-judged, then the estimate of speed will also be in in error.

**Dazzle camouflage**

The greatest efforts have been made in investigating the systems of dazzle, originated by Lieut. Wilkinson. Any system of merit must distort the perception of the ship at a range of 1,000 yards or less to disrupt the accuracy of near observations. By painting the ship with bright or contrasting colours, outlines and structures are broken and, for a good design, the range, course, and speed of the vessel comes into doubt, prompting the submarine to make targeting corrections near the surface, remaining there longer than might be safe.

Her painting must be such that distortion is obtained under all conditions of weather and light. An error of 15 degrees^[[6]](#footnote-6)^ in perceived course from the combination of slight inaccuracies in the periscope and human error will be sufficient to disrupt a torpedo attack. If an error of 2 Knots in speed is also made, or of 200 to 300 yards in range, the submarine is effectively nullified as a torpedo menace to shipping^[[7]](#footnote-7)^. Shell fire, on the other hand, necessitates the submersible coming to the surface and resorting to spot-firing. In this case, the only defence of the ship under fire is her armament and the possible help of destroyers.

A surprising lack of high-quality information on the matter of dazzle camouflage is evident, and not until the last two months of hostilities was laboratory apparatus developed that could even approximate the conditions at sea. Nonetheless, several good principles were discovered, and all systems of effective disruptive painting must depend on these. However, with few exceptions, these principles have been completely ignored, the camoufleurs being given free rein to their imaginations and their results applied to ships regardless of merit.

It seems obvious in examining the matter that certain principles of distortion and perspective worked in a definite scheme^[[8]](#footnote-8)^, with colours of sufficient contrast accomplishing the desired results. If tests on such systems can be reduced to the best mathematical basis^[[9]](#footnote-9)^ that is feasible in the face of indeterminate matters due to sky and light conditions, the results would be of value.

**Motivation and approach for the current work**

As background to this work, practically all relevant data compiled on camouflage from the USA and from Europe were made available as an uncatalogued collection, but most was of little value in developing the subject. It was only during the last two months of hostilities that the matter was treated from a definite^[[10]](#footnote-10)^ standpoint by the Navy Department, with results starting to appear at the time the armistice was signed^[[11]](#footnote-11)^.

The experimental work here was based on principles of distorted perspective in combination with colours approved by the Navy Department which are not affected by colour filters to any appreciable extent^[[12]](#footnote-12)^. An effort was made to reduce the matter to a mathematical basis^[[13]](#footnote-13)^. With perspective designs, any concentration of light or dark colour will satisfy the principle that large masses of colour at bow, stern and superstructure, have a distorting effect. Remarkably, perspective designs were absent from the approved list of the Bureau until near the end of the war when several were distributed suggesting that the value of this approach had only then become apparent. In fact, towards the cessation of hostilities it became certain that the submarine was diminished as an effective weapon because of the perfection of the hydrophone and the use of depth charges. A submarine in motion and within the range of a listening device was surely doomed; the ferrets of chasers could find her and stay with her until an 'egg' was dropped when she might then come to the surface disabled and surrender or lie on the bottom and perish.

Despite this predicted demise of the U-boat, the subject holds a certain fascination. The work of this thesis strives to bring points of possible value to the fore should the matter once again come to prominence, as likely it never will. However, one possibility that might prompt its return is the use of sound and vibration deflectors by submarines as countermeasures to the hydrophone.

General proven principles of value in research have been adopted in this thesis, incorporated the aim of proving the value of certain basic principles of perspective painting in camouflage.

***An author's lament***

It must be remembered that the play of light is ever changing, toning down in places, increasing brilliancy in others, and affecting the perception of objects on the sea. To run a series of fieldwork observations on real painted ships under such conditions would be long, tedious, expensive and of doubtful accuracy, partly because few ships would be made available for such work. In trying to achieve reliable simulations of actual conditions on painted scenery and artificial light (see Methods below), one is convinced of the rather hopeless situation for the task at hand. However, the work of this thesis progressed nonetheless, using some limited knowledge of lights and shadows acquired at sea and along the New England coast, and applied to the apparatus described below.

***Experimental aim***

To cover the numerous environmental conditions in the theatre of submarine warfare, as many combinations of simulated sky, sea and light were used in the experiments as was reasonably possible^[[14]](#footnote-14)^. It was not practicable to use a moving model and so the experimental aim reduced to applying dazzle camouflage to distort the perception of a target ship's course. If demonstrated by experiment, we can assume that the enemy's understanding of range and speed will also be influenced^[[15]](#footnote-15)^.

Many atmospheric factors are involved for the ship at sea, and all of them, such as refraction and haze, contribute to the distortion of veridical perception. But since the tests under the artificial conditions here must be made as severe as possible, a distortion to perceived course of not less than 18 deg^[[16]](#footnote-16)^ must be achieved by the camouflage. If persistent errors are made greater than this, then the objective has been achieved, not least since the true atmospheric conditions at sea are likely to assist the ship yet further.

**Methods**

***Camouflage/stimulus design principles and application***

To provide a fair indication of the merits or failure of the following principles, the experimental observations were made in conditions that were as challenging as seemed plausible.

*Masts and superstructure*

From available data and reports, camouflage must accomplish the following. All high vertical points such as masts, stacks and superstructures must be distorted so that no estimate of course can be made by perceptual alignment. To achieve this, one mast alone should be used, as short as compatible with the efficient operation of the wireless. If two or more masts are strictly needed, they must be staggered rather than placing them on the centreline of the ship. To further help against the perceptual alignment of masts, corresponding parts should be painted in different colours. For example, when using black and white, the top of one should be black and the other white. With this arrangement, at no time will both be plainly visible, one of them blending with the background. This is particularly true of the colours to be discussed later^[[17]](#footnote-17)^.

*Stacks and superstructure*

The stacks must be short and, if necessary, forced draft should be used to achieve the desired result. False superstructures can be used but these require rigid construction to withstand the weather, making them cumbersome, and they are expensive to construct. There are two methods to conceal superstructure and false works. There is little to choose between them, but one offers simpler application than the other. The first is to extend the distortion or perspective painting consistently from the hull to the superstructure. The second requires the entire structure including the forward and aft vertical surfaces and the lower part of the stack(s) to be painted in a dark monotone shade (e.g., black or violet). The result is quite remarkable, in that it becomes almost impossible to determine angles or lengths^[[18]](#footnote-18)^. The top of the stack should be painted with the lighter tones used on the hull.

*Hull*

At the time hostilities ceased, the Bureau supposed the hull to consist of three main divisions: the bow, the midships and the stern, and proposed treating them as follows. The midships was devoted to breaking of outlines, whereas for the bow, the intention was to turn it perceptually away from the observer using light, pale colours, in small divisions. Furthermore, instead of trying to imitate the stern on the bow, as had often been the case, the stern was to be brought forward to the quarter. Painted shapes and patterns within these three divisions were to be designed with three things in mind:

i) There should be a continuous perspective design through the three spaces.

ii) The important forms should be constructed with large and small ends, with the small end set forward.

ii) Where possible, that all important lines point downward from the stern to the bow.

This last point might be questioned in the light of the experiment here and will be reconsidered later^[[19]](#footnote-19)^.

The camouflage designs for the model ships here used the ideas above and the following constraints. The breaking of outline by painting is necessary but it is not essential to blotch colours on the side of the ship in contrasting masses in an artistically hit or miss way. Furthermore, any desired effect may be combined with the application of forced perspective. Any colour of any desired intensity may be applied to any part of the ship but only as part of the scheme being aimed for. Lines may be smooth but not monotonous so that if a line or painted strip is followed by the eye it may not be possible to determine direction, particularly if given a turning movement^[[20]](#footnote-20)^. The same curve that appears concave in one moment might seem to be convex with a slight change of light. When contrasting colours are arranged in any good perspective design it is practically impossible to determine the ship's course accurately.

Twenty-four designs were used in this work, all of them employing a forced perspective or converging line principle. No two were in any way similar and, to be of value, might be coloured with practically any one of the combinations to be discussed below. The twelve best^[[21]](#footnote-21)^ designs (Figures 3 & 4) were chosen for presentation and discussion here.

*Colours*

In choosing colours it was safe to discard brilliant ones. Of the original twenty flat tone pigments selected, six were discarded following testing, leaving the following fourteen, presented here with their standard Navy Camouflage designations: Black, White, Grey-white, No. 4 Grey, No. 1 Grey, Grey-pink, No. 3 Pink, No. 1 Grey-green, No. 1 Green, No. 1 Blue, No. 3 Blue, Lavender, Violet, Yellow (see Figure 1)^[[22]](#footnote-22)^.


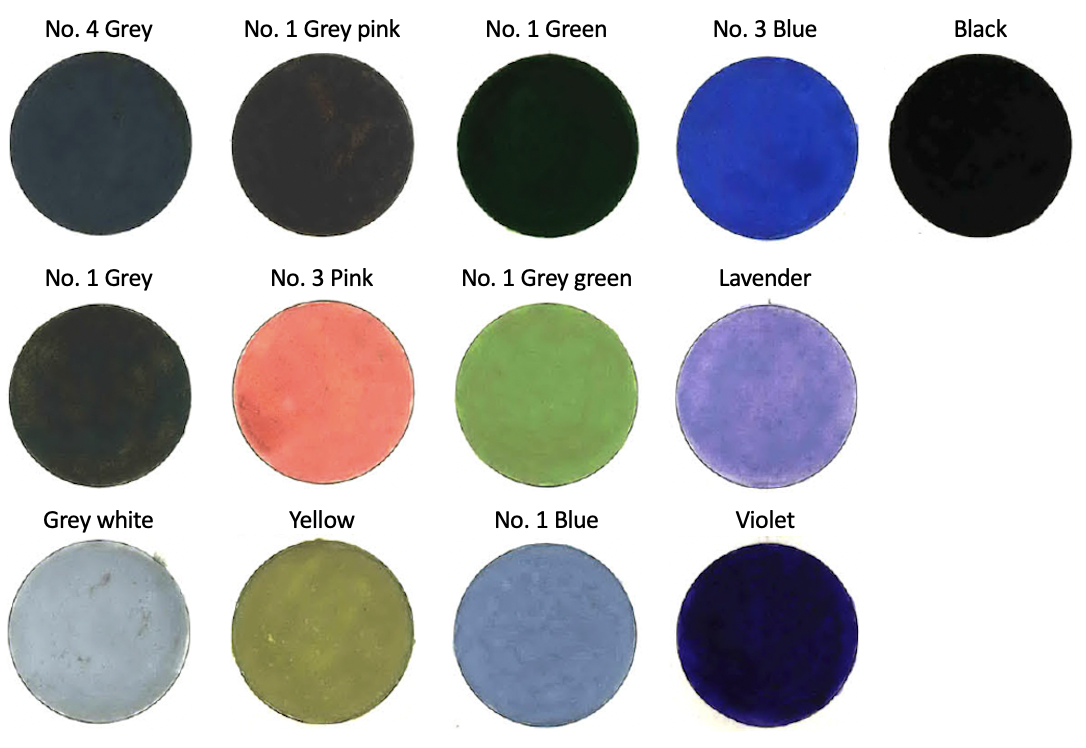


Figure 1. The fourteen colours (those shown plus white) used for the ship camouflages in the experiment.

Any intensity or tone within this range was possible, but none of the designs used tones other than those in Figure 1^[[23]](#footnote-23)^. All decks were painted with No. 1 grey. The various combinations of these colours as applied to the model ships are shown in the Results section.

Designs were as simple as possible with the idea of applying them to the side of a vessel^[[24]](#footnote-24)^ and as few colours as possible were used in each design to achieve the object of contrast.

Attempts at low visibility dazzle seem rather impossible here, but one or two cases were tried (Designs 5 & 6; see below) and were found to be of possible value.

*Ship classes*

The designs were applied to four different classes of model ships: (i) tramp/cargo ships with superstructure at the stern, (ii) tramp/cargo ships with superstructure amidships, (iii) passenger ships, and (iv) destroyers.

***Equipment, experimental conditions, and procedure***

The equipment used in the experiments reported here belongs to the camouflage test facility of the Boston Camouflage District (BCD)^[[25]](#footnote-25)^.

*Water and sky*

The simulation of water was achieved^[[26]](#footnote-26)^ by building up a curved painted surface to mimic the appearance of the sea when observed from a submarine. An endless belt of canvas painted with various seascapes was run over rollers^[[27]](#footnote-27)^ to simulate a variety of weather conditions (see Figure 2). Various sky conditions and shorelines were obtained in a similar way to the water but in the vertical plane. Hand cranks were used to operate both canvas belts. The bed over which the sea canvas rolled was given a downward turn to achieve a true representation of water in the horizontal plane and the visual centre of the periscope was brought low enough to the horizon to approximate the views seen from a real submarine.


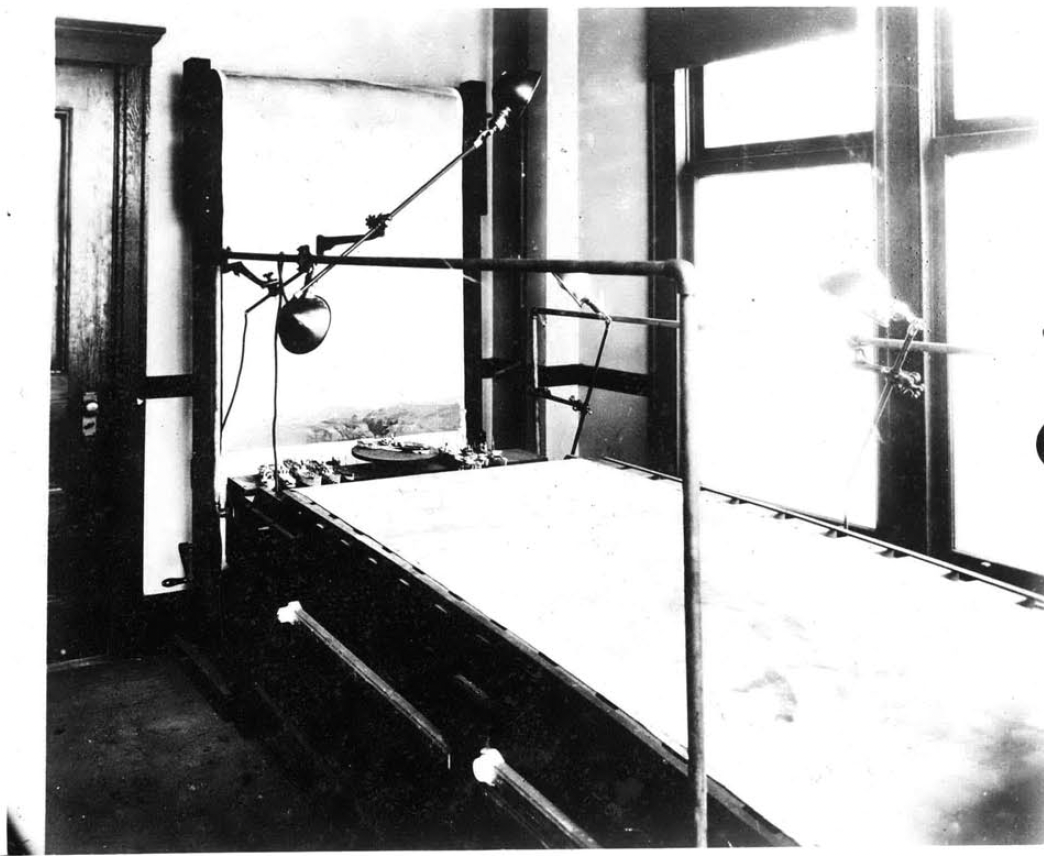

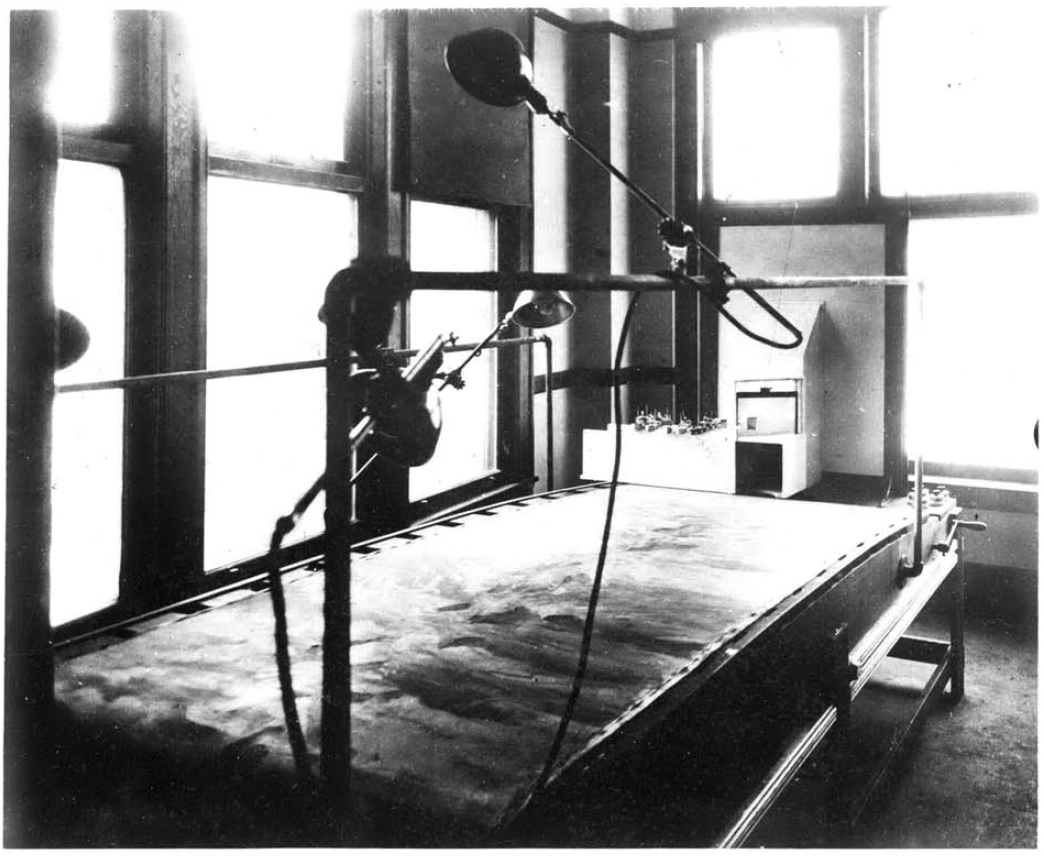


Figure 2. Two views of the equipment of the Boston Camouflage District (BCD) used in the experiment. Note the horizontal belt used to simulate sea and in the view on the left, the vertical belt used to role one of four different skyscapes into place. Note also the hand crank in the view on the right, towards the rear of the belt.

*Periscope, scale, and fog*

It was not possible to follow the true periscope exactly because for the scale models in the experiment it was necessary to produce a virtual image reduced in size, whereas the true periscope would produce a real image enlarged in size^[[28]](#footnote-28)^. The models were bult to a scale (1/32":12") which allowed true representations of the ships viewed through the periscope. Two mirrors and two lenses comprised the working parts of the periscope, the mirrors being fixed parallel to each other and at a forty-five-degree angle with the vertical, and the lenses placed between the mirrors. The lower lens was fixed and had a diameter of four inches with a focal length of thirty inches, while the upper lens was a three-inch minus or reducing lens which allowed the image to be varied in size as required.

A fog producing device was installed directly in front of the bottom opening of the periscope. It consisted of a semi-transparent mirror placed vertically at a forty-five-degree angle to the line of vision. A ground glass screen was placed in the vertical plane parallel to the line of vision and fog densities could be varied by adjusting a light enclosed near to this mirror. Blue and ground glass screens were used to give the correct tone for daylight fog. Horizontal light carriers were erected along the sides of the frame, and nitrogen daylight lamps were used to produce lighting effects on the sea and sky^[[29]](#footnote-29)^.

*Psychophysical task: Direction matching of the model ships*

A turntable was installed between the horizontal edge of the sea and sky scenery. On turning this with a hand wheel, a pointer on a compass card showed the physical direction of the ship on the turntable. This card was located near the eyepiece of the periscope so that as perceptual estimates of direction were made and shown by another dial, the two could be compared conveniently^[[30]](#footnote-30)^ to determine the magnitudes of perceptual errors. The directions and errors were recorded manually in pre-prepared tables.

*Experimental environments*

The apparatus had four distinct skyscapes:

(i) Clear blue sky.

(ii) Hazy, dull sky, in which cumulus clouds predominate.

(iii) Dark storm clouds, touched with colour including tints of orange, yellow, and patches of blue.

(iv) Ragged shorelines typical of the French and English coasts.

The seascapes had the effect of toning the sky and reflecting light on the ships and four types were painted on the horizontal canvas belt:

(i) Calm blue, summer sea.

(ii) Bright green water, slightly ruffled.

(ii) Dull grey hazy sea.

(iv) Rough white capped choppy weather.

It is difficult to determine which or how many seascapes might be used with each skyscape to appear real. Table 1 shows the sea effects used in the experiment for each sky and illustrates the difficulty of the matter.

| Sky | Seascape |
| --- | --- |
| Clear blue sky  It is very important to consider the angle from which the sun strikes the ship | On a calm day with a slight haze, the sea might be blue; then with hard metallic sunlight, a green sea might result; and on a clear, windy fall day, a choppy blue sea would occur. |
| Hazy sky | All four effects of sea might occur if the lighting were properly regulated. |
| Storm sky | Either the dull, flat sea if the wind had not ruffled it, or if the squally or puffy winds were blowing, the dark rough chop would result. |
| Shoreline | Practically any one of the four might occur with proper lighting. |

Table 1. The seascapes used for each skyscape in the experiment.

*Shortcomings of the equipment*

The apparatus described above provided the best approach available for the effective simulation of conditions at sea. However, there were some shortcomings. First, no scientific study of the apparatus was made (e.g., calibration^[[31]](#footnote-31)^). Second, the curve of the sea belt, the arrangement of the periscope, the lighting, and the painting of the sea and sky, were all chosen to appear natural to artists familiar with sea painting, and a little too much was left to their artistic temperament and imagination. Nonetheless, by testing models in a way that leaves as little as possible to the imagination of the observer, good comparative results can be obtained with this equipment.

***Participants***

The first observer was a Lieutenant in one of the European Navies, entirely familiar with ships, periscopes, range finders, and the conditions at sea under which observations occur. Before taking part in the experiment, he became familiar with each design, meaning that any errors in his settings were notwithstanding this knowledge. The idea here was that repeated observations on a design without a time limit would cater for the possibility that submarine commanders might become familiar with specific camouflage designs.

The second observer was very closely in touch with the evolution and application of each camouflage design, making repeated observations on them, and offering valuable suggestions during the progress of the work.

The third set of observations was made by four participants. None was familiar with the camouflage designs prior to the experiment and were naive in this respect. However, all were familiar with the structures of the ships, the principles of perspective and visual illusions, and the conditions of light and shadow at sea.

*Simulated distances*

To stress test the camouflage designs, the simulated distances at which observations were made were severe, being somewhat less than in most real attacks at sea. A range of 1,100 yards was used for four participants and 2,200 yards for the other two^[[32]](#footnote-32)^.

***Experimental design***

The perceived directions for each of two physical directions and for each of twelve camouflage designs under the four different conditions of sky (Table 2) were measured for each of the six observers (above) with sea and lighting conditions varying across them (2x12x4 = 96 observations per participant). The aim was to have no two scenes alike so that variations in the lighting or the sea would influence the shadows, tending to alter the perceived aspect of the ship.

Although usually understood to apply to direct mathematical measurements, a study of the methods of the precision of measurements^[[33]](#footnote-33)^ can also be applied to indirect measurements, and the case in hand is comparable to certain calculations entailed in chemical combinations where precision has been applied.

**Results**

Absolute (i.e., unsigned) errors for each ship were averaged for each observer and then averaged across all observers. (The raw data and averages are reported in the twelve tables of Appendix A^[[34]](#footnote-34)^). Figures 4 & 6 (for designs 1-6, and designs 7-12, respectively) show the average errors (ordinate) for each of the four main skyscapes (abscissa) in the graduated order of: clear, hazy, storm, cliffs. These are flanked by white and black backgrounds/conditions (not mentioned in the Methods section). For the white condition, the error was assumed to be zero and this was borne out by experiment^[[35]](#footnote-35)^. For the black condition, the error was assumed to be zero^[[36]](#footnote-36)^.

The areas under the (jagged) black results curves in Figures 4 & 6 are quantitative measures of the effectiveness of the camouflage designs^[[37]](#footnote-37)^, and by comparing them across the twelve plots the relative merits of the designs can be seen. The results for monotone black and grey ships^[[38]](#footnote-38)^ (Table 2; not mentioned in the Methods section) appear at the bottom of each plot (blue and red curves, respectively) so that the application of camouflage can be compared with its absence.

The broken black horizontal lines in Figures 4 & 6 show the average under all conditions of weather^[[39]](#footnote-39)^.

|  | **Black** | **Grey** |
| --- | --- | --- |
| **Clear** | 2 | 2 |
| **Hazy** | 8 | 4 |
| **Storm** | 5 | 3 |
| **Cliffs** | 3 | 7 |

Table 2. Perceptual errors (in degrees) for Black and Grey ships (different columns) as they would be painted in normal times for each of the four skyscapes. These are plotted as thin blue and red lines in results Figures 4 & 6.

***Design 1***

This design (Figure 3, top-left) was the simplest possible application of perspective. All boundaries converge at a point either explicitly, as the arrowhead at the bow, or as the bounding curves of the other pattern elements^[[40]](#footnote-40)^. The masts were treated so that the grey and the pink divisions would not stand out from a background simultaneously. The grey patches from the hull were extended to the stack and superstructure. The choice of the grey and pink might seem rather poor at first glance, but they produced marked contrast and proved to be most^[[41]](#footnote-41)^ effective in the tests.

The photographs of the various models (insets to Figures 3 & 5) give some idea of the ship as observed through the periscope. It was impossible to focus the camera through the periscope lens, so the pictures were taken from a slight elevation. This meant the camera lens could not be brought close enough to the water level to fully eliminate the sea from the background. Because of this, the directions of the ships can be estimated much more readily than when appearing against the sky as they did in the experiments.

The photographic reproductions (insets to Figures 3 & 5) illustrate the maximum effect that can be achieved using colour filters^[[42]](#footnote-42)^. To approximate this effect in actual observations, it is necessary to devise a system of filters that in a very short interval of time will eliminate the red and green tones from the ship and cause it to approach a flat painted surface in appearance^[[43]](#footnote-43)^. Observations may not be prolonged more than thirty or forty seconds^[[44]](#footnote-44)^.

For this design (inset of Figure 3, top-left) the single mast^[[45]](#footnote-45)^ is quite effectively blocked out of the skyline, with the exception of the very top, which would not be useful in determining the size or course of the ship. The design is simple, but the actual effect of the colours is necessary for perceptual distortion. The ship is heading 30 deg away from the observer.

Figure 3. Designs 1-6 (out of 12) used in the experiments. Designs 1-3 are applied to tramp steamers with superstructures and stacks placed aft. For Designs 4-6, the superstructure and stacks are amidships.


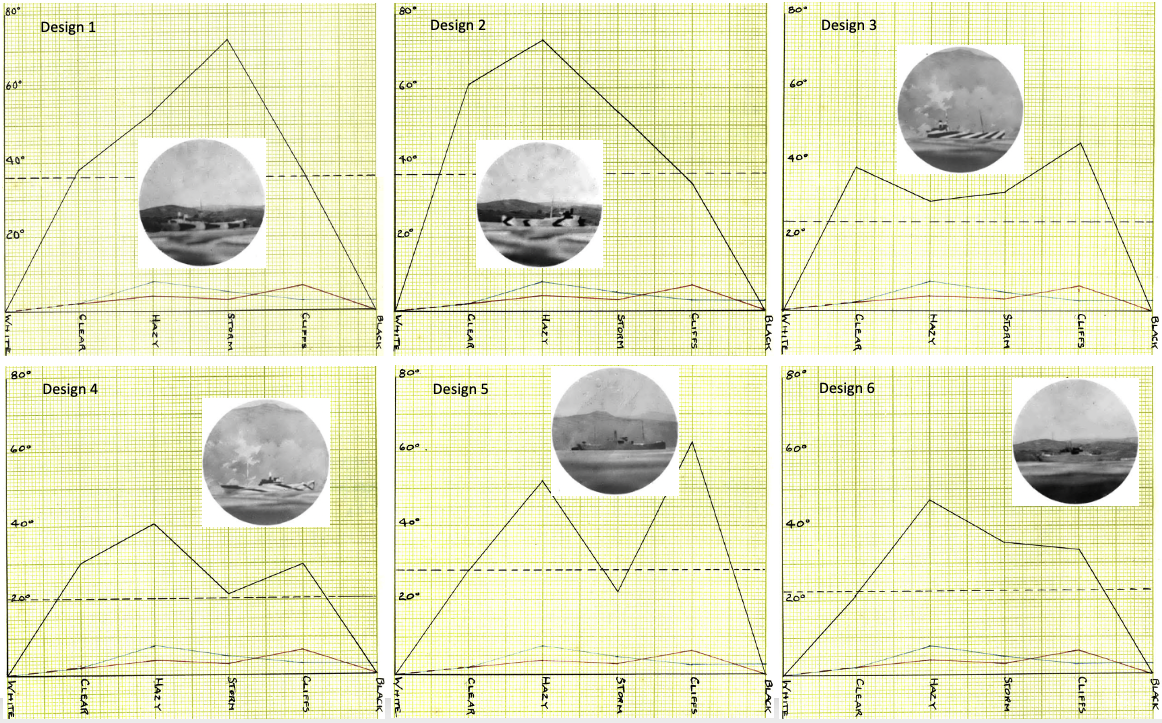


Figure 4. Results for Designs 1-6. The ordinate gives the average error from Tables A1-A6 in Appendix A for six backgrounds which are from left to right: White, Clear, Hazy, Storm, Cliffs, Black. The errors were assumed to be zero for the black and white backgrounds, but no data are reported. The dashed line is the average across background conditions including black and white. The blue and red lines are for black and grey ships, respectively (see Table 2). The insets indicate views similar to that seen through the periscope (see text for details). The ship directions in the insets are: D1: 30 deg away; D2: 30 deg towards; D3: 30 deg towards; D4: 20 deg astern; D5: 25 deg towards; D6: 20 deg towards.

***Design 2***

It was determined that black, blue and white had the maximum contrast and these colours came to be used in several designs. In this case (Figure 3, top-middle) they were applied using the converging line principle, but in such a way that the points of each coloured region do not align, and the eye does not follow directly between them. It is difficult to determine the shape or size of the superstructure when painted black, and here the broad stripe of black carried from the hull to the superstructure masks the outlines. No. 2 grey-white was incorporated with the idea that against certain skies and weather conditions, the sections so painted would be lessened in visibility in the same proportion that the contrasting parts would attract the eye, thus breaking the outlines further.

From the inset for Design 2 in Figure 4 (top-middle) it might be assumed that the ship was approaching the observer at an angle of maybe ten degrees from the horizontal. In fact, it is 30 degrees.

***Design 3***

In this design (Figure 3, top-right), all lines on the hull converge at a point forward of the ship and a little above it. This vanishing point system is most effective in obtaining a perceptual turning movement of the ship. The narrower lighter stripes at the bow also tend to turn the ship away from the observer, while the heavier striped stern brings it into prominence and towards the observer. Black is used to paint out the superstructure, and the masts are painted in the accepted manner. No. 1. green was used for the sake of variety and to test its value, but No. 3 blue might have been used instead^[[46]](#footnote-46)^. It is possible to screen out the green, but the resulting black will serve the purpose of distortion nearly as well.

From the inset for Design 3 in Figure 4 (top-right), the ship would seem to be headed away from the observer, but it is coming ahead at an angle of 30 degrees. The masts are out of line, which helps the distortion. The design illustrates the effect of lines converging at a vanishing point very well, and that even a simple design can produce excellent results.

***Design 4***

The first three designs were painted on tramp steamers with superstructures and stacks placed aft. This made the distortion harder to achieve than when superstructures were placed amidships as in the ship of Figure 3, bottom-left. Here, two vanishing points were used, one above the bow and forward of the ship, the other below. It was intended that this would perceptually turn the bow and stern away and towards the observer, respectively. The combination of black, No. 3 blue, and white is good in all designs: the black and white for contrast, and the blue because it is difficult for the eye to focus on. The design is carried from the hull to the superstructure.

In the inset to Figure 4, bottom left, the double vanishing points turn the ship perceptually away from the observer more than its true physically direction of 20 degrees astern, which might be anything up to 45 degrees.

***Design 5***

Low visibility was discussed in the Introduction, and it was decided that under a range of 5,000 yards this approach was not effective. Design 5 (Figure 3, bottom-middle) was derived with the idea of trying to achieve dazzle by using colours but with comparative low visibility. Beyond 3,000 yards, however, the colours blend and, while nearly invisible against some backgrounds, the design inherits the general faults of monotone painting. The pattern in this design involves the arcs of circles, and if other colours had been used to give contrast, it would have been effective at all ranges^[[47]](#footnote-47)^. However, the interest here lies in its distortion at a range of 3,000 yards^[[48]](#footnote-48)^. Camouflage of the superstructure is extended from the hull and the masts are properly treated.

In the inset to Figure 4, bottom-middle, the grey and green blend to a flat tone on the plate and quite effectively alter the course. This grey is the result of using two colours of marked contrast yet even with the naked eye at a range of about 1,500 yards, the colours tend to blend. The true direction here is 25 degrees towards the observer.

***Design 6***

In this design (Figure 3, bottom-right), contrast is again^[[49]](#footnote-49)^ obtained using colours that were not considered in the research work during the war. The design is one of reverse perspective with violet applied in broad stripes at the stern and, on being carried to the superstructure, is as effective as black would be under the same conditions. It is a very simple design, easily applied to the side of a ship, and breaks up the ship's outlines effectively.

In the inset to Figure 4, bottom-right, as for Design 5, the grey results from a combination of contrasting colours, in this case, dark purple and bright green. Both examples of low visibility (Design 5 and Design 6) show the good qualities of this method in obscuring outlines. However, maximum effects are obtained using colours of stronger contrast.

The physical direction of the ship in Figure 4, bottom right, is 20 degrees towards the observer.

***Design 7***

Mackay determined that red, green and lavender would result in a grey tone with warm hue when applied in small areas thereby achieving a low visibility effect. In contradistinction, these colours were applied to the passenger ship type used here (Figure 5, top-left) in sufficiently large areas to produce a splendid dazzling effect for the observer. All lines converge at a vanishing point near the bow. The lavender attracts the eye less than the brighter colours of green and pink and has the effect of masking the outlines. The curve at the bow tends to twist it away from the observer perceptually.

This combination of curves and vanishing points impose a perceptual turn on the ship in natural colours, while in the inset to Figure 6, top-left^[[50]](#footnote-50)^, the camera has almost neutralized the effect where the physical direction is 30 degrees towards the observer.

***Design 8***

No. 3 blue and light green used in Design 8 (Figure 5, top-centre) do not have the strength of contrast of some other combinations, but as used in this type of design—which might be described as a herring bone perspective—, they give rather good results. The two sections of the design converge at a point near the bow, and this seems to break the bow apart from the ship. Under certain lighting, it was found that this appears to turn the ship away from the periscope, while for other lighting, the ship appeared to turn towards it. The superstructure is effectively distorted by extending the design to the hull. The rather large masses of blue at the stern tend to interfere with the perceived length of the ship.

The pattern has been decidedly altered on the negative (inset, Figure 5 top-middle) where the light parts of the image are a medium blue on the ship, and the dark parts are light green. Nonetheless, the contrast is approximately preserved. The true direction of the ship is 30 degrees towards the observer.

***Design 9***

This design involves a combination of black, No. 3. blue and white in large regular curves and is effective on this type of passenger ship (Figure 5, top-right). All curves work towards the point at the bow and twist the bow away from the observer perceptually.

This is a very good example of the twisting effect of long curves converging at a point either on the hull or at a vanishing point outside the ship's boundary. In the inset to Figure 6, top-right, the physical direction is 30 degrees towards the observer.


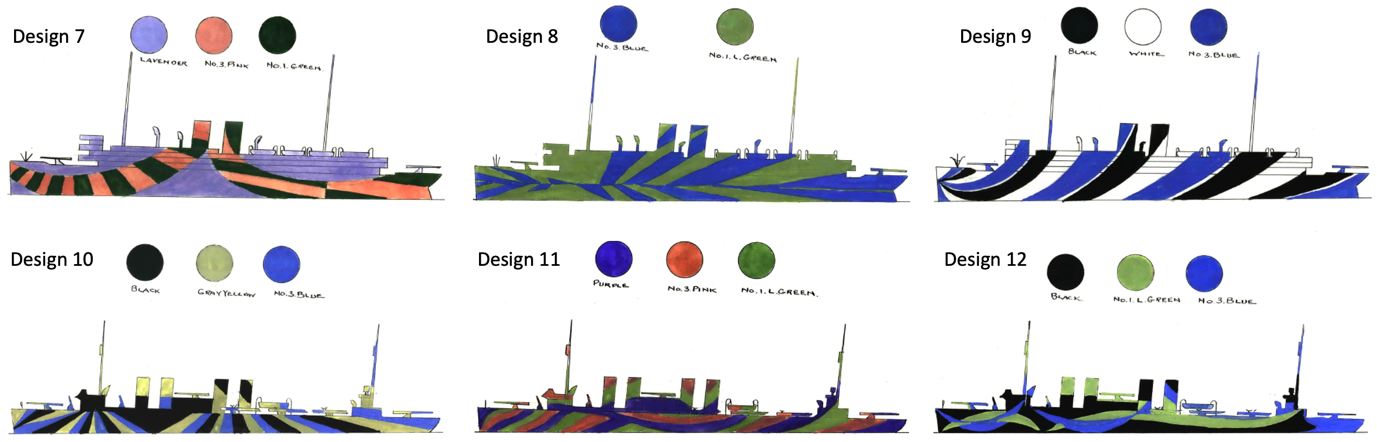


Figure 5. Designs 7-12 (out of 12) used in the experiments. Designs 7-9 are applied to passenger ships and designs 10-12 are applied to destroyers.


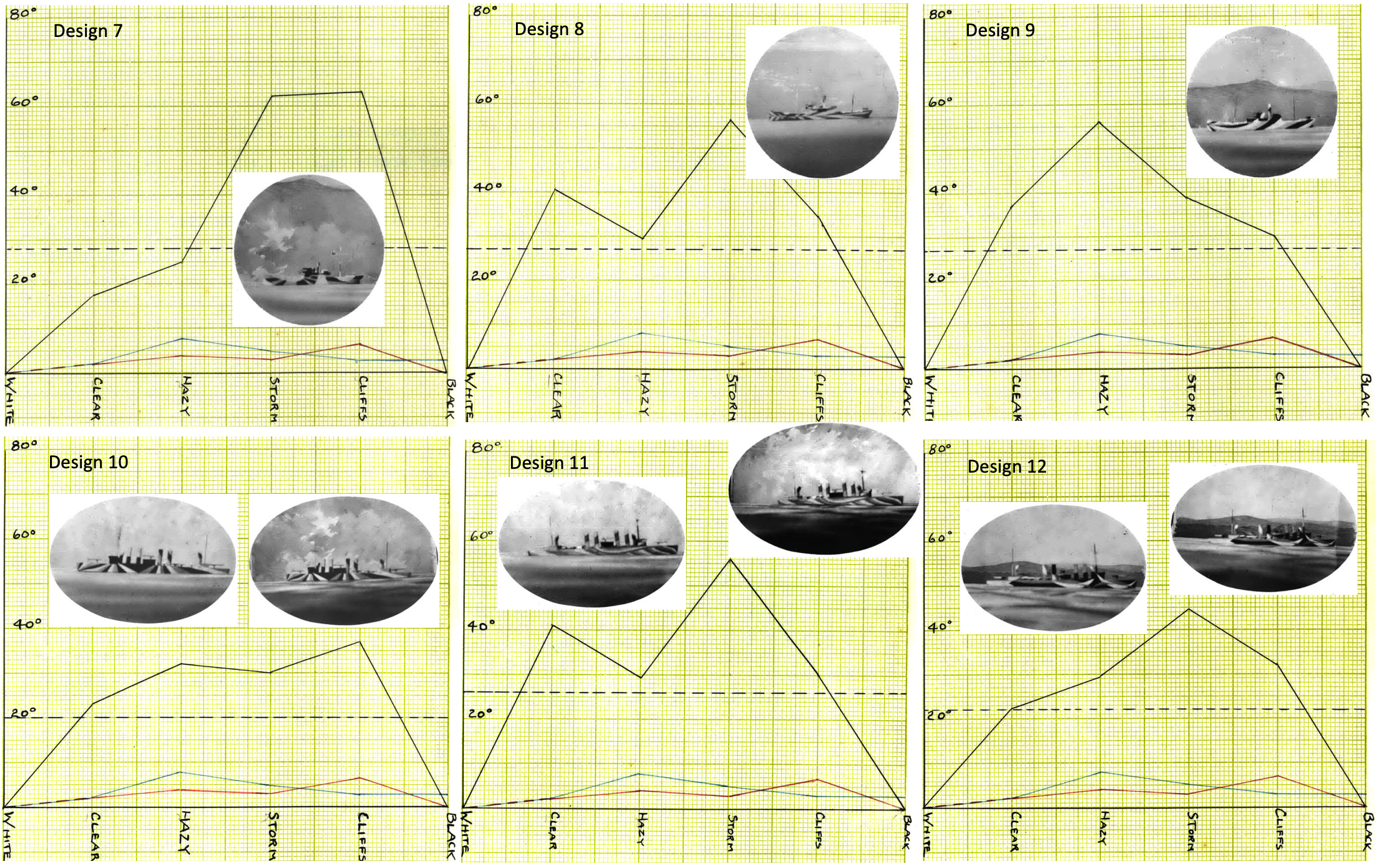


Figure 6. Results for Designs 7-12. The data are from Tables A7-A12 in Appendix A. See caption to Figure 4 for details. The ship directions in the insets are: D7: 30 deg towards; D8: 30 deg towards; D9: 30 deg towards; D10: 25 deg astern; D11: 20 deg towards; D12: 30 deg astern.

***Design 10***

Destroyers are rather difficult to camouflage successfully because of the small amount of freeboard and the massive superstructure and stacks, all of which have a decided rake aft (Figure 5, bottom-left). This first destroyer design employed extensive areas of black to distort the structure. It is nearly impossible to determine the type of ship or direction of travel using this design, no outlines being sufficiently clear to determine the course. It is a rather successful combination of black, No. 3 blue, and yellow.

It has been noted that it is difficult to disguise a destroyer or to alter her superstructure, but these final three designs exemplify the possibilities of distortive painting in this respect. The perceptual effects illustrated by the insets on the bottom row of Figure 6 were more marked when seen in colour. The physical direction in the insert of Figure 6, bottom-left, is 25 degrees astern.

***Design 11***

This is another case of pink, green and lavender, applied to achieve dazzle (Figure 5, bottom-middle). This design tends to lower the visibility of the ship under certain lighting, even at ranges of 1,100 yards. While this reduction in visibility might be of little value^[[51]](#footnote-51)^, the benefits of perceptual distortion make the design acceptable, the outlines being rather well broken.

The photograph of this painting was expected to reveal low visibility (inset, Figure 6, bottom-middle) and against certain backgrounds visibility was reduced. The results are passable but not as good as some others. The physical direction here is 20 degrees towards the observer.

***Design 12***

The lavish use of black once again helps to break up the form of the superstructure, while the long regular curves in contrasting colours give the bow a decided perceptual distortion (Figure 5, bottom-right). The No. 3. blue stern interferes with the perceived length of the ship while the mass of black on the quarter attracts attention and serves to shorten estimates of length even further.

This painting indicates how effectively the ship can be perceptually distorted and the superstructure broken by using black and medium blue. It is difficult to determine the length of the ship either in the photograph (inset, Figure 6, bottom-right) or in observing the model through the periscope. It is equally hard to decide on the number of stacks and the disposition of deck works. The physical direction of the ship here is 30 degrees astern.

Other colour schemes for the designs considered here are discussed in the next section.

**Discussion and Conclusion**

Figure 7 was included by way of illustrating the twist effect. The ships were placed about

six inches apart and as nearly parallel as possible. The results of the camouflage are self-evident.


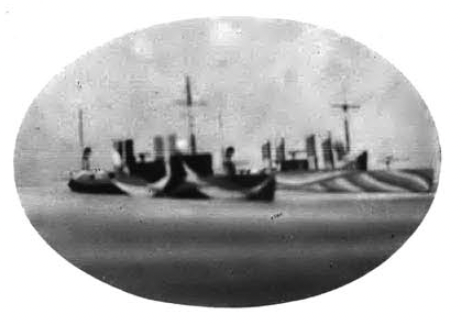


Figure 7. Two destroyers heading in parallel directions.

The results of the experiments here suggest that the method of formulating dazzle designs towards the end of the war was an advance on earlier work and that efforts were being conducted in the right direction. In general, the vanishing point system using curves or straight lines to converge towards the bow is correct. Black may be used to mask superstructure, or the design carried into the deck works in a consistent manner. The treatment of masts and stacks has been discussed previously and is understood. Any of the colours used in a particular design may be used almost as well in any other. For example, Design 2 might have used black, green and white instead of blue, grey, white and black, and Design 8 might have been painted with dark grey and pink rather than blue and green^[[52]](#footnote-52)^.

The attempt to standardise the experimental tests by using the same backgrounds for each model was done so that future work with the same apparatus might be compared with the results reported here. Under comparable conditions, Figures 4 & 6 show the effectiveness of each design and can be compared across designs for their relative merits. It is noted that most of the errors are between twenty and thirty degrees (the plot averages), higher than the benchmark of 18 degrees, so even the poorest of the designs would induce sufficient error in a submarine observation to protect the ship against torpedo attacks. These errors would no doubt be amplified when weather and haze aided the ship.

An apparatus for neutralizing the effect of the camouflage might be devised in the future. This machine, as previously mentioned, might consist of a series of revolving colour screens that would eliminate all but the effects of black and white from the ship. In fact, the ship would assume a flat grey aspect. However, the time element of observations is important and any countermeasure equipment for camouflage would have to be operable within a few seconds to be successful.

Now that the submarine is no longer an effective weapon in naval operations, the days of camouflaging ships in wartime have passed. It is unfortunate that so little was accomplished during the period of hostilities because better results might have saved numerous ships and lives otherwise lost. In fact, camouflage made no more than a ten percent contribution to safety^[[53]](#footnote-53)^.

**Acknowledgement**

The writer is deeply indebted to Professor C. H. Peabody, of the Department of Naval Architecture and Marine Engineering at the Massachusetts Institute of Technology, for his kind suggestions and aid during the work and for making possible the use of the necessary equipment.

**Appendix A: Results Tables**

Tables A1-A12 provide the raw data for Designs 1-12, respectively. Values are in degrees. The error is the absolute difference between the physical direction of the model ship and the observer's setting of their perceived direction (Est) (two trials per condition). The column on the far right is the average error for each skyscape condition. These are the data plotted in the results figures. The second line from the bottom is the average of the entries above it. The bottom line is the average including assumed values of zero deg for nominal black and white backgrounds for which no data were reported. This is the dashed line in the results figures. The entries in bold denote calculation errors in the original report where > 1 deg.

| **Design 1** |  | | | | | | | | | |
| --- | --- | --- | --- | --- | --- | --- | --- | --- | --- | --- |
|  | **Obs 1** | | | **Obs 2** | | | **Obs 3-6** | | | **Average** |
|  | **Physical** | **Est.** | **Error** | **Physical** | **Est.** | **Error** | **Physical** | **Est.** | **Error** |  |
| **Clear** | 140 | 110 | 30 | 64 | 280 | 216 | 50 | 64 | 14 | **38.00** |
|  | 60 | 190 | 130 | 52 | 68 | 16 | 122 | 90 | 32 |  |
| **Hazy** | 135 | 122 | 13 | 54 | 44 | 10 | 52 | 120 | 68 | **53.50** |
|  | 58 | 110 | **42** | 128 | 56 | 72 | 130 | 240 | 110 |  |
| **Storm** | 112 | 62 | 50 | 128 | 72 | 56 | 135 | 42 | 93 | **73.00** |
|  | 75 | 60 | 15 | 58 | 72 | 14 | 52 | 50 | 2 |  |
| **Cliffs** | 112 | 66 | 46 | 46 | 110 | 64 | 51 | 52 | 1 | 38.00 |
|  | 112 | 66 | 46 | 126 | 66 | 60 | 126 | 136 | 10 |  |
|  | **Average angle of error:** | | | | | | | | | 50 |
|  | **Average angle of error assuming no error for white or black backgrounds:** | | | | | | | | | **36** |

Table A1. Results for three observer sets and their averages for Design 1.

| **Design 2** |  | | | | | | | | | |
| --- | --- | --- | --- | --- | --- | --- | --- | --- | --- | --- |
|  | **Obs 1** | | | **Obs 2** | | | **Obs 3-6** | | | **Average** |
|  | **Physical** | **Est.** | **Error** | **Physical** | **Est.** | **Error** | **Physical** | **Est.** | **Error** |  |
| **Clear** | 232 | 300 | 68 | 292 | 294 | 2 | 302 | 322 | 20 | **61** |
|  | 225 | 318 | 93 | **-** | **-** | **-** | 235 | 296 | 61 |  |
| **Hazy** | 300 | 244 | 56 | 296 | 230 | 66 | 300 | 130 | 170 | 73 |
|  | 238 | 246 | 8 | 230 | 290 | 60 | 230 | 310 | 80 |  |
| **Storm** | 314 | 298 | 16 | 234 | 292 | 58 | 302 | 286 | 16 | 53.6 |
|  | 250 | 290 | 40 | 312 | 300 | 12 | 242 | 62 | 180 |  |
| **Cliffs** | 292 | 300 | 8 | 242 | 280 | 38 | 240 | 124 | 116 | 34.5 |
|  | 242 | 258 | 16 | 308 | 286 | 22 | 303 | 310 | 7 |  |
|  | **Average angle of error:** | | | | | | | | | **54** |
|  | **Average angle of error assuming no error for white or black backgrounds:** | | | | | | | | | 37 |

Table A2. Results for three observer sets and their averages for Design 2.

| **Design 3** |  | | | | | | | | | |
| --- | --- | --- | --- | --- | --- | --- | --- | --- | --- | --- |
|  | **Obs 1** | | | **Obs 2** | | | **Obs 3-6** | | | **Average** |
|  | **Physical** | **Est.** | **Error** | **Physical** | **Est.** | **Error** | **Physical** | **Est.** | **Error** |  |
| **Clear** | 46 | 126 | 80 | 57 | 56 | 1 | 60 | 120 | 60 | **38.4** |
|  | 49 | 52 | 3 | 118 | 70 | 48 | 130 | 130 | 0 |  |
| **Hazy** | 112 | 118 | 6 | 112 | 90 | 22 | 120 | 135 | 15 | **29.6** |
|  | 58 | 30 | 28 | 60 | 60 | 0 | 50 | 126 | 76 |  |
| **Storm** | 120 | 70 | 50 | 60 | 58 | 2 | 128 | 54 | 74 | 32 |
|  | 140 | 150 | 10 | 176 | 54 | **52** | 64 | 58 | 6 |  |
| **Cliffs** | 110 | 60 | 50 | 120 | 76 | 44 | 56 | 150 | 94 | 45 |
|  | 120 | 60 | 60 | 52 | 60 | **12** | 130 | 120 | 10 |  |
|  | **Average angle of error:** | | | | | | | | | 36.2 |
|  | **Average angle of error assuming no error for white or black backgrounds:** | | | | | | | | | 24 |

Table A3. Results for three observer sets and their averages for Design 3.

| **Design 4** |  | | | | | | | | | |
| --- | --- | --- | --- | --- | --- | --- | --- | --- | --- | --- |
|  | **Obs 1** | | | **Obs 2** | | | **Obs 3-6** | | | **Average** |
|  | **Physical** | **Est.** | **Error** | **Physical** | **Est.** | **Error** | **Physical** | **Est.** | **Error** |  |
| **Clear** | 46 | 54 | 8 | 47 | 74 | 27 | 120 | 60 | 60 | 30 |
|  | 114 | 86 | 28 | 128 | 77 | 51 | 235 | 228 | 7 |  |
| **Hazy** | 54 | 118 | 64 | 126 | 62 | 64 | 50 | 64 | 14 | 40.6 |
|  | 116 | 124 | 8 | 128 | 112 | 16 | 128 | 50 | 78 |  |
| **Storm** | 120 | 110 | 10 | 44 | 60 | 16 | 118 | 60 | 58 | 21.6 |
|  | 42 | 45 | 3 | 117 | 80 | 37 | 54 | 48 | 6 |  |
| **Cliffs** | 120 | 62 | 58 | 46 | 56 | 10 | 128 | 54 | 74 | **29.6** |
|  | 78 | 76 | 2 | 122 | 80 | 42 | 35 | 148 | 113 |  |
|  | **Average angle of error:** | | | | | | | | | **35.5** |
|  | **Average angle of error assuming no error for white or black backgrounds:** | | | | | | | | | 20.3 |

Table A4. Results for three observer sets and their averages for Design 4.

| **Design 5** |  | | | | | | | | | |
| --- | --- | --- | --- | --- | --- | --- | --- | --- | --- | --- |
|  | **Obs 1** | | | **Obs 2** | | | **Obs 3-6** | | | **Average** |
|  | **Physical** | **Est.** | **Error** | **Physical** | **Est.** | **Error** | **Physical** | **Est.** | **Error** |  |
| **Clear** | 125 | 112 | 13 | 128 | 114 | 14 | 125 | 68 | 57 | 28 |
|  | 46 | 38 | 8 | 126 | 120 | 6 | 126 | 54 | 72 |  |
| **Hazy** | 130 | 146 | 16 | 80 | 100 | 20 | 120 | 280 | 160 | 52 |
|  | 50 | 64 | 14 | 142 | 102 | 40 | 160 | 98 | 62 |  |
| **Storm** | 48 | 72 | 24 | 126 | 78 | 48 | 60 | 86 | 26 | 22 |
|  | 122 | 110 | 12 | 52 | 68 | 16 | 48 | 50 | 2 |  |
| **Cliffs** | 122 | 70 | 52 | 49 | 62 | 13 | 128 | 114 | 14 | **62.5** |
|  | 60 | 68 | 8 | 120 | 116 | 4 | 136 | 116 | 20 |  |
|  | **Average angle of error:** | | | | | | | | | **41.10** |
|  | **Average angle of error assuming no error for white or black backgrounds:** | | | | | | | | | **28.00** |

Table A5. Results for three observer sets and their averages for Design 5.

| **Design 6** |  | | | | | | | | | |
| --- | --- | --- | --- | --- | --- | --- | --- | --- | --- | --- |
|  | **Obs 1** | | | **Obs 2** | | | **Obs 3-6** | | | **Average** |
|  | **Physical** | **Est.** | **Error** | **Physical** | **Est.** | **Error** | **Physical** | **Est.** | **Error** |  |
| **Clear** | 45 | 124 | 79 | 49 | 74 | 25 | 114 | 112 | 2 | **21** |
|  | 52 | 130 | **8** | 134 | 96 | 38 | 46 | 50 | 4 |  |
| **Hazy** | 138 | 42 | 96 | 64 | 100 | 36 | 122 | 128 | 6 | 47 |
|  | 130 | 70 | 60 | 136 | 130 | 6 | 42 | 120 | 78 |  |
| **Storm** | 122 | 66 | 56 | 38 | 72 | 34 | 131 | 60 | 71 | 35.3 |
|  | 46 | 34 | 12 | 32 | 60 | 28 | 309 | 300 | 9 |  |
| **Cliffs** | 60 | 42 | 18 | 41 | 66 | 25 | 123 | 92 | 31 | 33.3 |
|  | 42 | 120 | 78 | 134 | 106 | 28 | 72 | 52 | 20 |  |
|  | **Average angle of error:** | | | | | | | | | **38.00** |
|  | **Average angle of error assuming no error for white or black backgrounds:** | | | | | | | | | 22.70 |

Table A6. Results for three observer sets and their averages for Design 6.

| **Design 7** |  | | | | | | | | | |
| --- | --- | --- | --- | --- | --- | --- | --- | --- | --- | --- |
|  | **Obs 1** | | | **Obs 2** | | | **Obs 3-6** | | | **Average** |
|  | **Physical** | **Est.** | **Error** | **Physical** | **Est.** | **Error** | **Physical** | **Est.** | **Error** |  |
| **Clear** | 230 | 252 | 22 | 232 | 260 | **52** | 303 | 302 | 1 | **17.5** |
|  | 216 | 220 | 4 | 307 | 294 | 13 | 130 | 150 | 20 |  |
| **Hazy** | 312 | 240 | 72 | 308 | 314 | 6 | 232 | 240 | 8 | 25 |
|  | 238 | 240 | 2 | 232 | 290 | 58 | 306 | 308 | 2 |  |
| **Storm** | 242 | 60 | **178** | 300 | 260 | 40 | 305 | 350 | 45 | **62** |
|  | 314 | 314 | 0 | 306 | 270 | 36 | 232 | 220 | 12 |  |
| **Cliffs** | 232 | 304 | 72 | 302 | 250 | 52 | 303 | 302 | 2 | **63** |
|  | 230 | 270 | 40 | 232 | 260 | 28 | 224 | 40 | **174** |  |
|  | **Average angle of error:** | | | | | | | | | **39.20** |
|  | **Average angle of error assuming no error for white or black backgrounds:** | | | | | | | | | 28.00 |

Table A7. Results for three observer sets and their averages for Design 7.

| **Design 8** |  | | | | | | | | | |
| --- | --- | --- | --- | --- | --- | --- | --- | --- | --- | --- |
|  | **Obs 1** | | | **Obs 2** | | | **Obs 3-6** | | | **Average** |
|  | **Physical** | **Est.** | **Error** | **Physical** | **Est.** | **Error** | **Physical** | **Est.** | **Error** |  |
| **Clear** | 238 | 108 | 130 | 235 | 240 | **15** | 230 | 224 | 6 | 40.8 |
|  | 294 | 298 | 4 | 226 | 286 | 60 | 310 | 280 | 30 |  |
| **Hazy** | 230 | 248 | 18 | 242 | 282 | 40 | 310 | 300 | 10 | 29.6 |
|  | 230 | 312 | 82 | 224 | 242 | 18 | 238 | 242 | 4 |  |
| **Storm** | 230 | 310 | 80 | 301 | 292 | 9 | 305 | 250 | 55 | **56.4** |
|  | 130 | 130 | 0 | 232 | 250 | 18 | 128 | 238 | 110 |  |
| **Cliffs** | 226 | 338 | 112 | 240 | 264 | 24 | 313 | 310 | 3 | 34.5 |
|  | 280 | 250 | 30 | 298 | 280 | 18 | 233 | 252 | 19 |  |
|  | **Average angle of error:** | | | | | | | | | **36.8** |
|  | **Average angle of error assuming no error for white or black backgrounds:** | | | | | | | | | 27 |

Table A8. Results for three observer sets and their averages for Design 8.

| **Design 9** |  | | | | | | | | | |
| --- | --- | --- | --- | --- | --- | --- | --- | --- | --- | --- |
|  | **Obs 1** | | | **Obs 2** | | | **Obs 3-6** | | | **Average** |
|  | **Physical** | **Est.** | **Error** | **Physical** | **Est.** | **Error** | **Physical** | **Est.** | **Error** |  |
| **Clear** | 222 | 30 | **168** | 294 | 284 | 10 | 236 | 232 | 4 | 37 |
|  | 236 | 240 | 4 | 238 | 247 | 9 | 248 | 222 | 26 |  |
| **Hazy** | 220 | 62 | **168** | 226 | 310 | 84 | 240 | 280 | 40 | 56 |
|  | 308 | 294 | 14 | 304 | 290 | 14 | 310 | 294 | 16 |  |
| **Storm** | 306 | 298 | 8 | 240 | 260 | 20 | 246 | 214 | **132** | 39 |
|  | 230 | 288 | 58 | 300 | 288 | 12 | 306 | 300 | 6 |  |
| **Cliffs** | 288 | 296 | 8 | 243 | 274 | 31 | 237 | 294 | 57 | 30 |
|  | 230 | 240 | 10 | 324 | 280 | 44 | 310 | 240 | **30** |  |
|  | **Average angle of error:** | | | | | | | | | **58.90** |
|  | **Average angle of error assuming no error for white or black backgrounds:** | | | | | | | | | 27.00 |

Table A9. Results for three observer sets and their averages for Design 9.

| **Design 10** |  | | | | | | | | | |
| --- | --- | --- | --- | --- | --- | --- | --- | --- | --- | --- |
|  | **Obs 1** | | | **Obs 2** | | | **Obs 3-6** | | | **Average** |
|  | **Physical** | **Est.** | **Error** | **Physical** | **Est.** | **Error** | **Physical** | **Est.** | **Error** |  |
| **Clear** | 229 | 240 | 11 | 311 | 296 | 15 | 310 | 230 | 80 | 23 |
|  | 310 | 308 | 2 | 234 | 312 | **22** | 56 | 46 | 10 |  |
| **Hazy** | 220 | 220 | 0 | 308 | 260 | 48 | 300 | 302 | 2 | 32 |
|  | 324 | 224 | 100 | 218 | 240 | 22 | 223 | 242 | 19 |  |
| **Storm** | 304 | 312 | 8 | 308 | 300 | **2** | 306 | 90 | **16** | 30 |
|  | 226 | 226 | 0 | 230 | 294 | 64 | 141 | 50 | 91 |  |
| **Cliffs** | 228 | 308 | 80 | 312 | 297 | 15 | 250 | 110 | 140 | **37** |
|  | 306 | 294 | 12 | 231 | 254 | **77** | 320 | 320 | 0 |  |
|  | **Average angle of error:** | | | | | | | | | **38.90** |
|  | **Average angle of error assuming no error for white or black backgrounds:** | | | | | | | | | 20.00 |

Table A10. Results for three observer sets and their averages for Design 10.

| **Design 11** |  | | | | | | | | | |
| --- | --- | --- | --- | --- | --- | --- | --- | --- | --- | --- |
|  | **Obs 1** | | | **Obs 2** | | | **Obs 3-6** | | | **Average** |
|  | **Physical** | **Est.** | **Error** | **Physical** | **Est.** | **Error** | **Physical** | **Est.** | **Error** |  |
| **Clear** | 123 | 62 | **59** | 130 | 96 | 34 | 128 | 52 | 76 | 41 |
|  | 115 | 58 | 57 | 52 | 56 | 4 | 312 | 296 | 16 |  |
| **Hazy** | 130 | 128 | 2 | 130 | 110 | 20 | 122 | 70 | 52 | **29.6** |
|  | 128 | 48 | 80 | 35 | 45 | 10 | 56 | 60 | 4 |  |
| **Storm** | 122 | 80 | 42 | 56 | 76 | 20 | 114 | 62 | 52 | **44.6** |
|  | 46 | 62 | 16 | 122 | 96 | 26 | 52 | 220 | **178** |  |
| **Cliffs** | 122 | 104 | 18 | 55 | 72 | **19** | 40 | 72 | 32 | **32** |
|  | 68 | 100 | 32 | 123 | 96 | 27 | 56 | 110 | 54 |  |
|  | **Average angle of error:** | | | | | | | | | **39.20** |
|  | **Average angle of error assuming no error for white or black backgrounds:** | | | | | | | | | **22.00** |

Table A11. Results for three observer sets and their averages for Design 11.

| **Design 12** |  | | | | | | | | | |
| --- | --- | --- | --- | --- | --- | --- | --- | --- | --- | --- |
|  | **Obs 1** | | | **Obs 2** | | | **Obs 3-6** | | | **Average** |
|  | **Physical** | **Est.** | **Error** | **Physical** | **Est.** | **Error** | **Physical** | **Est.** | **Error** |  |
| **Clear** | 119 | 58 | 61 | 130 | 96 | 34 | 45 | 135 | 90 | **22.5** |
|  | 120 | 60 | 60 | 58 | 70 | 12 | 310 | 230 | 80 |  |
| **Hazy** | 130 | 58 | 72 | 120 | 62 | **68** | 48 | 54 | 6 | 29.6 |
|  | 60 | 50 | 10 | 68 | 90 | 22 | 310 | 310 | 0 |  |
| **Storm** | 38 | 52 | 14 | 54 | 66 | 12 | 50 | 310 | 100 | 44.6 |
|  | 98 | 128 | 30 | 122 | 94 | 28 | 50 | 134 | 84 |  |
| **Cliffs** | 38 | 50 | 12 | 47 | 44 | 3 | 121 | 80 | 41 | 32 |
|  | 122 | 110 | 12 | 126 | 102 | 24 | 42 | 142 | 100 |  |
|  | **Average angle of error:** | | | | | | | | | **40.50** |
|  | **Average angle of error assuming no error for white or black backgrounds:** | | | | | | | | | 22.00 |

Table A12. Results for three observer sets and their averages for Design 12.

1. This is not made explicit but is implied from what is described about the methods with respect to the first of six observers. [↑](#footnote-ref-1)
2. This figure was derived from an independent calculation of the averages of the average observer values reported by Blodgett for each of 12 ship designs and 4 skyscapes (i.e. the average of 48 reported values). [↑](#footnote-ref-2)
3. Blodgett was not explicit about where the following list of findings/claims come from, but given their position in the thesis, presumably they derive from the exploits of the research team under discussion. [↑](#footnote-ref-3)
4. It is unclear what Blodgett meant by 'superimposed'. [↑](#footnote-ref-4)
5. Both this quote and the previous one come from Bates (1918) and have been slightly revised to match the original text. [↑](#footnote-ref-5)
6. It is unclear where this figure of 15 deg comes from, nor the subsequent figures regarding speed and distance, not least since distance is not needed in calculating the firing solution. An estimate of distance is helpful in estimating speed (as Blodgett reports) and identifying class, but is of no concern independent of these factors, contrary to what Blodgett appeared to imply. [↑](#footnote-ref-6)
7. It is unclear where these figures come from. [↑](#footnote-ref-7)
8. It is unclear what Blodgett meant by a 'definite scheme' (even in the context of footnote 10), but possibly it relates to ideas around forced perspective. [↑](#footnote-ref-8)
9. Blodgett made no attempt to do this. [↑](#footnote-ref-9)
10. This word, though typically corrected or deleted (as superfluous) from modern undergraduate dissertations, was not uncommon in official documents of the period (e.g., Van Buskirk, 1919a). It seems the term referred to specific decisions, observations or principles (etc.), sometimes in the context of officialdom. [↑](#footnote-ref-10)
11. This section was extracted from the original foreword. [↑](#footnote-ref-11)
12. The original term is 'screens' rather than filters. It seems that colour filters were used as a countermeasure to the use of colour in camouflage. It is unclear what is meant precisely by 'not affected'. [↑](#footnote-ref-12)
13. But there is no sign of this in the thesis; perhaps 'mathematical' is not the word Blodgett was after. [↑](#footnote-ref-13)
14. It is unclear what Blodgett was driving at here. He used the words: 'seem possible', which has been edited to 'reasonably possible'. [↑](#footnote-ref-14)
15. It is unclear what Blodgett was driving at here. This is a best attempt at conveying intended meaning. [↑](#footnote-ref-15)
16. It is unclear where this figure comes from. The predicate of this statement is also mysterious. [↑](#footnote-ref-16)
17. Presumably, this is what Blodgett meant here. [↑](#footnote-ref-17)
18. The following sentence was deleted from the following text, because its meaning was unclear, and it seemed misplaced: "This method really carries out any hull design and more or less accentuates contrasts." [↑](#footnote-ref-18)
19. This reconsideration does not take place explicitly but might refer to the "reverse perspective" of Design 6. [↑](#footnote-ref-19)
20. This is a best guess at a sentence that was difficult to follow. [↑](#footnote-ref-20)
21. Blodgett did not define what he meant by 'best'. [↑](#footnote-ref-21)
22. These colour swatches are from an electronic copy (pdf) of the original materials. It is possible that these colours do not convey the original colours of the paints owing to degradation over time. [↑](#footnote-ref-22)
23. This meaning of this sentence is unclear. [↑](#footnote-ref-23)
24. Blodgett does not report whether the same design was applied to each side of the vessel but presumably this was the case since only one side is shown for each design (Figures 3 & 5). [↑](#footnote-ref-24)
25. The atribution to BCD is the best that can be discerned from the original document but uncertainty remains over where the experiments were conducted. [↑](#footnote-ref-25)
26. From Blodgett's writing it is unclear whether several of the design issues were solved himself, or by the designers of the equipment. The latter seems more likely. [↑](#footnote-ref-26)
27. It is unclear whether the sea was put in motion during the observations but the text here implies that it might have been. [↑](#footnote-ref-27)
28. This section was unclear in the original; this is a best guess of the meaning intended. [↑](#footnote-ref-28)
29. This section was unclear in the original; this is a best guess of the meaning intended. The reference to the horizontal light carriers remains mysterious. [↑](#footnote-ref-29)
30. Blodgett did not say, but presumably these observations were intended only for the experimenter, not the participants. [↑](#footnote-ref-30)
31. Presumably, this is what Blodgett meant here. [↑](#footnote-ref-31)
32. Blodgett did not report which observers took part in each of the two simulated distances. [↑](#footnote-ref-32)
33. Alternatively, it might be that Blodgett intended to say: '...a study of the methods of precision measurements...' [↑](#footnote-ref-33)
34. Blodgett does not make clear what he means by Obs 3 in his original tables. Presumably, he refers to the average of observers 3-6 in the participants section. [↑](#footnote-ref-34)
35. Blodgett does not report these results, nor the observer or observers who took part, nor the number of observations. [↑](#footnote-ref-35)
36. The implication is that no data were gathered for this condition. [↑](#footnote-ref-36)
37. Blodgett does not calculate these 'areas'. [↑](#footnote-ref-37)
38. Blodgett does not report how many observers were involved in this part of the experiment nor who they were. His writing neither ruled out nor implied in, the six observers mentioned in the Participants section. [↑](#footnote-ref-38)
39. This is the average across the six entries along the abscissa, including the white and black conditions. The relevant term should probably be 'background conditions' rather than 'conditions of weather'. [↑](#footnote-ref-39)
40. It was difficult to decipher precise meaning here, but the gist is probably correct. [↑](#footnote-ref-40)
41. It is not clear whether Blodgett means that he found these to be the most effective colours, or whether he is using the word 'most' in a most old-fashioned English sense to mean 'rather', or 'very'. [↑](#footnote-ref-41)
42. It is unclear what this means. As earlier, the word 'screens' has been replaced by 'filters' in the edit (also in the next sentence). But the point is unclear, nonetheless. Perhaps the aim is to diminish an effect from a height inferred but not illustrated. [↑](#footnote-ref-42)
43. This is unclear, but presumably refers to a countermeasure strategy for overcoming the colours in dazzle camouflage by using colour filters. [↑](#footnote-ref-43)
44. It is unclear what this is referring to; possibly a limit in the experiment on trial length. [↑](#footnote-ref-44)
45. The design in Figure 3, top-left (Design 1) shows two masts, yet the photograph (insert of Figure 4, top-left) seems to show only one, as implied by Blodgett's text. [↑](#footnote-ref-45)
46. It is unclear where the basis for this statement about colour switching comes from. [↑](#footnote-ref-46)
47. It is difficult to see where the basis for this statement comes from. [↑](#footnote-ref-47)
48. This is puzzling because the simulated distances are reportedly 1,100 yards and 2,200 yards. [↑](#footnote-ref-48)
49. There was no mention of this for the earlier designs, so the word 'again' is mysterious, but it might relate to Design 5. [↑](#footnote-ref-49)
50. Blodgett's photographs of the three passenger ships (Designs 7-9, insets in Figure 6) appear to be for a different ship shape from those in the camouflage design drawings (Designs 7-9, Figures 5), perhaps more like a tramp with superstructure amidships (Designs 4-6, Figures 3 and 4). [↑](#footnote-ref-50)
51. Presumably, this was Blodgett's point. [↑](#footnote-ref-51)
52. It is unclear where this colour switching comes from. [↑](#footnote-ref-52)
53. It is unclear where this figure and assessment come from. [↑](#footnote-ref-53)
