# Supplementary material for: Blodgett's (1919) “Ship camouflage” 105 years on: A misperception of dazzle perception revealed and redressed
Source: Iperception. 2025 Mar 14;16(2):20416695241312316. doi: 10.1177/20416695241312316 (PMC11909666; doi:10.1177/20416695241312316)
Supplement: sj-docx-3-ipe-10.1177_20416695241312316 - Supplemental material for Blodgett's (1919) “Ship camouflage” 105 years on: A misperception of dazzle perception revealed and redressed [file sj-docx-3-ipe-10.1177_20416695241312316.docx]

**Blodgett's (1919) "Ship Camouflage" 105 years on: A dazzling misperception of dazzle perception revealed and redressed**

Meese, T. S. & Strong, S. L. (2025), *i-Perception.*

**Supplementary Material 3: Results per ship design and notes on sign preservation in averaging**


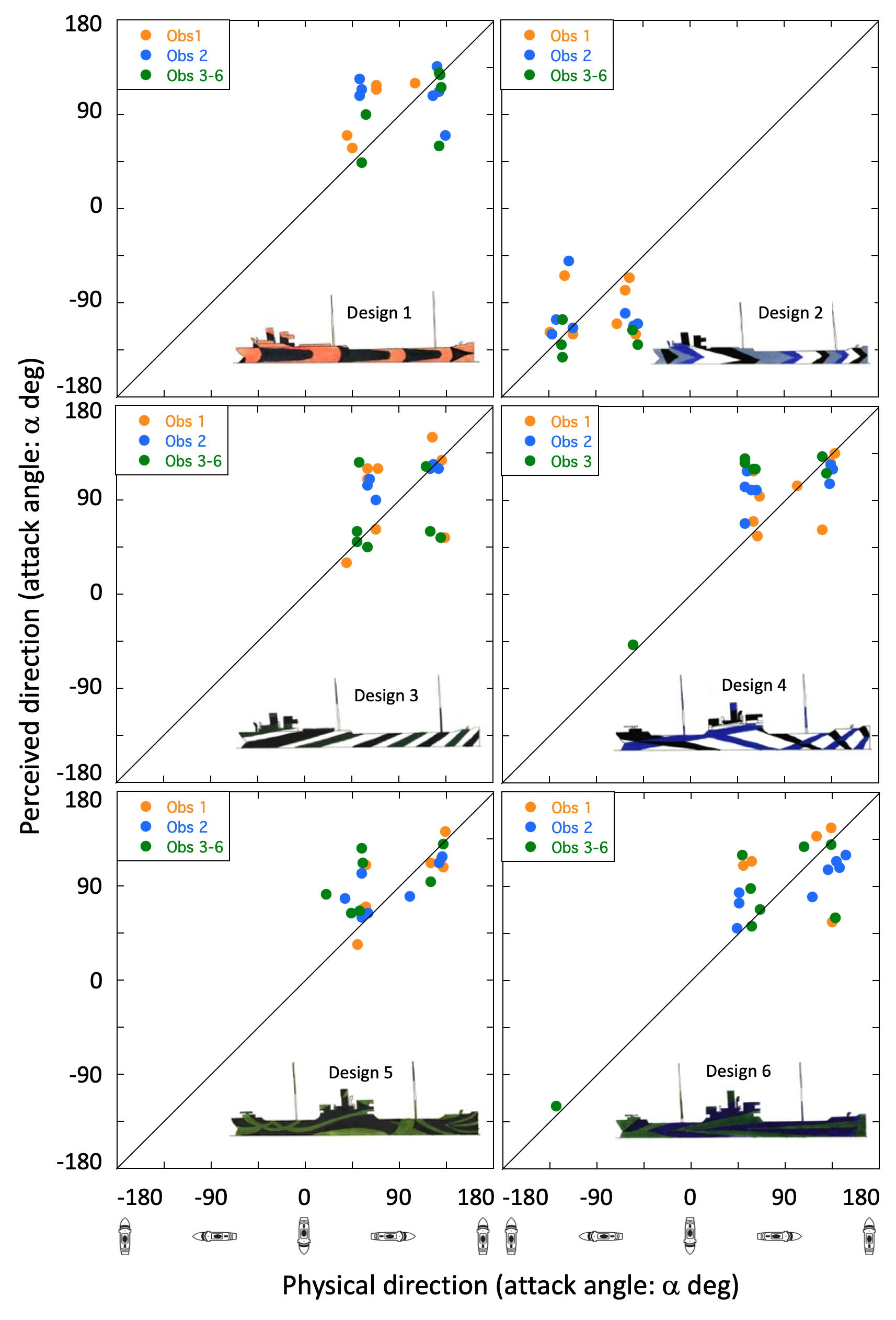


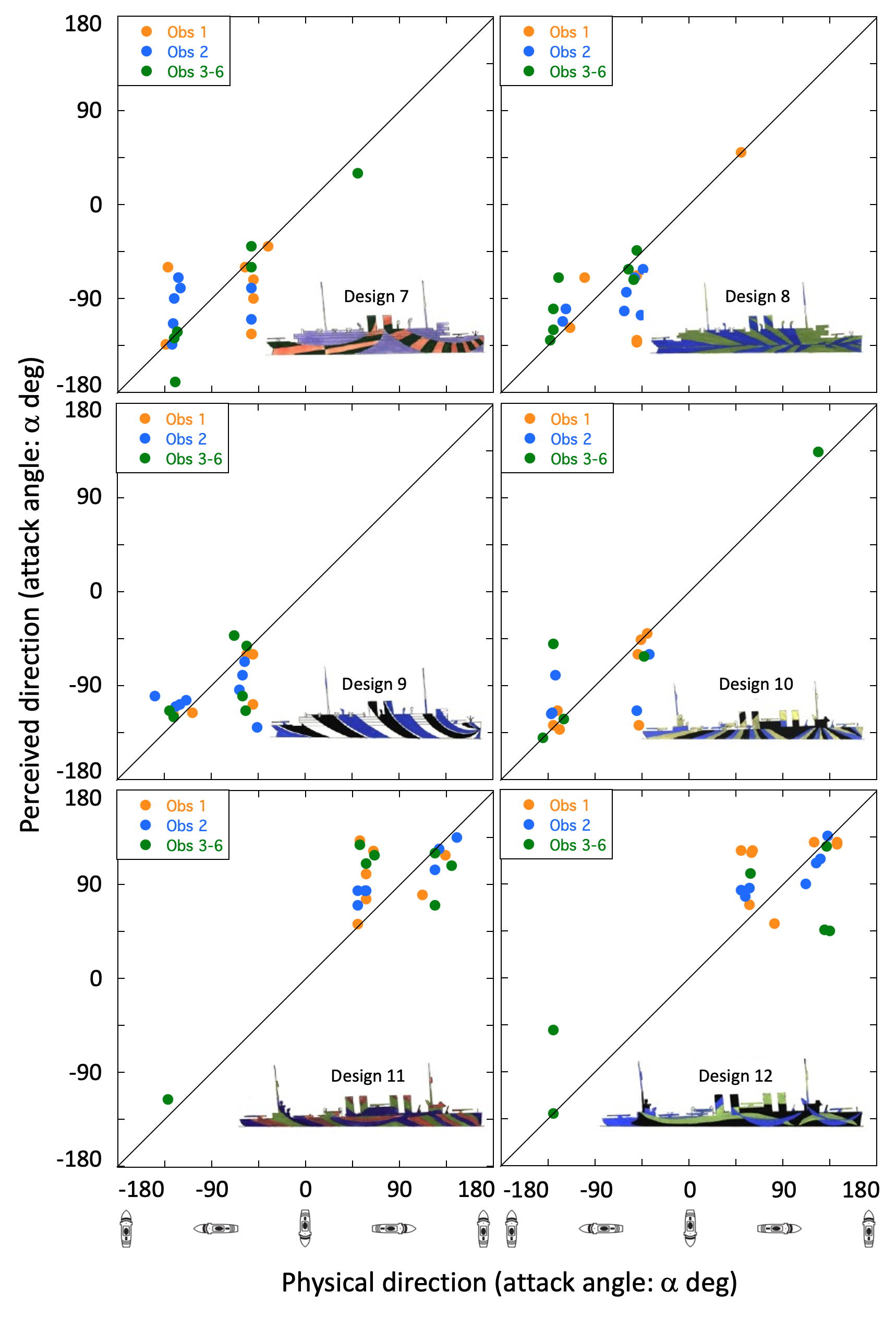


Figure S3.1. For completeness we show scatter plots here for each of the twelve camouflage designs. Different colour symbols are for different observers collapsed across skyscape. The direction axes are for target angle, α. This involves a transform from the compass angle convention thought to be used by Blodgett (1919) but is more intuitive (see Figure 4 in main report for details). The oblique lines in the figures are contours of veridicality and the icons depict ships travelling in a direction (α) of 90 deg. Note that in most cases there are two clusters of data (determined by the experimenter's choice of physical (actual) direction) with occasional outliers. These outliers are from mirror reversed physical ship directions and were systematically pooled with the main clusters as described in the main body of the report. Note also that within most clusters, there are perceived direction errors (deviations from veridicality) that span both sides of the contour of verdicality which means the sign of perceptual errors should be preserved in the data analysis. Note further that within each panel, one of the main clusters is dominated by positive errors (the perceived attack angles (data points) lie above the contour of veridicality) while the other one is dominated by negative errors (the perceived attack angles (data points) lie below the contour of veridicality). This difference across clusters is due to the hysteresis effect (*h*). It means that when combining the two clusters to calculate an overall average error (e.g., Figure 6c in the main report), the error signs in one of the clusters must first be flipped (otherwise the perceptual errors for a pure hysteresis effect would average to zero). The average standard deviation of responses within the 24 clusters above (including mirror reversals) was 26.9 deg.
