# Supplementary material for: Blodgett's (1919) “Ship camouflage” 105 years on: A misperception of dazzle perception revealed and redressed
Source: Iperception. 2025 Mar 14;16(2):20416695241312316. doi: 10.1177/20416695241312316 (PMC11909666; doi:10.1177/20416695241312316)
Supplement: sj-docx-4-ipe-10.1177_20416695241312316 - Supplemental material for Blodgett's (1919) “Ship camouflage” 105 years on: A misperception of dazzle perception revealed and redressed [file sj-docx-4-ipe-10.1177_20416695241312316.docx]

**Blodgett's (1919) "Ship Camouflage" 105 years on: A dazzling misperception of dazzle perception revealed and redressed**

Meese, T. S. & Strong, S. L. (2025), *i-Perception.*

**Supplementary Material 4: Reinstation of results from Blodgett**

Table S4.1 lists the 9 responses (perceived directions) and their corresponding conditions where the absolute perceptual error was greater than 90 deg but the confusion was not one of bow and stern. All of these results were for either Observer 1 or Observers 3-6. None were for Observer 2.

| Actual direction (deg) | Perceived direction (deg) | Signed error  (deg) (perceived-actual) | Observer | Ship design  (Figure 3) | Skyscape |
| --- | --- | --- | --- | --- | --- |
| 225 | 318 | 93 | 1 | 2 | Clear |
| 226 | 338 | 112 | 1 | 8 | Cliffs |
| 324 | 224 | -100 | 1 | 10 | Hazy |
| 138 | 42 | -96 | 1 | 6 | Hazy |
| 135 | 42 | -93 | 3-6 | 1 | Storm |
| 56 | 150 | 94 | 3-6 | 3 | Cliffs |
| 35 | 148 | 113 | 3-6 | 4 | Cliffs |
| 141 | 50 | -91 | 3-6 | 10 | Storm |
| 42 | 142 | 100 | 3-6 | 12 | Cliffs |

Table S4.1 Reinstated results for the analysis in this supplementary material. The directions/angles use the same convention as Blodgett (1919) which was assumed to be the compass convention (Figure 4a in main report).

Figure S4.1. Perceived direction as a function of physical direction (*α*, including mirror reversals—i.e., angle on the bow) for different observers. The nine open symbols are the reinstated results for Observer 1 and Observers 3-6. The black and red diamonds show the means before and after data reinstation, respectively.

Figure S4.1 is adapted from Figure 7a in the main report with the reinstated results (Table S4.1) included (open symbols). This increases the average errors slightly (compare red and black diamonds), pulling them towards the horizon (target angle = 90 deg) but does not change the overall picture. Nonetheless, we performed the statistical analysis again for these two observers. Table S4.2, shows that reinstating these data did not change whether an individual result reached significance (compare with Table 3 in the main report). Our conclusions based around Table 3 are unchanged by the analysis here.

| **Row No.** | **Comparison** | **Observer** | **Lefthand cluster**  **(Figure 7a)** | | **Righthand cluster (Figure 7a)** | |
| --- | --- | --- | --- | --- | --- | --- |
|  |  |  | ***t*(DF)** | ***p*** | ***t*(DF)** | ***p*** |
| 1 | PE *vs* 0 deg | 1 | 19.7(52) | <0.001 ** | -13.1(34) | <0.001 ** |
| 2 | " | 3-6 | 16.4(38) | <0.001 ** | -13.3(43) | <0.001 ** |
| 3 | PTA *vs* 90 deg | 1 | 0.14(52) | 0.888 | 3.35(34) | <0.001 ** |
| 4 | " | 3-6 | -0.56(38) | 0.576 | 2.15(43) | 0.037 * |
| 5 | MPE: LHC *vs* RHC | 1 | 2.48(86) | 0.008 ** | - | - |
| 6 | " | 3-6 | 0.96(81) | 0.169 | - | - |

Table S4.2. Statistical analysis (t-tests) for the clusters of data in Figure S4.1. The first four rows are for two-tailed single sample t-tests. The bottom two rows are for one-tailed unmatched two-sample t-tests. The statistical significance or otherwise for the entries here are the same as the corresponding entries in Table 3 of the main report. PE = perceptual error; PTA = perceived target angle. MPE = magnitude of perceptual error. LHC, RHC = lefthand and righthand clusters. * = statistically significant. ** = statistically highly significant.

Finally, we re-considered our overall estimates of hysteresis (*h*) and twist (*w*) based on the full set of data (i.e., all 12 designs). In general, here and in the main report, these were calculated by first deriving *h* and *w* for each side of the mirror reversal (i.e., for physical directions of 0-180 deg, and 181 to 360 deg; see Figure 10 in the main report) and then averaging the two estimates of each parameter. This gives slightly different values from deriving these parameters directly from the complete mirror reversed data set because of Blodgett's (1919) uneven distribution of experimental trials across physical directions, but the differences are slight. For the full set of 12 designs, we found *h* = 24.7 deg, and *w* = 6.7 deg, before reinstating the results in Table S4.1, and *h* = 27.4 deg, and *w* = 6.8 deg, with those results reinstated. (Note that the reports of *w* and *h* in the main report exclude design 6.) The influence of reinstating the results in Table S4.1 on estimates of *h* and *w* is small and unimportant.
