# Supplementary material for: Blodgett's (1919) “Ship camouflage” 105 years on: A misperception of dazzle perception revealed and redressed
Source: Iperception. 2025 Mar 14;16(2):20416695241312316. doi: 10.1177/20416695241312316 (PMC11909666; doi:10.1177/20416695241312316)

**Blodgett's (1919) "Ship Camouflage" 105 years on: A dazzling misperception of dazzle perception revealed and redressed**

Meese, T. S. & Strong, S. L. (2025), *i-Perception.*

**Supplementary Material 5: The two instruction slides used in the new experiment**

------------------------------------------------------------------


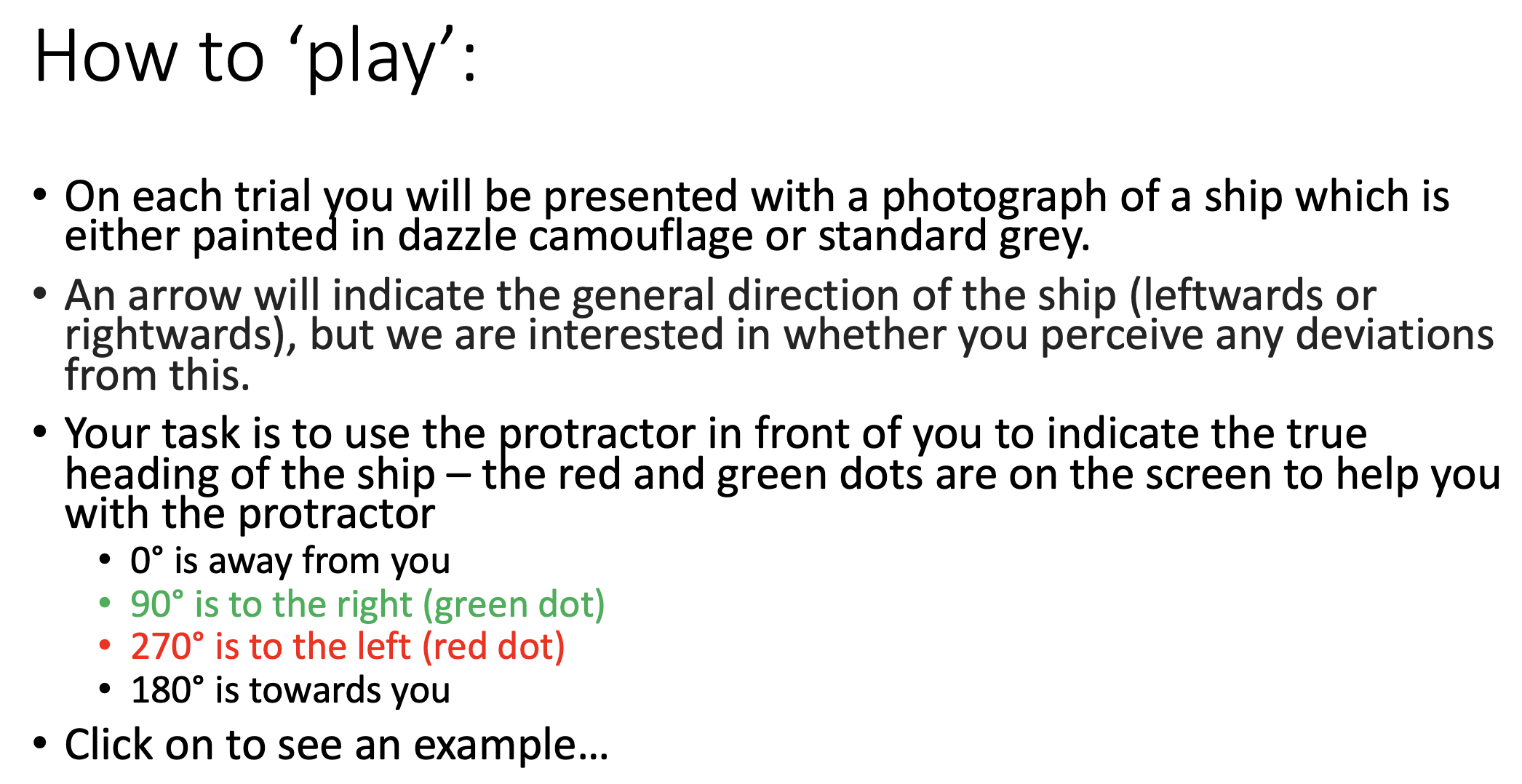


------------------------------------------------------------------


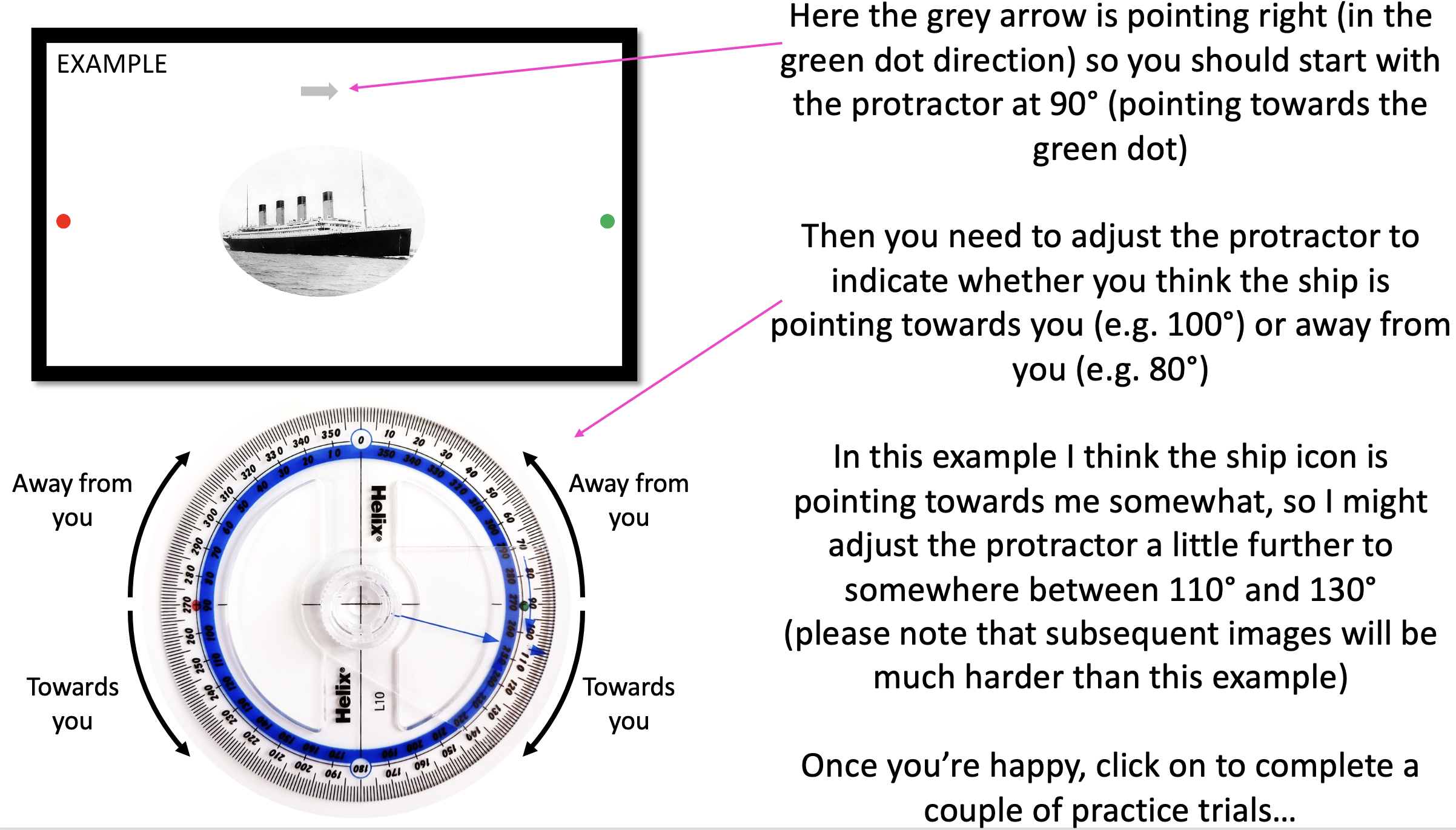

Supplement: sj-docx-5-ipe-10.1177_20416695241312316 - Supplemental material for Blodgett's (1919) “Ship camouflage” 105 years on: A misperception of dazzle perception revealed and redressed [file sj-docx-5-ipe-10.1177_20416695241312316.docx]
