# Supplementary material for: Blodgett's (1919) “Ship camouflage” 105 years on: A misperception of dazzle perception revealed and redressed
Source: Iperception. 2025 Mar 14;16(2):20416695241312316. doi: 10.1177/20416695241312316 (PMC11909666; doi:10.1177/20416695241312316)
Supplement: sj-docx-6-ipe-10.1177_20416695241312316 - Supplemental material for Blodgett's (1919) “Ship camouflage” 105 years on: A misperception of dazzle perception revealed and redressed [file sj-docx-6-ipe-10.1177_20416695241312316.docx]

**Blodgett's (1919) "Ship Camouflage" 105 years on: A dazzling misperception of dazzle perception revealed and redressed**

Meese, T. S. & Strong, S. L. (2025), *i-Perception.*

**Supplementary Material 6: Results of the colour grouping experiment**

Table S6.1 shows the groupings of Blodgett's twelve ships (see Figure 3 in the main report) by each of our sixteen naive participants who also conducted the new control experiment. The red asterisks show the groupings used in Figure 6d. The blue asterisk is for the design (D11) that was excluded from the analysis owing to the inconsistency between observers over its grouping.

Table S6.1 Results from the colour grouping experiment.
