# Supplementary material for: Blodgett's (1919) “Ship camouflage” 105 years on: A misperception of dazzle perception revealed and redressed
Source: Iperception. 2025 Mar 14;16(2):20416695241312316. doi: 10.1177/20416695241312316 (PMC11909666; doi:10.1177/20416695241312316)
Supplement: sj-docx-12-ipe-10.1177_20416695241312316 - Supplemental material for Blodgett's (1919) “Ship camouflage” 105 years on: A misperception of dazzle perception revealed and redressed [file sj-docx-12-ipe-10.1177_20416695241312316.docx]

**Blodgett's (1919) "Ship Camouflage" 105 years on: A dazzling misperception of dazzle perception revealed and redressed**

Meese, T. S. & Strong, S. L. (2025), *i-Perception.*

This Supplementary Material is a slightly more detailed version of the Results and Discussion from the paper above.

**3.3 Results and Discussion**

In Blodgett (1919), results were shown as absolute perceptual errors averaged across observers (with observers 3-6 seemingly treated as a single item in the average) for each skyscape. This was plotted separately for each dazzle design, making comparisons difficult. Here, we replot these results for each design in Figure 6a. The ordinate is the absolute (i.e., unsigned) difference between actual direction and perceived direction. The figure shows substantial variation of perceptual errors across conditions, though no obvious systematic effects for dazzle design or skyscape. Each average in Figure 4a was derived from no more than twelve measures: two observations for each of six observers but weighted as if only a total of six observations across three observers. These were spread rather haphazardly across physical directions within the constraints described above (see S3 for details).


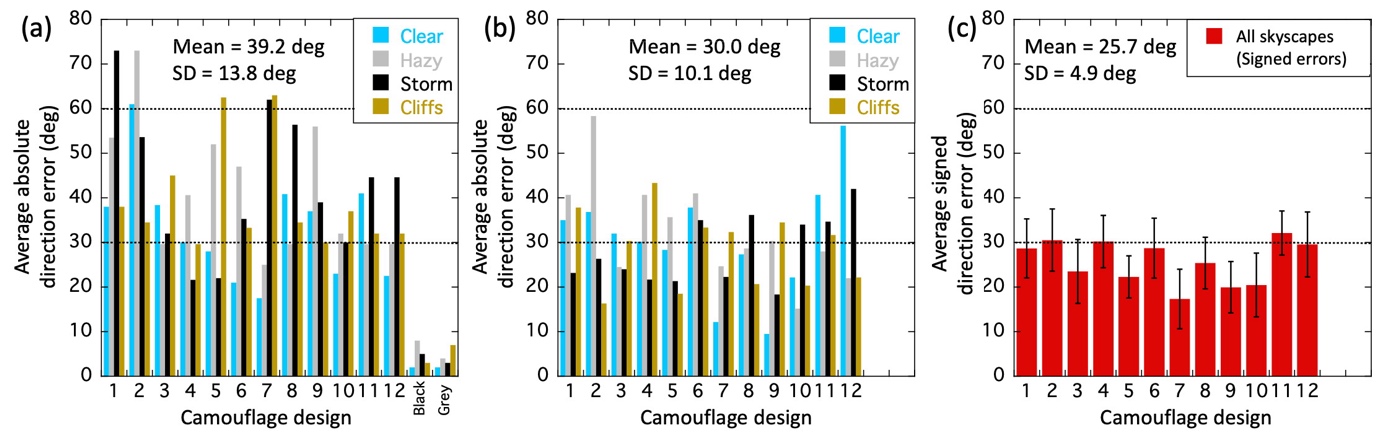


Figure 6. Experimental results (perceived direction errors) averaged across observers. (a) Blodgett's (1919) results as he reported them (averages of absolute direction errors) for each of the twelve camouflage designs (Figure 3) and the uniform black and grey control conditions for each of the four skyscapes (see legend). (b) The same as (a) after data cleaning but retaining absolute errors in the averaging (see text for details) and with the unsafe control results removed. (c) The same as (b) but using signed errors in the averaging and with results collapsed across the four skyscapes. Error bars are ±1SE of the signed perceptual error distributions in the pool. The means and SDs reported in each panel are calculated across the full set of bars excluding the black and grey control conditions. The horizontal dashed lines are arbitrary comparison lines at 30 and 60 deg.

Most notable in Figure 6a is (i) how large some of the absolute direction errors are (>60 deg) and (ii) how much greater they are for each of the twelve dazzle designs compared to the two neutral conditions (black and grey at the far right of the plot). However, as highlighted above, the validity of these control results is questionable, and we consider them unsafe. We return to these data in our overall conclusion after running our own control experiment designed to compare dazzle designs with neutral (grey) versions (c.f. Lovell et al, 2024).

**3.4 A revaluation of Blodgett's results**

It is fortunate that Blodgett (1919) tabulated his results (see appendices in either S1 or S2) because this permitted close examination and more detailed plotting and evaluation of what he measured. We identified several sources of error in Blodgett's presentation and worked through correcting each source in turn before inspecting the cleaned results. This procedure was put in place to protect against p-hacking and expectation bias by the current authors through the influence of cleaning decisions.

**3.4.1 Data cleaning**

We identified five different problems with Blodgett's data and his analysis, four of which are listed in Table 2. First, we found instances where the reported error was inconsistent with the difference between the actual and perceived directions (data column 1, Table 2). Since we cannot know whether this was a calculation error or a transcription error (which could relate to any of the three values involved), we removed these data from further analysis (these data are identified in the appendices of S1 and of S2). We considered this safe, since there was no reason to suppose these errors were specific to a particular feature in the results (e.g., large effect sizes).

Second, we found several cases where Blodgett's averaging across observer was wrong (data column 4, Table 2), but this held no obvious relation to the first problem. Therefore, we disregarded Blodgett's averages and performed these ourselves (we make a further point about averaging below).

Third, we found some cases where the difference between actual and perceived directions were greater than 90 deg (data column 2, Table 2; see also our archived data files referenced in the end matter). This can happen if the observer misidentifies the stern as the bow. While not without interest, we considered this to be a different class of error from our primary interest, and while it might occur at sea when single short observations are made, submariners typically tracked their potentially zig-zagging quarry (e.g., ONI, 1918a; Paterson, 2018; Florek, 2017a, b), making the persistence of this error unlikely, not least because of the clear giveaway from a steamship's smoke. Wilkinson, in Van Buskirk (1919a, p125), was also dismissive of this type of perceptual error. A second way in which perceptual errors can be greater than 90 deg is when a gross perceptual switch from veridicality occurs for whether the bow or stern is closer to the observer (e.g., a target angle of 90+50 deg is misperceived as an angle of 90-50 deg). Again, this struck us as a different class of perceptual error, perhaps more akin to the perceptual uncertainty/confusion that we discuss in Section 6.3. We also note that the simulated fog used by Blodgett might have contributed to both of these situations in an uncontrolled way (by obscuring small but tell tail forward and rear facing features of the superstructure, for example). Therefore, we followed Lovell et al (2024) and removed all results where perceptual errors were greater than 90 deg from further analysis. Of the 20 out of 288 trials where we did this (Table 2), 11 were for bow/stern confusions and all but one were for either Observer 1 (*n* = 6) or Observer 3-6 (*n* = 13). For completeness, in Supplementary Material 4 (S4), we reintroduce the 9 trials that were not bow/stern confusions and show that their exclusion was not critical for our main observer-based conclusions. And like Lovell et al (2024), neither did we notice anything of interest about the conditions that resulted in data removal.

| **Design** | **Differencing/24** | **Direction/24** | **Missing/24** | **Averaging/4** | **Removals/24** |
| --- | --- | --- | --- | --- | --- |
| 1 | 1 | 4 | 0 | 3 | 5 |
| 2 | 0 | 4 | 1 | 1 | 5 |
| 3 | 2 | 1 | 0 | 2 | 3 |
| 4 | 0 | 1 | 0 | 1 | 1 |
| 5 | 0 | 1 | 0 | 1 | 1 |
| 6 | 1 | 1 | 0 | 1 | 2 |
| 7 | 3 | 0 | 0 | 3 | 3 |
| 8 | 0 | 3 | 0 | 1 | 3 |
| 9 | 4 | 0 | 0 | 0 | 4 |
| 10 | 4 | 3 | 0 | 1 | 7 |
| 11 | 3 | 0 | 0 | 3 | 3 |
| 12 | 1 | 2 | 0 | 1 | 3 |
| **Total** | 19/288 | 20/288 | 1/288 | 18/48 | 40/288 |

Table 2. Anomalies in Blodgett's results tables (entries are number of trials). The 'differencing' column is for when the reported error was inconsistent with the difference between actual and perceived directions. The 'direction' error column is for when the error was greater than 90 deg (see text for details). The 'missing' column refers to missing data for trials. The 'averaging' column is for when Blodgett's average over the four skyscapes (the results he plotted) was wrong. For differencing and averaging, we worked with a tolerance of 1 deg. The final column lists the number of trials out of 24 (3 observers x 4 skyscapes x 2 trials) that were missing or removed (owing to differencing or direction errors) for each camouflage design (i.e., the sum of the first three columns). This totalled 40 over the entire experiment, leaving 248 of 288 trials intact for our analyses. In our own plots (i.e., all replots of Blodgett's data except Figure 6a), we discarded Blodgett's averages and recalculated these ourselves.

Fourth, there was one trial in Blodgett's results tables where no data were recorded (Design 2, Observer 2, clear skyscape; data column 3 in Table 2).

The fifth problem was with Blodgett's approach to averaging; he reported perceptual errors as the absolute (unsigned) differences between actual and perceived directions and averaged those, but this runs the risk of amplifying effect size. Consider a random variable with a mean of zero (e.g., a ship whose direction is seen veridically on average but is subject to random misperceptions from trial-to-trial). A signed average of sufficient samples of this variable will deliver a value of zero, but an average of absolute (unsigned) values will always be positive, its magnitude determined by the variance of the random variable. This is a problem for Blodgett's presentation if the data being pooled include perceived directions each side of veridicality. Observation of his data show this to be commonplace (e.g., see S3) highlighting a need for care with the perceptual error sign in our reanalysis.

Finally, we note that the individual data for each of Observers 3 to 6 might have been subject to any or all the problems above, but because only their (presumed) average was presented by Blodgett (1919), we could apply corrections only to the combined results. Nonetheless, and notwithstanding this obfuscation (imposing potential non-invertible nonlinearities), we found these cleaned results to be remarkably similar to those from each of the two experts.

**3.5 A reanalysis and discussion of Blodgett's results**

**3.5.1 Part 1: Following Blodgett**

Figure 6b shows the absolute (unsigned) errors from Figure 6a after all other aspects of data cleaning and with the questionable black and grey control conditions removed from the plot. There was a general reduction in the overall magnitude of perceptual error (by 9.2 deg), and a reduction in the variance (the SD reduced by 3.7 deg), but no obvious pattern of perceptual errors emerged. To get an overall picture across the twelve camouflage designs, we collapsed the results across skyscape while respecting the sign of perceptual errors before any of the averaging (see S3 for further details) and plotted these in Figure 6c (completing our data cleaning). Still there was little of interest to see, but the overall effect size reduced by a further 4.3 deg. Inspection of the twelve individual designs (S3) also revealed nothing further about visual misperceptions.

**3.5.2 Part 2: Analysis of individual trials across four different factors**

Our next step was to perform a detailed analysis at the individual trial level. We did this to investigate potential effects of observer, skyscape, ship class and ship colour. Although Blodgett (1919) expressed interest in these four factors, he provided no formal comparisons. Figure 7a shows perceived direction against physical direction for all cleaned data (subject to mirror reversal as appropriate; see earlier section), and is colour coded for the three/six observers. The black diamonds show the two cluster averages (see figure caption for arithmetic details). The three observer groups overlapped substantially and t-﻿tests (not shown) on each of the three possible comparisons within each of the two clusters of signed errors (six t-tests) confirmed there were no significant differences between the observers (no value of *p* (two-tailed) was less than 0.33). We used this homogeneity to justify pooling experimental trials across observer (treating the three different observer entries as a single random variable) when looking for differences in the other factors below.


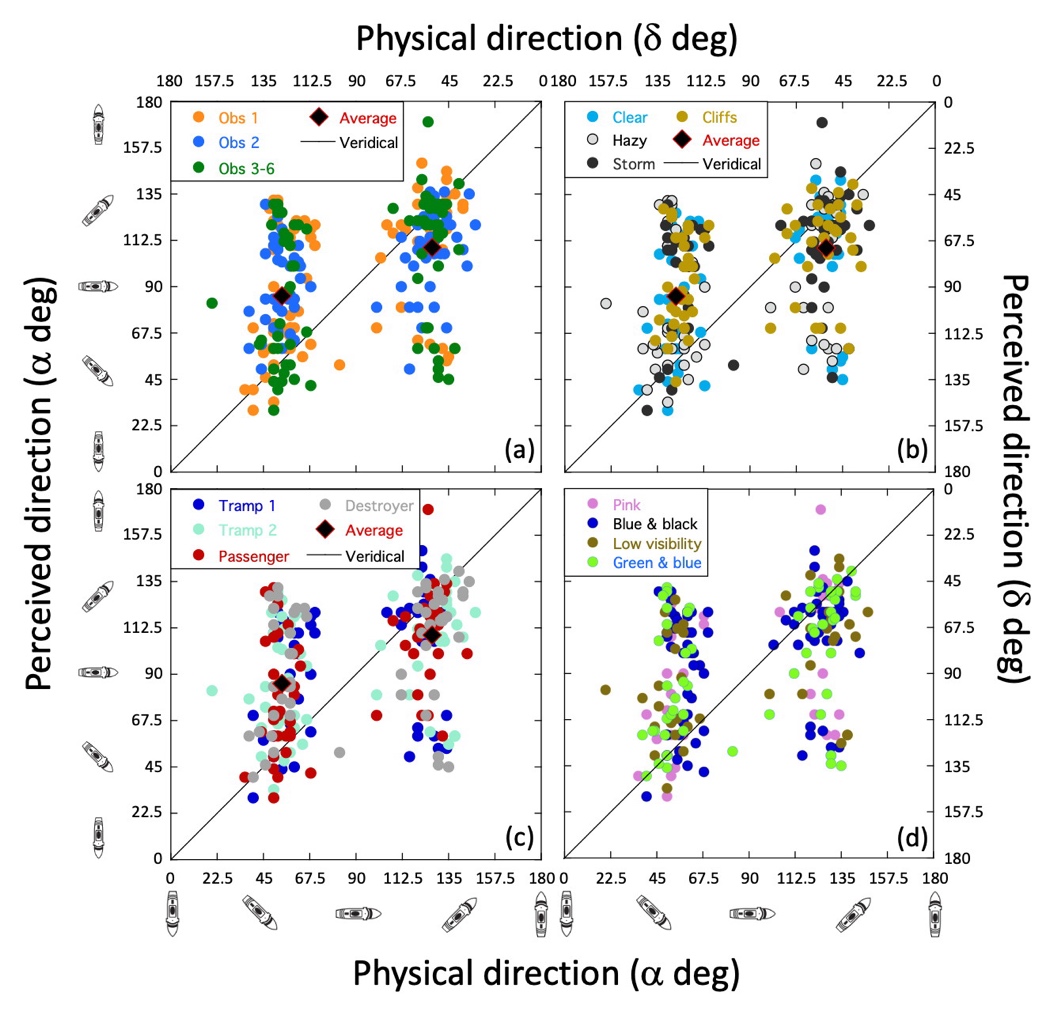


Figure 7. Cleaned results for perceived direction as a function of physical direction. The axis icons show the direction of the target ships relative to the viewpoint of the observer. The labels are for each of the two direction conventions (target angle, α, and compass angle, δ) described in Figure 4. The diagonal lines in each panel are the contours of veridicality. Deviations of data points from these contours indicate signed perceptual errors (according to the target angle axis). In (a, b, c), each circle is the result of an individual trial, and all valid trials are plotted (see *Data cleaning*), including mirror reversals (see S3). The black diamonds are (unweighted) averages of the data cluster means across mirror reversals (see S4). The data set is the same across each of these three panels. The symbol colours denote grouping by (a) observer, (b) skyscape and (c) ship class. The results in (d) are for camouflage colour and exclude ship design 11 (see text for details). Within each panel, there were no statistically significant differences for the factors depicted. In these plots, the signed differences between the data points and the contours of veridicality denote the perceptual error distributions.

Figure 7b is the same as Figure 7a but colour coded for the four different skyscapes. Again, there was substantial overlap across conditions and t-tests (not shown) confirmed there were no significant differences in the six available comparisons within each of the two clusters of signed errors (twelve t-tests). The lowest value of *p* (two-tailed) was 0.12, for a mean difference of 10.5 deg (hazy > storm) for target angles between 90 and 180 deg.

Figure 7c is the same as Figure 7a but colour coded for the four different ship classes. Once more there was substantial overlap across conditions and t-tests (not shown) confirmed there were no significant differences in the six available comparisons within each of the two clusters of signed errors (twelve t-tests). The lowest value of *p* (two-tailed) was 0.088, for a mean difference of 12.5 deg (Tramp 1 (designs 1-3) > Passenger (designs 7-9)) for target angles between 0 and 90 deg.

Finally, Figure 7d is similar to Figure 7a but colour coded for four different colour combinations in the camouflages (accepting that the colours now might not be as they were in 1919; see text around Figure 3). These groupings were determined by our sixteen naive participants after performing the control experiment below (see also S5 & S6). They were given twelve rectangular pieces of paper, each containing one of the coloured designs in Figure 3, and asked to sort them into four piles, each containing any number of ships but associated by the camouflage colours and shades. They were told to ignore the shape (class) of the ship and the form of the patterns. There was marked consistency across participants for eleven of the twelve designs, and we labelled the groupings that emerged as follows: Pink for designs 1 and 7; Blue & Black for designs 2, 3, 4 & 9; Low Visibility, for designs 5 & 6^[[1]](#footnote-1)^; and Green & Blue, for designs 8, 10 & 12 (see Figure 3 for design labels). Design 11 was omitted because there was inconsistency in its grouping across participants (see Supplementary Material S6 for further details). Figure 5d shows substantial overlap across these colour groups and t-tests (not shown) confirmed there were no significant differences in the six available comparisons within each of the two clusters of signed errors (twelve t-tests; no value of *p* (two-tailed) was lower than 0.44). This is consistent with Bittinger & Hulburt's (1936a) appraisal and development of WWI dazzle designs, claiming that "[n]o evidence could be discovered that colo[u]rs added to the effectiveness of the designs, and therefore all the new designs presented here employ only shades of gray...". We know of no quantitative support for this statement until now, though a definitive claim would require making comparisons between coloured designs and achromatic versions of those same designs.

In sum, with a liberal approach to the formal statistics, we looked for differences across observers, skyscapes, ship classes and camouflage colours by performing 42 two-tailed t-tests and found no significant effects, even without Bonferroni correction. This does not mean there are no effects for these factors in general, but there is no evidence for mean differences in Blodgett's cleaned data under the constraints of his reporting and his experimental design.

1. Although Blodgett (1919) was interested primarily in dazzle camouflage, his writing suggests that that these two designs also employed low visibility techniques (see S1). [↑](#footnote-ref-1)
