# Supplementary material for: Blodgett's (1919) “Ship camouflage” 105 years on: A misperception of dazzle perception revealed and redressed
Source: Iperception. 2025 Mar 14;16(2):20416695241312316. doi: 10.1177/20416695241312316 (PMC11909666; doi:10.1177/20416695241312316)
Supplement: sj-docx-13-ipe-10.1177_20416695241312316 - Supplemental material for Blodgett's (1919) “Ship camouflage” 105 years on: A misperception of dazzle perception revealed and redressed [file sj-docx-13-ipe-10.1177_20416695241312316.docx]

**Meta data document for Meese & Strong (2025) *i-Perception*, on Blodgett (1919)**

Blodgett's (1919) "Ship Camouflage" 105 years on: A dazzling misperception of dazzle perception revealed and redressed

----

This document refers to five data files.

1. Blodgett results (pre-cleaning)

This file contains a transcription of Blodgett's original raw data (perceived and actual directions (in degrees) of model ships viewed through a periscope) now in editable form. These data are how we found them in his original public domain file. Results for each of the 12 camouflage designs and skyscapes (left hand labels) contain three different average columns. The central one ('Blodgett Average') is a transcription of Blodgett's reported averages. The column to the left of this is our own calculation of the averages of Blodgett's results. Both of these columns are used in our own report. The column to the right of this is a weighted average based on the assumed number of participants involved in each of Blodgett's measures. This data column is not used in our report.

Some data entries in this document are set in bold and some in orange. These are explained within the data file.

2. Blodgett results (pre-cleaning reinstate)

This is the same data file as the one above but with 9 data entries highlighted in blue. These are the data initially excluded from our analysis in the main report but reinstated in the supplementary material (S4) for further analysis.

3. Blodgett results (post-cleaning)

These are the results from the first file above, with the errors identified by us removed. This file contains only raw data. These are the data we used for the main analyses in our own paper.

4. Colour grouping results

This is an editable results file for the experiment on colour grouping that we report in supplementary material S6. The entries are identical to those in S6 where details can be found.

5. New control experiment results

This file contains the raw data for the 'new control experiment' reported in the main body of our paper. ('New' because it was conducted in 2024, compared to the original in 1919.) The columns are for 16 naive observers. The entries are the perceived directions of ships (using the compass convention as described in the paper) in dazzle and grey camouflage at the true directions indicated by the lefthand columns. See data file and our own paper for further details.

**Reference**

Blodgett, L. S. (1919) "Ship camouflage," MIT thesis, B.S., Dept. of Naval Architecture and Marine Engineering. Department of Distinctive Collections, MIT Libraries, Cambridge, Massachusetts.
